# Transcription Adaptation during *In Vitro* Adipogenesis and Osteogenesis of Porcine Mesenchymal Stem Cells: Dynamics of Pathways, Biological Processes, Up-Stream Regulators, and Gene Networks

**DOI:** 10.1371/journal.pone.0137644

**Published:** 2015-09-23

**Authors:** Massimo Bionaz, Elisa Monaco, Matthew B. Wheeler

**Affiliations:** 1 Laboratory of Stem Cell Biology and Engineering in the Department of Animal Sciences, University of Illinois at Urbana-Champaign, Urbana, Illinois, United States of America; 2 Institute for Genomic Biology, University of Illinois at Urbana-Champaign, Urbana, Illinois, United States of America; University of Wisconsin-Madison, UNITED STATES

## Abstract

The importance of mesenchymal stem cells (MSC) for bone regeneration is growing. Among MSC the bone marrow-derived stem cells (BMSC) are considered the gold standard in tissue engineering and regenerative medicine; however, the adipose-derived stem cells (ASC) have very similar properties and some advantages to be considered a good alternative to BMSC. The molecular mechanisms driving adipogenesis are relatively well-known but mechanisms driving osteogenesis are poorly known, particularly in pig. In the present study we have used transcriptome analysis to unravel pathways and biological functions driving *in vitro* adipogenesis and osteogenesis in BMSC and ASC. The analysis was performed using the novel Dynamic Impact Approach and functional enrichment analysis. In addition, a *k*-mean cluster analysis in association with enrichment analysis, networks reconstruction, and transcription factors overlapping analysis were performed in order to uncover the coordination of biological functions underlining differentiations. Analysis indicated a larger and more coordinated transcriptomic adaptation during adipogenesis compared to osteogenesis, with a larger induction of metabolism, particularly lipid synthesis (mostly triglycerides), and a larger use of amino acids for synthesis of feed-forward adipogenic compounds, larger cell signaling, lower cell-to-cell interactions, particularly for the cytoskeleton organization and cell junctions, and lower cell proliferation. The coordination of adipogenesis was mostly driven by Peroxisome Proliferator-activated Receptors together with other known adipogenic transcription factors. Only a few pathways and functions were more induced during osteogenesis compared to adipogenesis and some were more inhibited during osteogenesis, such as cholesterol and protein synthesis. Up-stream transcription factor analysis indicated activation of several lipid-related transcription regulators (e.g., PPARs and CEBPα) during adipogenesis but osteogenesis was driven by inhibition of several up-stream regulators, such as MYC. Between MSCs the data indicated an ‘adipocyte memory’ in ASC with also an apparent lower immunogenicity compared to BMSC during differentiations. Overall the analysis allowed proposing a dynamic model for the adipogenic and osteogenic differentiation in porcine ASC and BMSC.

## Introduction

The mesenchymal stem cells (MSC) are able to differentiate into multiple cell lineages [1], secrete numerous growth factors and cytokines with important functions in tissue regeneration [2], are immune privileged [3], and secrete immunomodulatory factors [4, 5] which make them excellent candidates for tissue replacement therapies. Bone marrow-derived mesenchymal stem cells (BMSC) are considered the gold standard for tissue engineering applications and disease treatments among MSC [6, 7]. The MSC were originally isolated from bone marrow [8], but they are present in many tissues due to their perivascular location [9]. One of the most interesting tissues for the isolation of MSC is adipose. The quantity and accessibility of subcutaneous adipose tissue in humans and other species makes it an attractive alternative to bone marrow as a source of adult stem cells [10–12]. As previously reported [13, 14], besides non-human primates, the pig can be considered an ideal animal model for initial studies exploring human MSC therapeutic applications. In addition, the porcine adipose derived stem cells (ASC) can be easily harvested, isolated, expanded and differentiated *in vitro* [13, 15, 17].

We have previously characterized porcine BMSC and ASC during adipogenic and osteogenic differentiation in a 2-dimensional culture system and we observed some morphological differences, particularly during osteogenesis [17]. In order to investigate the observed differences between the two MSC prior and during osteogenesis and adipogenesis we also performed a direct transcriptomic comparison between the two MSC types using a large microarray dataset [14]. In the same experiment we have also investigated the differences between osteogenic and adipogenic differentiation. The low number of differentially expressed genes (**DEG**) between the two MSC prior to differentiation highlighted the large similarity between the cell types. We observed an abundant expression of genes involved in immunomodulation, angiogenesis, and collagen formation [14] for both cell types. During both types of differentiation, few genes were differentially expressed between the two MSC. The functional analysis of those DEG indicated that ASC had a larger lipogenic signature compared to BMSC, while BMSC had a stronger proliferative capacity compared to ASC. The ASC were observed to have a greater angiogenic signature during adipogenesis compared to BMSC [14]. Between the differentiation types our data clearly suggested a pivotal role of PPAR signaling, a consistent greater lipogenesis and a greater angiogenic capacity of both MSC during adipogenesis compared to osteogenesis [14]. Inversely, when osteogenesis was compared to adipogenesis there was greater proliferation during the earlier phases of differentiation and a larger migratory capacity, involving cytoskeleton reorganization, as differentiation progressed. Our analysis also highlighted a pivotal role of G-proteins in determining the early stages of osteogenic differentiation of MSC [14].

The above results came from the analysis of the differentially expressed genes between comparisons but the real dynamic adaptations of the transcriptome were not analyzed. The functional analysis of the previous manuscript was performed using the enrichment analysis or overrepresented approach (ORA) [18]. The ORA is a robust and reliable approach in order to capture the most important biological terms in gene lists; however, it presents serious limitations when used for analysis of time course experiments [18, 19]. The ORA can be used in time course experiments after reduction of the dataset using cluster or principal components analyses. The cluster and/or principal components analyses are very useful in order to investigate if changes in genes coding for proteins involved in particular pathway or other biological terms are highly coordinated. This also allows for uncovering transcription factors involved in the regulation of genes with similar pattern in expression. Those approaches, however, do not allow investigation/visualization of the dynamic changes in the pathways or other biological events during the whole time course or between multiple treatments alone or in combination.

The molecular processes involved in MSC adipogenesis and osteogenesis both *in vivo* [20] and *in vitro* have been studied and reviewed [21–24]. It is well established that PPARγ is the master regulator of adipogenesis and it also has a crucial, although negative, role during osteogenesis [14, 22–25]. The Wnt signaling system plays a pivotal role in the osteogenic and adipogenic fate of MSC, with the canonical Wnt-β-catenin signaling being in favor of the osteogenesis (and being inhibitory toward adipogenesis) and the non-canonical Wnt pathway being in favor of the adipogenesis (and being inhibitory toward ostegenesis) [24]. Even though the signaling pathway(s) determining adipogenic or osteogenic fate in porcine BMSC and ASC is likely highly conserved between human, mouse, and pig, this has not been experimentally established.

With the purpose of complementing prior studies [14, 17], the aim of the present investigation was to uncover pathways, biological functions, and transcription factors involved in determining the osteogenic and adipogenic fate of ASC and BMSC. This was accomplished by performing a large functional analysis of microarray data using three different but interconnected approaches: (1) a functional analysis of KEGG pathways and Gene Ontology (GO) terms using the novel DIA and the classical enrichment analysis, (3) analysis of up-stream transcription factors and their estimated activation/inhibition, and (2) a *k*-mean cluster analysis in association with enrichment analysis and scrutiny of networks in order to determine co-regulated functions and uncover transcriptional factors that more significantly overlap with genes in *k*-mean clusters.

## Results and Discussion

### Overall transcriptome perturbation during differentiation

**Fig 1:**
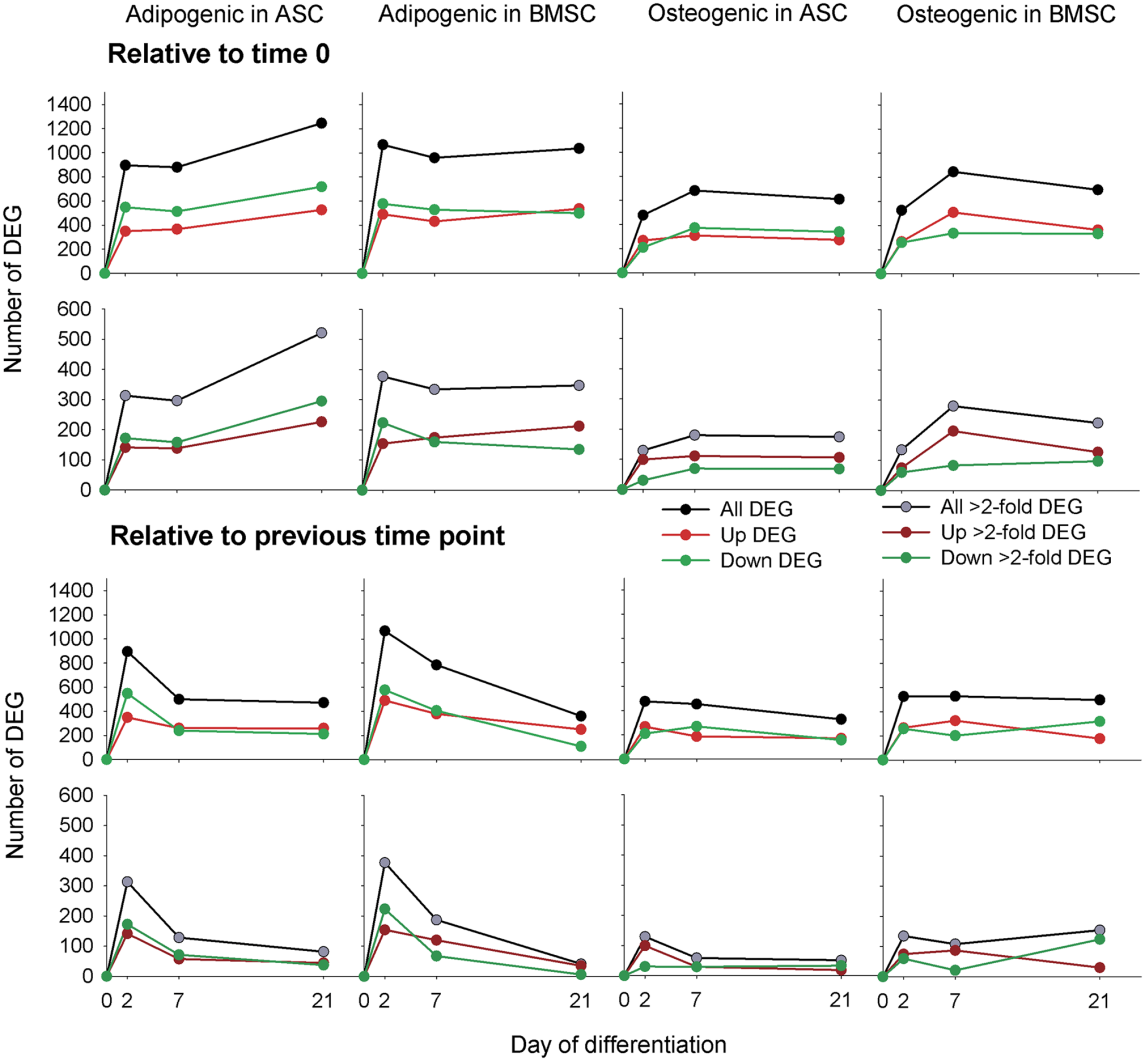
Number of differentially expressed genes during adipogenic and osteogenic differentiation in BMSC and ASC. Upper panels denote number of differentially expressed genes (DEG; FDR ≤ 0.05 and P-value between comparison ≤ 0.05) in all time points during differentiation compared to time 0 (i.e., prior differentiation) without fold-change cut-off (top panels) or with 2-fold change cut-off (lower panels). Lower panels denote the number of DEG between consecutive time points with or without 2-fold change cut-off.

Complete dataset with fold-change and statistical results are reported in S1 File. The overview of the pattern of the 2,200 DEG with an overall false discovery rate or FDR ≤ 0.05 for time × differentiation × cell type effect is shown in S1 Fig The number of DEG (FDR ≤ 0.05 for time × differentiation × cell type effect plus P-value≤ 0.05 between comparisons) in each MSC undergoing osteogenic and adipogenic differentiation is shown in Fig 1.

The adipogenic induction had a greater effect on the transcriptome compared to the osteogenic induction with a similar effect between the two MSC, but with a larger number of DEG in BMSC at 2 vs. 0 days of differentiation (2dd) compared to ASC and a larger number of DEG in ASC compared to BMSC at 21dd (Fig 1). The ASC had a larger number of down-regulated vs. up-regulated genes compared to BMSC (Fig 1). The osteogenic differentiation had an overall similar number of DEG between the two MSC, but with a slightly larger number of DEG in BMSC vs. ASC, particularly at 7dd. The osteogenic differentiation had also a larger number of up-regulated vs. down-regulated genes in BMSC vs. ASC. When the DEG between consecutive time points was analyzed (Fig 1) it was evident that the larger change in expression happened at the beginning of the two differentiations (i.e., at 2dd), particularly for the adipogenic differentiation. Significant changes in transcriptome during the early phases of *in vitro*-induced adipogenesis were observed also in T3T cells (i.e., mouse) [26] and in human adipocyte stem cells [27].

It appears from the above data that the adipogenic differentiation requires a greater transcriptomic perturbation to take place compared to the osteogenic differentiation. The fact that adipogenesis is highly regulated at the transcriptional level has been known for at least a decade [28]. In addition, the data in Fig 1 also showed that adipogenesis is featured by a large number of down-regulated DEG and this was more pronounced in ASC, while the osteogenesis is featured by a larger number of up-regulated DEG, particularly for the BMSC. This indicates that in order to induce the adipogenic signature the expression of most of the genes needed to be reduced, particularly for the ASC. The pattern of the number of DEG also showed that the transcriptomic changes related to adipogenesis happened in larger magnitude and at earlier time points compared to the osteogenesis. Another feature suggested by this analysis was the slightly larger number of DEG in BMSC vs. ASC during the early phases of adipogenesis, indicating either a larger transcription sensitivity of BMSC or, inversely, the need for a perturbation of a larger number of transcripts to induce adipogenesis. However, the most striking observation remains the high similarity in the number of DEG during the same differentiation between the two MSC and the obvious difference between the two differentiation types. This last observation supports, as previously concluded [14, 17], an overall large similarity between porcine ASC and BMSC.

### KEGG pathway analysis using the Dynamic Impact Approach (DIA)

**Fig 2:**
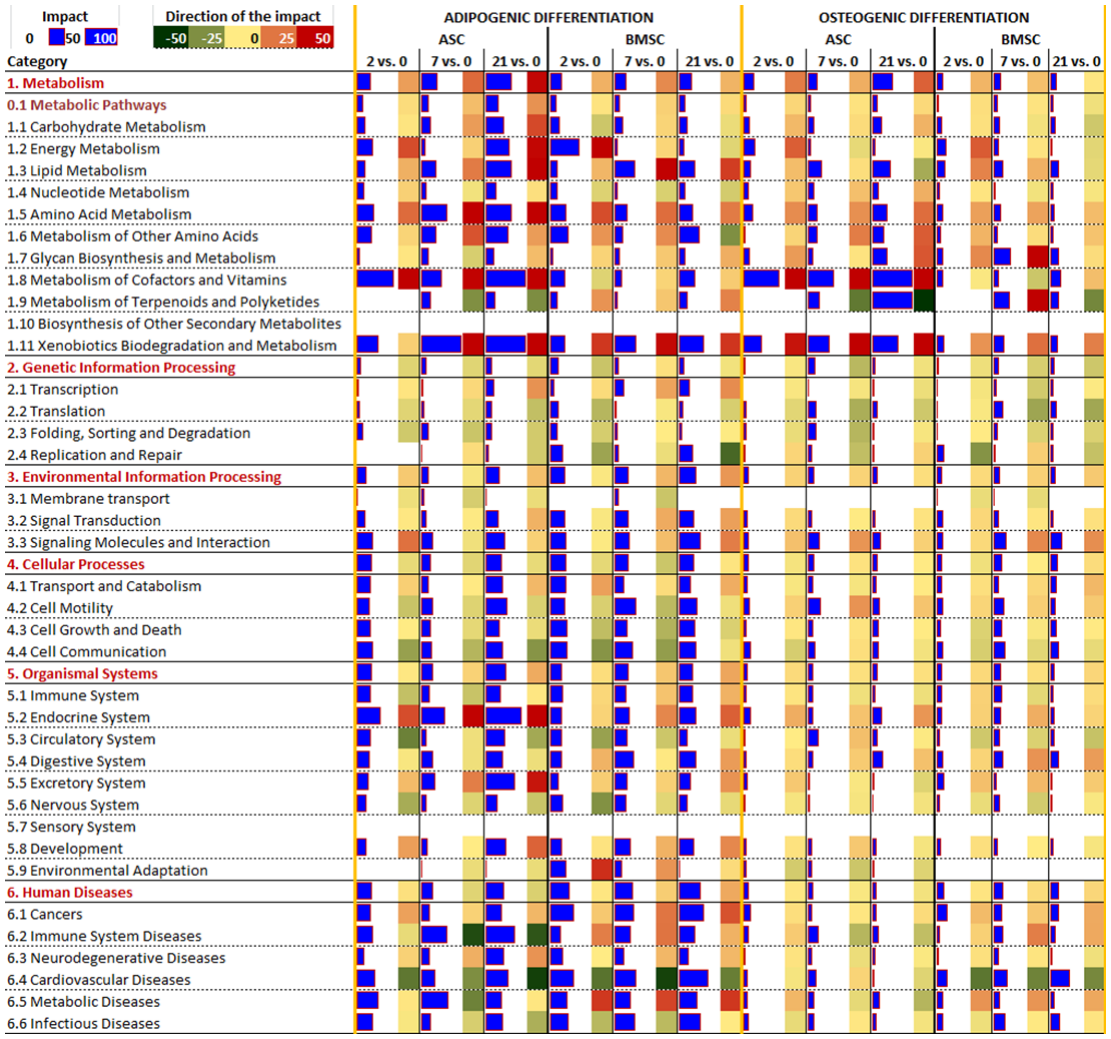
KEGG pathways: overall dynamic adaptation of the main categories and sub-categories of pathways. Overall impact and direction of the impact for the main KEGG pathway categories (red font) and sub-categories (black font) as calculated by the Dynamic Impact Approach. Reported are the “Impact”, i.e. the numerical effect or impact on the pathway, and the “Direction of the impact”, i.e. the overall estimated effect on the pathway (red = activated, i.e. the category or sub-category of pathways is estimated to be overall induced; green = inhibited, i.e. the category or sub-category of pathways is estimated to be overall reduced).

The DIA [19] was used to analyze the dynamic adaptation of the pathways during the adipogenic and osteogenic differentiation. The summary view of the main KEGG pathways categories is reported in Fig 2. From the figure it is clear an overall induction of metabolism during both differentiation types but with a larger induction during adipogenesis vs. osteogenesis and in ASC vs. BMSC. Adipogenesis was featured by an induction, although slight, of the main categories of pathways ‘Environmental Information Processing’ and ‘Organismal Systems’ and a minor inhibition of ‘Cellular Processes’ and ‘Genetic Information Processing’. ‘Human disease’ KEGG pathway category was also highly impacted during adipogenesis. The osteogenesis was featured by an overall activation of metabolism, and an overall inhibition of ‘Genetic Information Processing’.

The larger induction of metabolism during adipogenesis was mainly due to pathways related to few sub-categories of KEGG pathways such as lipid and amino acid metabolism and metabolism of xenobiotics, cofactors, and vitamins. The ‘Metabolism of cofactors and vitamins’ was strongly induced in ASC compared to BMSC unregard of differentiations.

**Fig 3:**
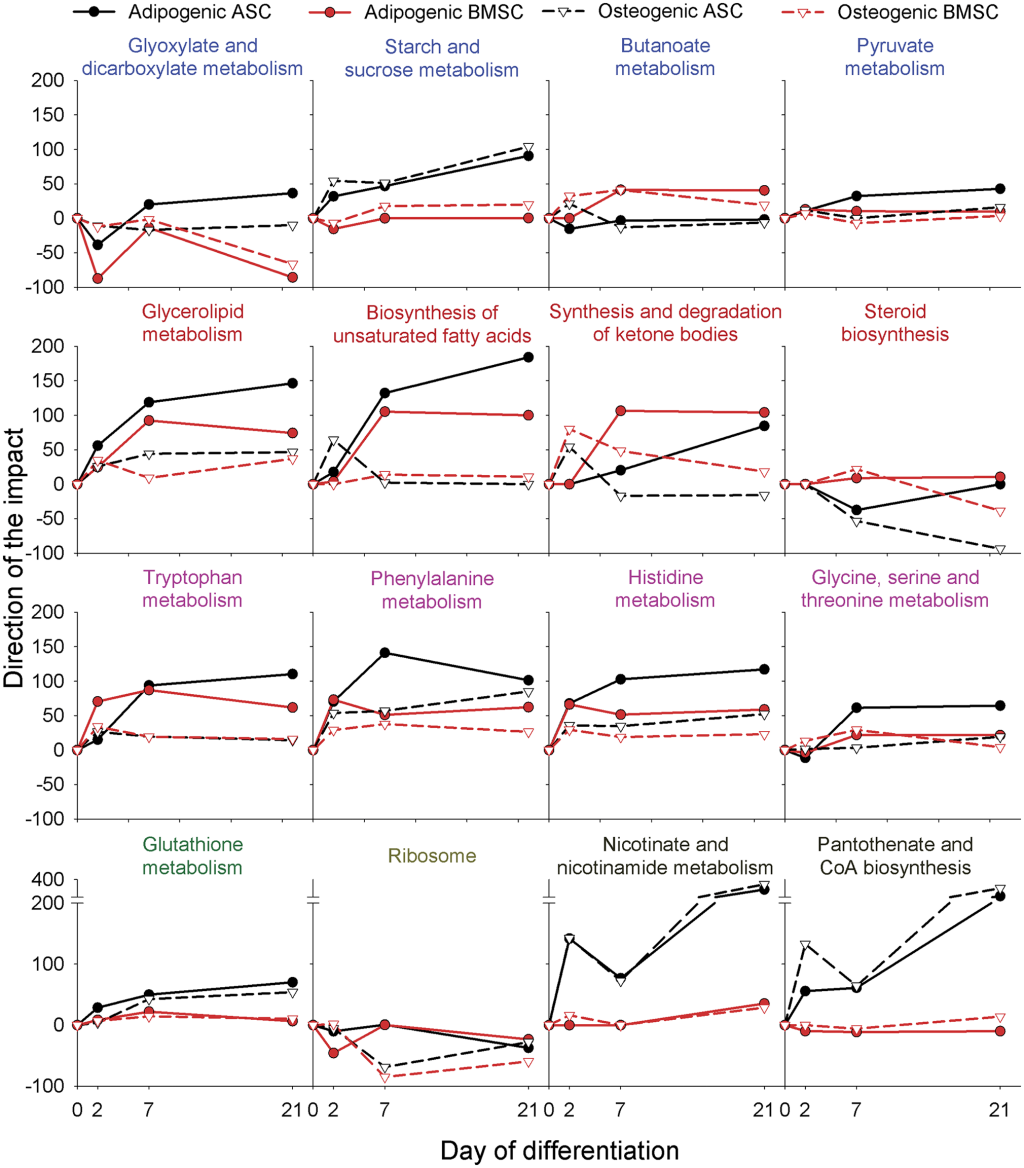
KEGG pathways related to metabolism. Shown are the direction of the impact of the most impacted metabolic-related pathways in each time point relative to pre-differentiation of adipogenic or osteogenic differentiation in ASC and BMSC. **Blue** font denotes carbohydrate metabolism-related pathways; **dark red** font denotes lipid metabolism-related pathways; **purple** font denotes amino acid metabolism-related pathways; **green** font denotes other amino acid metabolism-related pathways; **dark yellow** font denotes translation-related pathways; **black** font denotes cofactors and vitamins metabolism-related pathways (see details for all pathways in S2 File, sheet ‘KEGG Pathways’).

The ‘Carbohydrate metabolism’, sub-category of the metabolic pathways, was not strongly affected by the two differentiation methods (Fig 2). The majority of the pathways related to this sub-category, with the exception of the ‘Butanoate metabolism’, had a stronger induction in ASC compared to BMSC in either differentiation (Fig 3).

The most impacted pathways related to the ‘Lipid metabolism’ sub-category were the ‘Biosynthesis of unsaturated fatty acids’ and formation of triacylglycerol (i.e., ‘Glycerolipid metabolism’). These pathways were evidently more induced during adipogenesis compared to osteogenesis and in ASC compared to BMSC at 21dd (Fig 3). Those data support prior conclusions [14, 17] and confirm previous morphological results where large lipid droplets containing triglyceride were accumulated by both cells types during adipogenesis but with a larger accumulation in ASC [17].

Interestingly, the synthesis of steroids was not induced during adipogenesis and was inhibited during osteogenesis (Fig 3). An earlier analysis, using the same transcriptomic study but comparing the DEG between the two MSC during differentiations and using an enrichment analysis approach, suggested a larger importance of sterol biosynthesis in BMSC compared to ASC during early osteogenesis [14]. The results from the DIA of the present analysis support those earlier conclusions (Fig 3). Interestingly, the inhibition of cholesterol synthesis appears to be important in order to reduce bone loss [29] and enhance bone formation [30]. In support of this, it has been recently reported that the inhibition of osteogenesis in human MSC by chlorate is featured by a consistent increase in cholesterol synthesis [31]. Overall, in view of the above observations and of the present results, the decline of cholesterol synthesis during the late stage of osteogenic differentiation can be considered a consistent feature in both cells types but more pronounced in ASC (Fig 3).

The amino acid (AA) metabolism was also overall more induced during adipogenesis compared to osteogenesis, particularly for ASC (Fig 3). The metabolism of several AA, including Trp, His, Phe, Gly, Ser, and Thr and ‘Glutatione metabolism’ were among the most affected (Fig 3). Amid the AA metabolism, the ‘Tryptophan metabolism’ was the most induced during adipogenesis compared to osteogenesis (Fig 3). A detailed visualization of the pathway in ASC and BMSC at 7dd (S2 Fig) suggests that the MSC used Trp to produce several indole acetate-type molecules directly from Trp or passing by serotonin intermediate. This is probably a phenomena induced by indomethacin [32], which was added in large concentration in the adipogenic cocktail in the present experiment [17]. The indomethacin and the serotonin metabolites (that are also derived from Trp) have been shown to activate PPARγ and adipogenesis in human cells [32, 33]. Interestingly, also the induction of ‘Phenylalanine metabolism’ suggests the use of Phe for the production of the metabolite phenylacetate (S3 Fig), which has been observed to be an activator of adipogenesis in human MSC [34]. Those observations indicate that the MSC induced toward adipogenesis increase the metabolism of AA in order to produce intermediate metabolites that have a feed forward effect on further inducing adipogenesis. Those findings are supportive of our previous suggestion about the progressive induction of differentiation in ASC by provision of additional adipogenic signaling molecules produced by differentiated cells [17].

The ‘Metabolism of Cofactors and Vitamins’ was the most impacted sub-category of pathways (Fig 2). Interestingly, most of the pathways in these subcategories were strongly affected by the type of MSC but not by the type of differentiation. With the exception of ‘Terpenoid backbone biosynthesis’ that was mostly affected by differentiation (S2 File, sheet ‘KEGG pathways’), the ‘Nicotinate and nicotinamide metabolism’, ‘Pantothenate and CoA biosynthesis’, and ‘Riboflavin metabolism’ were only mildly affected by the type of differentiation, with an overall larger induction during osteogenesis and were consistently strongly induced in ASC during either differentiation path but not in BMSC (Fig 3 and S2 File, sheet ‘KEGG pathways’). The pattern of those pathways was however determined by a large change of one gene, ectonucleotide pyrophosphatase/phosphodiesterase 1 (*ENPP1*). The product of this gene has been shown to be essential for the control of bone mineralization [35]. Further, its overexpression in adipose tissue also induces insulin resistance both in the adipocytes and at the systemic level [36]. Thus, our findings of a consistent high expression of *ENPP1* in ASC during both types of differentiation appear to be supportive of an essential role of this gene in both bone mineralization and adipose tissue maturation. The insulin resistance effect of the product of *ENPP1*[36] might be due to the adipocytes trying to prevent lipid overload by reducing glucose uptake that in turn allows regulating lipogenesis.

The ‘Xenobiotic biodegradation and metabolism’ pathway was greatly induced during adipogenesis (Fig 2) due to large activation of P450-related pathways (S2 File), particularly for ASC. An important role of P450 in white adipose tissue in human has been reported [37]. The activation of P450 in human adipose stem cells retard adipogenesis thorugh the increased production of epoxyeicosatrienoic acid [38]. A detailed visualization of the P450 pathways during adipogenic differentiation in ASC in the present work (S4 Fig) revealed that the epoxyeicosatrienoic acid production was not induced during adipogenesis but the production of many other xenobiotics metabolites. It is not clear at the present the significance of this finding.

**Fig 4:**
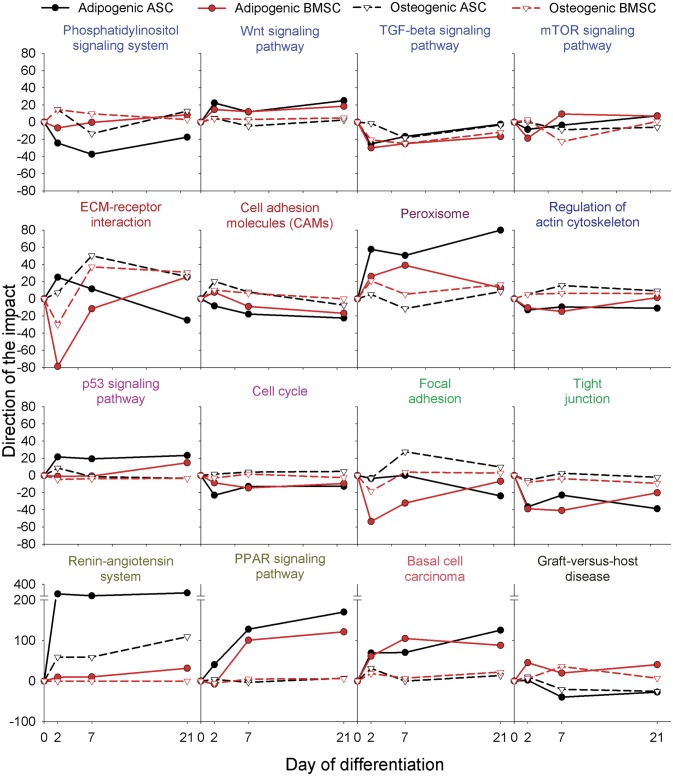
KEGG pathways related to other categories. Shown are the direction of the impact of the most impacted non metabolic-related pathways in each time point relative to pre-differentiation of adipogenic or osteogenic differentiation in ASC and BMSC. **Blue** font denotes signal transduction-related pathways; **dark red** font denotes signaling molecules and interaction-related pathways; **dark purple** font denotes transport and catabolism-related pathways; **dark blue** font denotes cell motility; **purple** font denotes Cell growth and death-related pathways; **green** font denotes cell communication-related pathways; **dark yellow** font denotes endocrine system-related pathways; **red** font denotes cancers-related pathways; **black** font denotes immune system diseases-related pathways (see details for all pathways in S2 File, sheet ‘KEGG Pathways’).

The ‘Ribosome’ KEGG pathway was evidently inhibited during the beginning of osteogenesis (Fig 3). Interestingly, data also indicated a slight inhibition of ‘mTOR pathway’ during osteogenesis and an evident inhibition of ‘Cell cycle’ during adipogenesis in both MSC (Fig 4). The inhibition of cell cycle (also supported by the ‘p53 signaling pathway’; Fig 4) indicate a reduction of proliferation during adipogenesis compared to osteogenesis. Those data are supported by our previous observation of an increase in number of cells during early phases of osteogenesis and a decrease during adipogenesis [14]. An overall decrease of phosphorylation of pathways involved in protein synthesis and cell proliferation was recently observed during the early phases of human BMSC *in vitro* osteogenic differentiation [39]. The decrease in phosphorylation of proteins involved in the regulation of protein synthesis (e.g., mTOR) results in a reduction of mRNA translation. Those observations, together with our data, indicate that protein synthesis was rather inhibited during osteogenesis, despite the fact that a large amount of secreted proteins are needed for extracellular matrix formation [40]. Protein synthesis is an important phenomenon during bone formation and an inhibition of protein synthesis reduces bone formation *in vivo* and *in vitro* [41]. However, protein synthesis is, energetically speaking, a very costly biological phenomenon and an inverse relationship between cell proliferation and protein synthesis has been observed [42, 43]. As previously reported [14] we have detected an increase in cell proliferation during osteogenesis but a decrease during adipogenesis. In this regard, the suggested decrease in protein synthesis might have allowed for a larger availability of energy for cell proliferation during osteogenesis. For adipogenic differentiation it appears that energy was sequestered for the accumulation of triglycerides, because we did not observe an increase in cell proliferation [14]. As also suggested previously [39], the decrease in protein synthesis as a way to spare energy might be a consequence of reduced energy availability due to glucose and/or serum depletion in the culture medium; however, for the present experiment we have used high-glucose media [17], suggesting that other factors might be more limiting. Protein synthesis was apparently not affected by adipogenesis, if not slightly inhibited in the early phases of differentiation, particularly for BMSC (Fig 3). This observation is in contrast to an early observation of a large increase in expression of several ribosomal proteins in 3T3-L1 cells after 6h of adipogenesis [26], observation recently strongly confirmed [44]. A complete overlap of our data with the above paper is impossible due to the different time of cell harvesting (the earlier harvesting in our case was 2h); however, all the ribosomal proteins that were observed to be up-regulated in 3T3 cells were either down-regulated or unaffected in porcine ASC at 2 day of adipogenesis in porcine ASC (e.g., *RPL7A*, *RPL6*, and *EIF4B*, see S1 File). These data may indicate a species-species differences; however, lack of an important role of ribosomal proteins during late stages (i.e., >1 day) adipogenesis in human ASC can be extrapolated by several works [45, 46]. Furthermore, the observation of a shift of several mRNA toward polyribosome during asipogenesis in 3T3-L1 cells [26] (i.e, increased translation) highlights an interpretative limitation in using only transcriptomics data.

Signaling pathways and networks drive any type of cellular differentiation. Interestingly, among the most impacted pathways were the ones related to cell-to-cell signaling (e.g., ‘ECM-receptor interaction’) and the one related to endocrine system (Fig 2) as the most impacted KEGG pathway was ‘Renin-angiotensin system’ followed by ‘PPAR signaling’ and ‘Basal cell carcinoma’ (Fig 4, S2 File, sheet ‘KEGG pathways’).

The ‘Renin-angiotensin system’ was highly impacted and induced in ASC and slightly impacted and induced in BMSC during adipogenesis. The large impact of renin-angiotensin system during adipogenesis is not surprising. The expression and secretion of angiotensinogen is known to induce pre-adipocytes differentiation and it is a marker of mature adipocytes [47].

The present analysis clearly showed a large induction of ‘PPAR signaling’ pathway due to adipogenesis (Fig 4; see S5 Fig for details of this KEGG pathway); however, it does not allow for a clear conclusion to which among the three PPAR isotypes is the most important for adipogenesis. Nonetheless, it has been well established that the activation of PPARγ is essential for the adipogenic differentiation [22, 24]. Thus our analysis appears to support the pivotal role of PPARγ in driving the adipogenic vs. osteogenic differentiation [24].

Another important pathway in driving the MSC toward the adipogenesis or osteogenesis is the Wnt signaling [24]. In our analysis the Wnt signaling was not among the most impacted pathways (see S2 File, sheet ‘KEGG pathway’); however, the impact and the induction were larger in adipogenesis compared to osteogenesis (Fig 4). The KEGG ‘Wnt signaling pathway’ at 7dd during adipogenesis and osteogenesis in BMSC is depicted in S6 Fig. From S6 Fig it is evident that both the increase in expression of Wnt genes and the increase of the components of the receptor (i.e., Frizzled) are common between the two differentiation types (also see S1 File). In both differentiation types, the genes involved in the canonical Wnt signaling were highly affected (S6 Fig). This appears to contradict the notion, based on previous findings, that the induction of Wnt/β-catenin pathway (i.e., canonical Wnt signaling) is crucial for prompting the osteogenic instead of the adipogenic fate in MSC [24]. Even though a role of the non-canonical Wnt signaling in determining the osteogenic fate of MSC has been reported [24] it is still controversial. In another study it was demonstrated that the non-canonical Wnt signaling is essential in inducing adipogenesis [48], especially due to *WNT4* and *WNT5A* expression during early differentiation. *WNT5A* was significantly induced during the early phases of both differentiations in BMSC in our experiment (S1 File).

Other pathways, such as ‘Phosphatidylinositol signaling system’ and ‘TGF-beta signaling’ were also highly impacted (Fig 4, S2 File, sheet ‘KEGG pathways’). The ‘Phosphatidylinositol signaling system’ was more induced during early osteogenesis and inhibited during adipogenesis. The pathway is linked with the focal adhesion pathway (see below) through phosphatidylinositol 3,4,5-trisphosphate and inhibition of this pathway negatively affects focal adhesion [49]. In our case the pattern of the direction of the impact between the ‘Phosphatidylinositol signaling system’ and ‘Focal adhesion’ was not similar (Fig 4).

Adipogenesis was featured by an overall inhibition of pathways related to cell-to-cell interaction and cytoskeleton regulation (including cell junction-related pathways) while osteogenesis was featured by an increase of the same pathways (Fig 4 and S2 File, sheet ‘KEGG pathways’). A larger cell-to-cell contact during osteogenesis compared to adipogenesis was also highlighted by our previous analysis [14]. Interestingly, an activation of those pathways was more evident for ASC compared to BMSC during osteogenesis (Fig 4). In our 2D *in vitro* experiment the porcine ASC tended to form large osteogenic nodules [17] through cell migration and/or a rolling of the single layer of cells to form dense nodules (see S1 Video). This observation together with the transcriptomics data indicates that cell adhesion molecules together with the regulation of the cytoskeleton might play a pivotal role in such cellular behavior. As pointed out before, the formation of nodules in ASC appears to follow the pattern of intramembranous ossification [13].

The induction of the ‘Peroxisome’ pathway during adipogenesis (Fig 4) can be related to the increase in activity of PPAR [50]; however, the number of peroxisomes and activity of their enzymes appear to be a feature of some differentiation types, as this increases significantly during cell differentiation, particularly in epithelial cells [51]. To our knowledge, no data about number and/or activity of peroxisomes are available for adipogenesis and osteogenesis in MSC.

The MSC are known to be immune-privileged particularly for the low expression or absence of major histocompatibility complex components [52]. This has been clearly demonstrated for BMSC [53], but also ASC have the same property [13, 54]. The larger inhibition of ‘Graft-versus-host disease’ during both differentiations in ASC vs. BMSC (Fig 4) and the pathways related to the sub-category ‘Immune System Diseases’ (that include also the pathway ‘Allograph rejection’) (Fig 2 and S2 File, sheet ‘KEGG pathways’) indicate a lower immunogenicity of ASC compared to BMSC during differentiations. The ASC have been shown to improve consistently the Graft-versus-host disease in several transplants [55]. Our *in vitro* data suggest ASC to be more immune privileged than BMSC. This however needs to be tested by direct *in vivo* transplant of the two MSC.

The ‘Basal cell carcinoma’ was among the most impacted KEGG pathways and was more induced during adipogenesis (Fig 4). The impact on this pathway was however due to three components: BMP, Wnt, and Frizzled (S7 Fig) that are parts of other pathways, for instance ‘Wnt signaling’ and ‘Hedgehog signaling’. In a previous analysis of the DEG between differentiation types, data indicated a larger tumorigenesis during later adipogenesis compared to osteogenesis [14]. The present data support that observation but, as also previously reported, caution should be taken in making the conclusion that adipogenesis is more tumorigenic than osteogenesis as tumor formation is not strictly a cellular phenomenon. However, further *in vivo* experiments should be run to validate such observation because there are indications that MSC from human fat or conditioned media from those cells promote tumor formation when co-transplanted with tumor cells [56].

In summary, the KEGG pathway analysis uncovered an overall increase in metabolism during adipogenesis, mostly due to lipid formation but also due to an increase in utilization of several AA. The analysis of metabolic pathways clearly depicts an adipose phenotype for the adipogenic differentiation. The osteogenic differentiation was not featured by a large change of any of the metabolic-related pathways, except a consistent decrease in steroid biosynthesis at the end of differentiation. The metabolism of cofactors and vitamins was also highly affected during differentiations, but mostly in ASC. The signaling molecule pathways analysis indicated that the induction of PPAR signaling is the most important event in determining the adipogenic fate of the porcine MSC with a concomitant involvement of Wnt and Hedgehog (S2 File, sheet ‘KEGG pathways’) signaling pathways. Interestingly, none of the signaling pathways were highly induced during osteogenesis, indicating, together with the overall lower change in gene expression (Fig 1), that the osteogenesis was less dependent on changes in gene expression and more dependent on other phenomena, likely phosphorylation. In support of this, recent phosphoproteomics analysis of human BMSC during osteogenic differentiation highlighted an important role of protein phosphorylation in driving such differentiation [39]. There is not similar data for the adipogenic differentiation to make a clear comparison; however, overall the above observations indicate a more central role of protein phosphorylation in osteogenesis compared to adipogenesis and a stronger transcriptomics adaptation at the root of the adipogenesis. Finally, the high impact of ‘Renin-angiotensin system’ and several pathways related to metabolism of cofactors and vitamins involving genes known to be adipose-specific in ASC vs. BMSC appears to indicate a retention of ‘adipocyte cell memory’ for ASC while none of the data from the KEGG pathways analysis indicate a “osteocyte cell memory’ in BMSC. The “stem cell memory” is not a new concept and it is related to epigenetic markers determined by the tissue of origin that persist in isolated cells. Human MSC retain past physical signals that determine the expression of specific differentiation markers [57] and induced pluripotent stem cells preserve an “epigenetic memory” from the original tissue that affects expression of gene and, thus, cell identity [58].

### Gene ontology analysis by the DIA and overrepresented approach by DAVID

In order to further mine the transcriptome dataset to uncover the functional dynamic changes involved in osteogenesis and adipogenesis in the two MSC we have performed the analysis of Gene Ontology (GO) categories using the DIA and the enrichment analysis by means of DAVID [59]. The GO categories include a larger amount of annotated genes compared to the KEGG pathway analysis. Complete results of all three categories of GO analysis are reported in S3 File.

With the purpose of determining the most affected terms in each condition we have computed several summaries for the DIA results including the overall direction of the impact during adipogenesis, during osteogenesis, between adipogenesis and osteogenesis, and between ASC and BMSC (all results are available in S3 File). In order to summarize the GO terms with the largest difference in the direction of the impact between adipogenesis and osteogenesis, results from the above-mentioned calculations were uploaded to REVIGO [60]. The results are available in S3 File and in several additional figures (S8 Fig and S9 Fig for GO Biological process, S10 and S11 Figs for GO Molecular function, and S12 and S13 Figs for GO Cellular component).

The overall GO analysis with the DIA indicated that many terms were similarly affected during both differentiation types (S3 File sheets ‘GO Biological process’, ‘GO Molecular function’, and ‘GO Cellular component’). Several of those terms were strongly impacted during both differentiations, such as the inhibition of protein processing and induction of genes related to the epithelial proliferation (S3 File, sheet ‘GO Biological process’).

**Fig 5:**
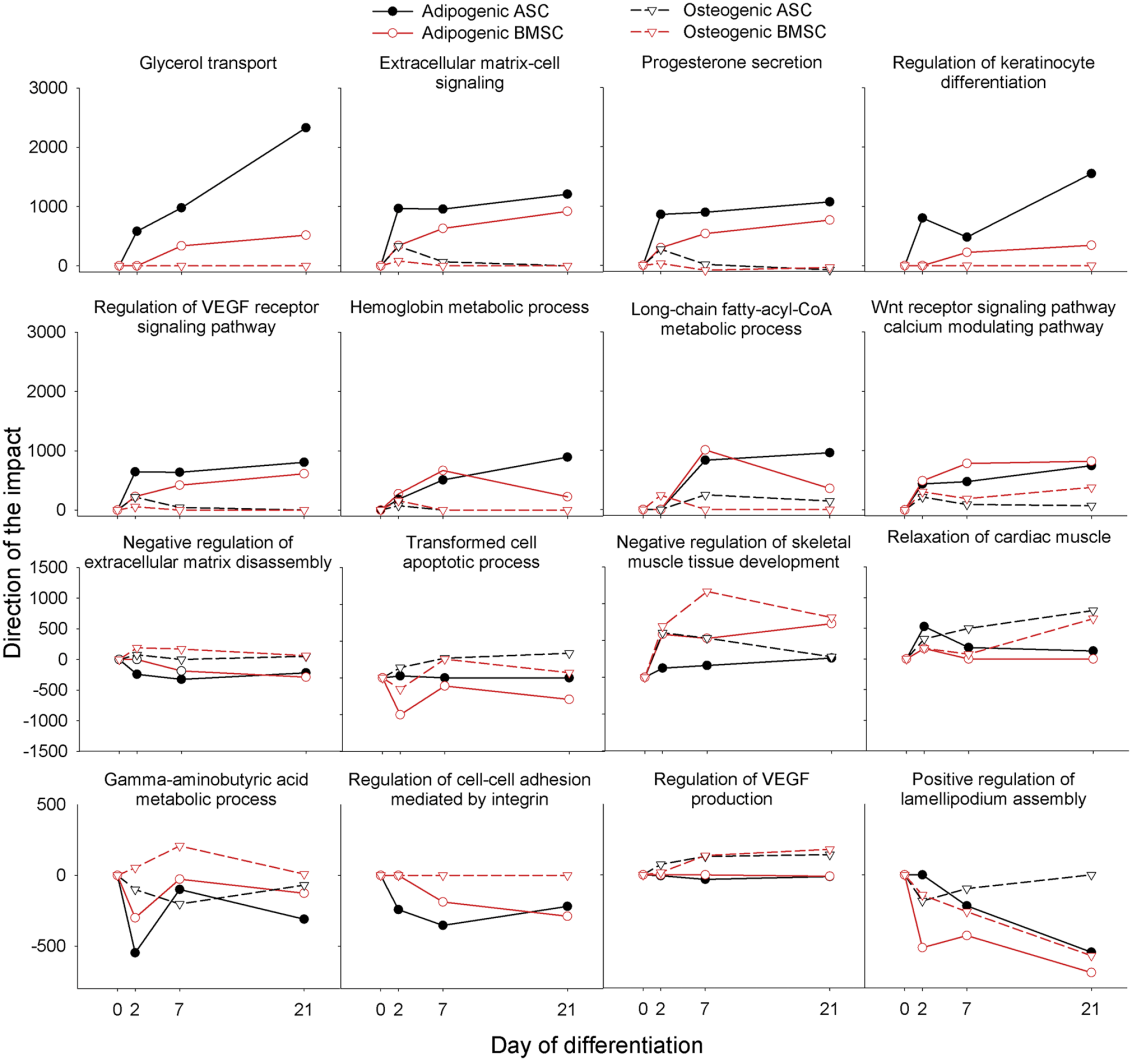
Gene ontology biological process terms. Direction of the impact of GO Biological process terms with the largest difference in the overall direction of the impact in adipogenesis compared to osteogenesis as calculated by the Dynamic Impact Approach. The two upper rows of figures report the terms with the largest overall direction of the impact in adipogenesis compared to osteogenesis. The last two rows of figures report the terms with the largest overall direction of the impact in osteogenesis compared to adipogenesis. The full results are available in S3 File.

In Fig 5 is reported the direction of the impact of the GO Biological processes (calculated by DIA) with the largest difference in the direction of the impact between adipogenesis and osteogenesis. The adipogenic differentiation, contrary to the osteogenic one, was featured by an overall very high impact of GO terms related to triglycerides synthesis with an evident large importance of glycerol transport and metabolism of fatty acids. The increase in glycerol transport, together with a lack of increase in utilization of glucose (as indicated by the KEGG pathway analysis, see Fig 3, and S2 File, sheet ‘KEGG pathway’), indicates a strong dependence of the differentiating MSC from the extracellular provision of glycerol for triglycerides synthesis. The adipogenesis was induced by addition of 1 μM of dexamethasone [17]. This compound is known in adipocytes to bind the glucocorticoid receptor and decrease expression of phosphoenolpyruvate carboxykinase (PCK1) by reducing the binding of several other factors (including CEBPs) to the promoter region of the PCK1 [61] inhibiting the glyceroneogenesis [62]. The latter appears to have a crucial role in triacylglycerol formation in mature adipocytes, particularly when there is an active lipolysis in adipocytes [62]. The suggested increase in glycerol transport and the latter observations suggest a minor role of glyceroneogenesis during adipogenic differentiation in our experimental conditions.

The adipogenesis was also featured by a large induction of extracellular matrix signaling that included secretion of hormones (indicated by ‘Progesterone secretion’, see Fig 5) and sensitivity to growth factors (indicated by ‘VEGF receptor signaling pathway’, see Fig 5). The high impact and induction in adipogenesis, but not in osteogenesis, of the ‘Wnt signaling pathway calcium modulating pathway’ (Fig 5) was also interesting. This finding is in line with the induction of ‘Wnt signaling pathway’ during adipogenesis suggested by the KEGG pathway analysis (Fig 4) and by data from another laboratory where it was observed an enhancement of adipogenesis by increase in calcium after the first 48 h of differentiation [63].

The analysis of the other GO categories, particularly the ‘GO Molecular function’, confirmed the stronger lipogenic phenotype during adipogenesis compared to osteogenesis (S3 File, sheet ‘GO Molecular function’). In addition, very few GO Biological processes were induced only during osteogenesis (S3 File and Fig 5). Among those it appears that the induction during osteogenesis and inhibition during adipogenesis of the “negative regulation of the extracellular matrix disassembly” might be important for allowing the deposition of collagen and other extracellular matrix components typical of bone formation and might be important for impeding formation of ECM during adipogenesis. Another interesting suggestion by the GO analysis is the induction of the “regulation of VEGF production” during osteogenesis (Fig 5). The porcine BMSC are able to produce and secrete a significant amount of VEGF [64]. Recently, it has been shown that production of VEGF by osteoprogenitors is important during bone healing [65] and it is well-known that vessel formation induced by VEGF is critical for bone healing [66, 67]. The data also suggested that among the most affected GO Biological processes there were several indicating a strong decrease of cell adhesion during adipogenesis but not during osteogenesis (Fig 5). None of the ‘GO Molecular function’ and ‘GO Cellular component’ were positively induced during osteogenesis and inhibited during adipogenesis (S3 File), with the exception of ‘Invadopodium membrane’ and ‘collagen type I’ among ‘GO Cellular component’ and ‘Collagen type V binding’ among ‘GO Molecular function’ category.

The analysis of the same dataset using DAVID, that uses an over-represented approach [68] and a more rich annotation database along with the GO categories, highlighted during adipogenesis in both MSC a consistent enrichment of functions related to cytoskeleton and its organization, among down-regulated genes, and lipid metabolism and response to hormones (particularly insulin) among up-regulated genes (S4 File). The osteogenic differentiation was featured by an enrichment of genes related to protein synthesis among down-regulated genes, particularly at 7 and 21 vs. 0dd, and an enrichment of genes related to glycoproteins and extracellular space components among up-regulated genes (S4 File).

Interestingly, none of the GO terms, that were considered highly impacted by the DIA, were also highly enriched by DAVID analysis. The discrepancy between the two methods is not surprising considering they are radical different approaches [18, 19]. The results from DAVID however appeared to be more similar to the results of the KEGG pathway analysis performed using DIA. For instance the reduction of protein synthesis during osteogenesis highlighted by DAVID analysis was also indicated by the inhibition of the ‘Ribosome’ pathway by the DIA (Fig 3). Similarly, the significant enrichment of the cytoskeleton among down-regulated genes during adipogenesis was also captured by the inhibition of ‘Regulation of actin cytoskeleton’ pathway (Fig 4).

A closer look at the results of the KEGG pathways by the enrichment analysis, performed by DAVID (S5 File), highlighted a concordance of results between DAVID and DIA, with most of the enriched pathways being also the higher impacted as calculated by the DIA (S2 File, Fig 3, and Fig 4). There was a strong agreement between the two approaches in indicating the ‘PPAR signaling pathway’ among the most important pathways during adipogenesis. However, some pathways, which were among the most impacted during adipogenesis, were not significantly enriched, such as ‘Renin-angiotensin system’. The ‘Basal cell carcinoma’, one of the pathways with the highest impact during adipogenesis as calculated by the DIA (Fig 4), was enriched only at 2 day adipogenesis in BMSC (S5 File). In addition, the ‘Wnt signaling pathway’ was not significantly enriched (simple EASE score >0.05) in any comparison, but tended to be significant (EASE score <0.10) in downregulated genes and only during adipogenesis (at 7 and 21 vs. 0dd in ASC and 7 vs. 0dd in BMSC, see S5 File). These data appear to support the DIA results and suggest that this pathway might be only relatively important during osteogenic differentiation in porcine MSC or it may suggest that the change in expression of components of this pathway is of low importance to determine the osteogenic fate of the porcine MSC, but can have a role in determining adipogenesis.

Even though we do not have an explanation for all the results, overall the combination of DIA and DAVID analyses uncovered a strong induction of functions related to lipid accumulation, increase of extracellular signaling, increase of sensitivity to angiogenesis, and strong reduction of cell-to-cell adhesion during adipogenesis but not during osteogenesis. Few functions were more induced in osteogenesis compared to adipogenesis, among those was the modification of extracellular space, particularly the accumulation of collagen, and likely a stronger production of VEGF.

### Transcription factors and other upstream regulators during adipogenesis and osteogenesis

**Fig 6:**
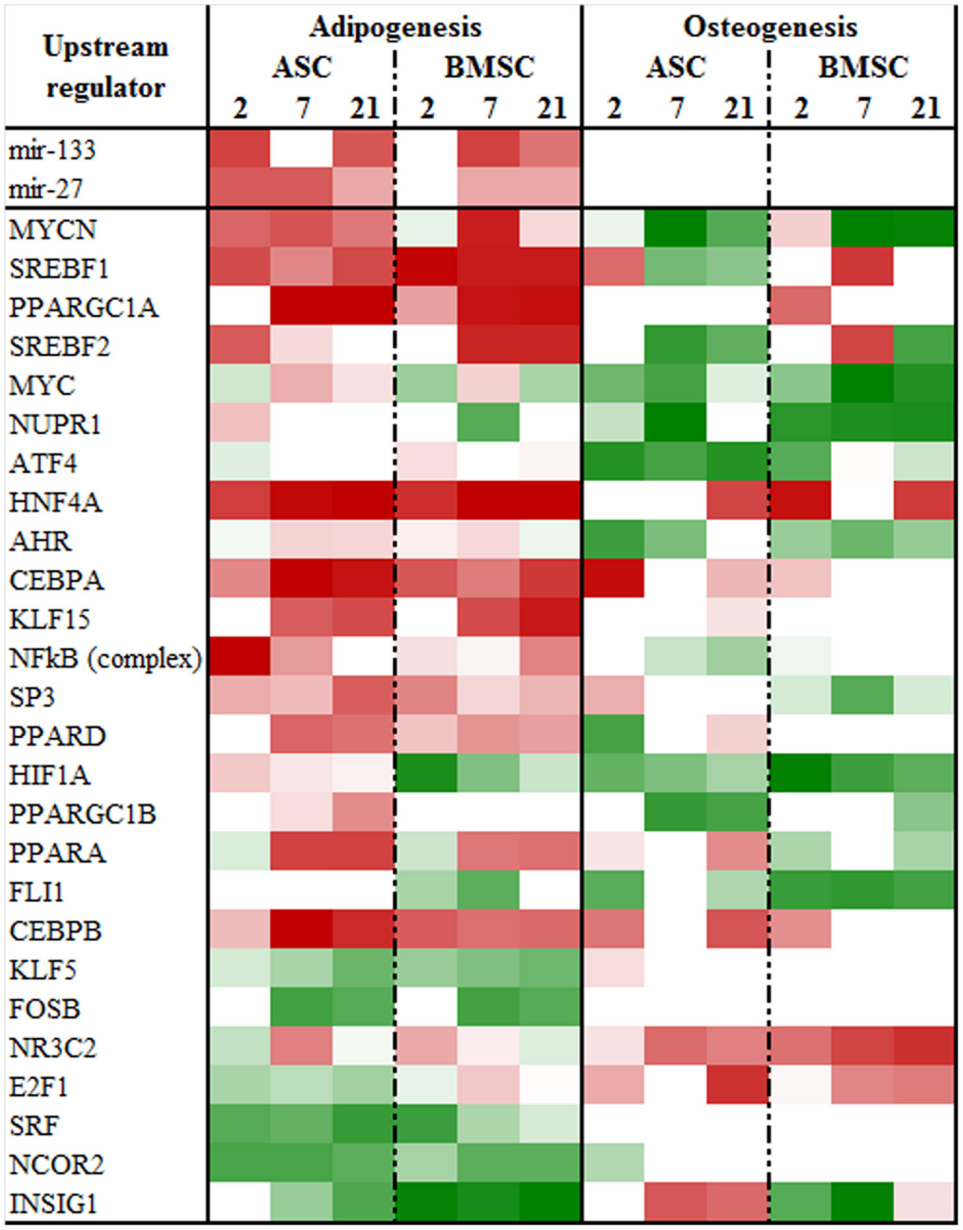
Relevant up-stream transcription regulators. Most activated or inhibited up-stream transcription regulators (transcription factors and ligand-activated nuclear receptors) as estimated by the z-score for each comparison from Ingenuity Pathway Analysis. Red denotes predicted activation and green predicted inhibition. The complete results, including other types of up-stream regulators are available in S6 File.

Complete results of the analysis of up-stream regulators among the DEG during differentiation are available in S6 File. In Fig 6 are reported the most important transcription factors (TF) and miRNA estimated by IPA to control the expression of DEG in adipogenic and osteogenic differentiation. The analysis confirmed a main role for the CEBPA and CEBPB in coordinating adipogenesis [69]. Surprisingly, the PPARγ was estimated to be activated during adipogenesis in ASC and BMSC and inhibited during osteogenesis in BMSC only (S6 File) but was not among the most activated or inhibited TF. Instead the other two PPAR isotypes, PPARα and PPARβ/δ and the co-activators PPARGC1A and PPARGC1B were estimated by IPA to be among the most activated during adipogenesis (Fig 6). The PPARGC1A is known to be involved in mitochondria proliferation and brown adipose tissue differentiation and PPARGC1B in adipogenesis [70], particularly in pre-adipocyte proliferation but not terminal adipogenesis [71] as also recently reviewed [72]. Those findings are however the opposite observed in our study, where the activation of PPARβ/δ was during terminal adipogenesis (Fig 6). The PPARα has been suggested in an early study to play a role in adipogenesis under certain conditions; however, it appears to play a more important role in brown adipose tissue and, likely, control oxidation of fatty acids in mature adipocytes [73].

A pivotal role of SREBP1 and SREBP2 activation during adipogenesis was also evidenced by IPA analysis (Fig 6). The SREBP1 plays a critical part in controlling transcription adaptation during adipogenesis [74]. Interestingly, the two SREBP isoforms were deemed to be inhibited by IPA during osteogenesis, particularly in ASC. The SREBP2 and, in minor fashion, SREBP1 are pivotal regulator of cholesterol synthesis [75]. The inhibition of those transcriptional factors supports the inhibition of cholesterol synthesis during osteogenesis observed in the present study (see above and Fig 3).

Other TF estimated to be significantly activated during adipogenesis were Hepatocyte Nuclear Factor 4 alpha (HNF4α), MYCN, and Kruppel-like factor 15 (KLF15; Fig 6). A direct role of HNF4α in adipogenesis has not been previously observed; however, this TF is very important to maintain homeostasis of triglycerides synthesis and cholesterol metabolism in liver [76]. This might be also true for porcine mesenchymal stem cells during adipogenesis. An early study observed an inhibitory effect of MYC on adipogenesis [77]. In our analysis MYC was partly inhibited during adipogenesis but its isoform, MYCN was significantly activated (Fig 6). The estimated activation of MYCN in our experiment has not apparent explanation. A role for KLF15 in adipogenesis has been previously demonstrated [78].

Few TF were inhibited during adipogenesis (Fig 6). Among the most inhibited transcription factors were some that have been previous known to inhibit adipogenesis, such as INSIG1 [79], NCOR1 [80], and FOSB [81], while the estimate inhibition of KLF5 was the opposite to its previously demonstrated pro-adipogenic role [82]. Two miRNA were deemed to be among the most induced during adipogenesis: miR27 and miR133. The miR27 has been previously associated with an anti-adipogenic effect in mouse as it targets PPARG (reviewed in [83]), while the miR133 has been associated to brown adipose differentiation [84]. Several more miRNA were uncovered by IPA to be overall induced or inhibited during differentiations (S6 File); however, when compared to previous miRNA observed to affect adipogenesis [83] we could not find any overlap. No miRNA were estimated to be more activated during osteogenesis compared to adipogenesis or being activated (i.e., z-score>2) during osteogenic differentiation (S6 File).

The osteogenesis was characterized by an overall inhibition of TF (Fig 6). Among those, estimated to be the most inhibited by IPA were MYC, NUPR1, HNF1A, and SREBP2. Contrary to our data, MYC was previously shown to be a positive regulator of osteogenic differentiation in human mesenchymal stem cells [85]. NUPR1 (Nuclear Protein 1) has not been previously associated with osteogenesis but it is a TF that responds to stress and increase survivability of cancerous cells [86]. Contrary to our data, the HNF1A (Hypoxia-Inducible Factor-1) has been demonstrated previously to be essential for the hypoxia-enhanced osteogenesis and its inhibition induces adipogenesis [87]. Among the TF only the NR3C2 (mineralocorticoid receptor) was estimated by IPA to be induced during osteogenesis. The NR3C2 has been previously reported to be involved in osteoblast differentiation [88] but very recently it has been demonstrated to have a negative effect on bone formation by the same nuclear receptor [89]. SREBP2 is a master regulator of cholesterol synthesis [90] and the inhibition of this TF supported the inhibition of sterol synthesis indicated by the DIA analysis (Fig 3).

Besides TF, several other upstream regulators were estimated to play a role in controlling adipogenesis and osteogenesis (S6 File). During adipogenesis among most activated upstream were insulin-like growth factor, insulin, and MAPK1. Their roles in adipogenesis have been very well established (reviewed in [91, 92]. Among the most inhibited upstream factors were TGFβ and interferon. The inhibitory role of TGFβ on adipogenesis is well established [93] (also reviewed in [94]). A role of interferon gamma, but not alpha, in inhibiting adipogenesis has been previously uncovered [95]. During osteogenesis was of relevance the inhibition of mTOR. An inhibition of mTOR pathways during osteogenesis was also revealed by the DIA analysis (Fig 4). mTOR seems to have a pro- and anti- osteogenic effect (reviewed in [96]). It has been demonstrated recently that mTOR is inhibited during early but activated during late osteogenesis [97].

Overall, the IPA analysis uncovered well-established upstream regulators for adipogenesis; however, some data (e.g., PPARGC1A, high enrichment of PPARα) seems also to indicate a differentiation toward brown adipose tissue. For what concern osteogenesis, very few upstream regulators were estimated to be induced. In addition, all the classical osteogenic transcription regulators (e.g., RUNX2, BMP4) were not estimated to be induced during osteogenesis (S6 File) and for the one we measured (BMP4) was not up-regulated (S1 File). Furthermore, several of the transcription factors estimated by IPA to be induced or inhibited during osteogenesis were the opposite of what was previously observed in mouse and human. This might indicate that the regulation of the porcine MSC osteogenesis might be specific for this species. This can have important implication for the use of pig as animal model for bone regeneration and, for this, warrants further investigation.

### *k*-mean cluster analysis

In order to uncover co-regulated genes and related pathways and functions we have performed k-mean cluster analysis using Genesis [98] in association with the enrichment analysis approach using DAVID [68]. The optimal number of clusters was determined by using the <1% gain of power of the Figure of Merit [98, 99]. The results indicated that 16 was the optimal number of *k*-mean clusters (S14 Fig).

**Fig 7:**
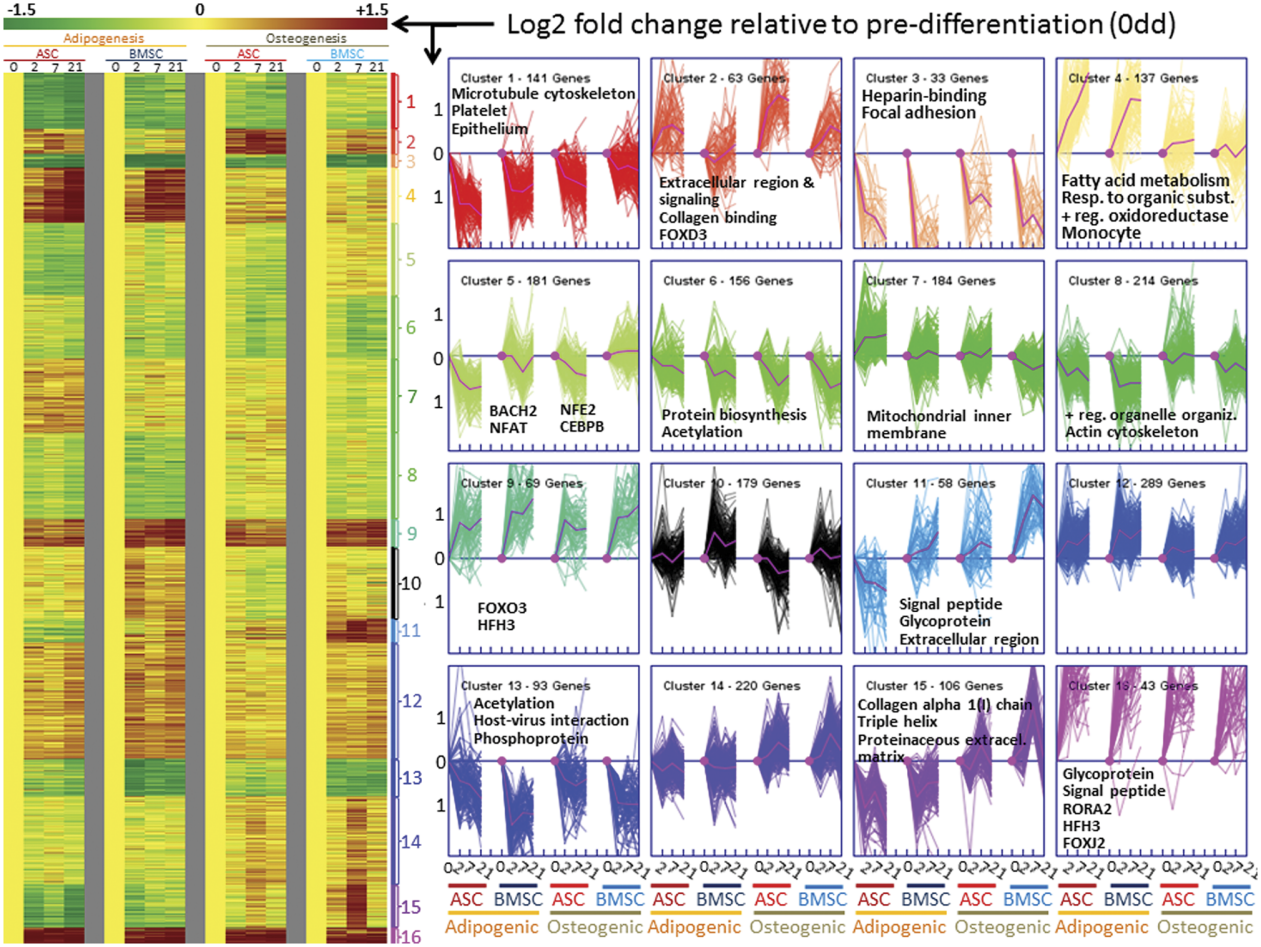
*k*-mean cluster analysis. The left panel reports the heat map and the right panel the expression graphs of the κ-mean clustering analysis using Genesis [98] among the 2,200 DEG due to differentiation × time × cell type. In the expression graphs are reported the number of genes and the most enriched functions (Benjamini-Hochberg FDR < 0.05) as determined by DAVID [68]. The ‘Functional Annotation Chart’ and the ‘Functional Annotation Clustering’ results that summarize the complete results of the enrichment analysis performed using DAVID of genes in clusters are available in S7 File and S8 File. Cluster 10, 12, and 14 had not biological terms enriched with a FDR<0.05. The purple line in each graph denotes the mean pattern. The Y-axis of the graphs denote the log2 fold change in each time point relative to pre-differentiation or 0dd and the numbers in X-axis denote the day of differentiation (0, 2, 7, and 21 day) in each cell type (ASC = adipose-derived stem cells; BMSC = bone marrow-derived stem cells) during adipogenic or osteogenic differentiation.

The pattern in expression for each cluster, both as heat map and as expression graphs, with their associated most enriched biological terms is reported in Fig 7. The associated cluster for each gene is reported in S1 File. The complete results of the functional enrichment analysis are reported in S7 File and S8 File. In order to evaluate the relationships between co-regulated genes we have performed a network analysis of genes in each cluster using Ingenuity Pathway Analysis (IPA) (S15 Fig and S9 File). With the purpose of identifying the transcription factors (TF) with a putative role in controlling the expression of genes in each cluster we have used IPA. The IPA allowed us to inquire about the TF with the largest number of target genes (S15 Fig) and the enrichment of overlap of TF with genes in each cluster (i.e., identification of the enrichment of putative upstream TFs, Table 1 and S10 File). Here we provide a summary of the main findings and a complete discussion is available in S11 File.

**Table 1:** Transcription factors controlling the expression of genes in clusters. Reported are the 31 transcription factors with a p-value of overlap <0.0005. The p-value of overlap indicates the statistical significance of genes in the dataset that are downstream of the transcription factor. Complete results are available in S10 File.

Cluster	TF^1^	Name	p-value^2^	Type^3^
2	FOXF1	Forkhead box protein F1	2.43E-04	TR
3	JUN	jun proto-oncogene	2.11E-07	TR
3	FOS	FBJ murine osteosarcoma viral oncogene homolog	2.11E-07	TR
3	TP53	tumor protein p53	1.12E-06	TR
3	ETS1	v-ets erythroblastosis virus E26 oncogene homolog 1 (avian)	2.51E-05	TR
3	WT1	Wilms tumor protein	8.26E-05	TR
3	SMAD7	SMAD family member 7	2.09E-04	TR
3	CDKN2A	cyclin-dependent kinase inhibitor 2A (inhibits CDK4)	3.07E-04	TR
4	PPARG	peroxisome proliferator-activate receptor gamma	6.14E-07	LDNR
4	PPARD	peroxisome proliferator-activate receptor delta	6.17E-06	LDNR
4	RELA	nuclear factor NF-kappa-B p65 subunit	6.16E-05	TR
4	PPARA	peroxisome proliferator-activate receptor alpha	1.39E-04	LDNR
4	SREBF1	Sterol regulatory element-binding transcription factor 1	1.53E-04	TR
4	NFkB	Nuclear factor NF-kappa-B (compex)	1.97E-04	TR
4	SMAD7	SMAD family member 7	1.93E-04	TR
4	NR3C1	glucocorticoid receptor	3.27E-04	TR
4	PRDM1	PR domain zinc finger protein 1 also known as BLIMP-1	4.41E-04	TR
4	HIF1A	Hypoxia-inducible factor 1-alpha	4.52E-04	TR
4	CEBPA*	CCAAT/enhancer binding protein (C/EBP), alpha	4.90E-04	TR
6	MYC	myelocytomatosis viral related oncogene, neuroblastoma derived (avian)	2.49E-04	TR
7	PPRC1	PPARG coactivator-related protein 1	1.50E-05	TR
8	CIITA	class II, major histocompatibility complex, transactivator	1.41E-04	TR
8	MYC	myelocytomatosis viral related oncogene, neuroblastoma derived (avian)	1.83E-04	TR
8	IRF4	interferon regulatory factor 4	2.28E-04	TR
8	FOS	FBJ murine osteosarcoma viral oncogene homolog	2.71E-04	TR
8	CTNNB1	catenin (cadherin-associated protein), beta 1	2.93E-04	TR
8	KLF5	Kruppel-like factor 5	4.80E-04	TR
11	IRF1	interferon regulatory factor 1	4.47E-04	TR
13	JUN	jun proto-oncogene	9.40E-05	TR
13	SP1	Sp1 transcription factor	4.10E-04	TR
16	FOXO3	forkhead box O3	2.28E-04	TR

^1^Transcription Factor

^2^The P-value denotes the significance of overlap with genes in the cluster (i.e., transcription factor expected to be activated or inhibited given the observed genes in the cluster) as calculated by Ingenuity Pathway Analysis.

^3^Type of molecule: TR = Transcription Regulator; LDNR = Ligand-dependent nuclear receptor

*Included as DEG in cluster 4.

The clusters 4, 7 and 12 grouped genes with an overall larger increase in expression during adipogenesis compared to osteogenesis. Among those, cluster 4 appears to be the cluster “signature” of the adipogenic differentiation due to its large difference between the two types of differentiation. The TF analysis of this cluster revealed an estimated large role of all 3 PPAR isotypes in controlling the expression of the genes belong to the cluster with PPARγ having the larger significance (Table 1). The cluster 4 had also the largest number of TF with the strongest significance of overlap. Most of those TF are related to lipid metabolism (e.g., SREBF1 and CEBPA) but also to inflammatory response (e.g., NFκB and RELA). The PPARγ and the CEBPα are known to play a pivotal role in adipogenesis [23, 100, 101]. Also, activation of PPARα has been reported to induce adipogenesis [22].

The clusters 11, 14, and 15 grouped genes that expression is increased during osteogenesis and reduced during adipogenesis. Among those only cluster 15 had an enrichment of collagen- and extracellular matrix-associated genes with a BH FDR<0.05 (Fig 7 and S7 File). Those genes can be considered expected during osteogenesis bearing in mind that collagen type I deposition in the extracellular matrix is essential for bone structure [102]. The cluster 15 appears to contain the “osteogenic signature genes”, based on the larger increase in expression pattern of those genes during osteogenesis compared to adipogenesis. As for the cluster 14, the cluster 11 is highly enriched by genes related to extracellular region, in particular signaling molecules (Fig 7 and S7 File), indicating a large co-regulation of cell-to-cell communication during osteogenesis.

Clusters 1, 3, 8 and 13 (and with lower magnitude also cluster 5) grouped genes with, on average, a consistent down-regulation during differentiations but with a larger decrease in adipogenic compared to osteogenic differentiation. Among others, those clusters were highly enriched with genes related to cytoskeleton organization (Fig 7 and S7 File). The cytoskeleton plays a pivotal role in cell shape, organelle organization, polarity, and sensing external forces that in turn are able to stimulate differentiation. This has been shown in BMSC [103] but also in ASC [104]. The coordinated down-regulation of the cytoskeleton during differentiation, particularly for the adipogenic differentiation, might be indicative of decreased cell interactions but might also indicate a decreased hypersensitivity of the cells to the stiffness of the surrounding environment. In human BMSC the phosphorylation of the actin cytoskeleton is an important phenomenon during *in vitro* osteogenesis [39]. Among the other clusters few were able to enrich biological terms.

Overall the cluster analysis in association with the TF network and TF overlapping analyses strongly indicated larger transcriptomics coordination and larger interactions of genes involved in adipogenic compared to osteogenic differentiation. In addition, the analysis highlighted a larger number of TF involved in driving adipogenesis compared to osteogenesis.

The enrichment analysis using IPA indicated that cluster 6 was highly enriched by genes involved in protein synthesis and was also highly enriched by target genes of MYCN or n-Myc (Table 1). The n-Myc is part of a family of transcription factors having similar functions, among those the v-Myc (or simply MYC) has been shown to play a crucial role in coordinating expression of ribosomal proteins that are involved in protein synthesis [105]. In our experiment, *MYC* was actually up-regulated during adipogenesis while *MYCN* was not affected by differentiations (S1 File). Although not clear due to the increase or not change in expression, our analysis suggest that MYC had likely played a role in coordinating the decrease in protein synthesis during osteogenic differentiations in our experiment with a probable more important role of n-Myc than v-Myc.

### Limitations

The current study presents several limitations. Some of those were previously pointed out [14], including the use of a microarray platform with less than half the transcripts present in the porcine genome, the incomplete annotation, and limitation of the DIA and of the enrichment analysis [19]. A further limitation of the used approaches is the analysis of pathways or other biological terms in isolation, i.e., without considering that pathways are highly interconnected and same genes can be involved in multiple pathways or biological terms [106].

Despite the high consistency between our data and several data produced *in vivo* underlined by the present study, the *in vitro* system is well known to poorly mimic the *in vivo* milieu. The possibility of running a similar analysis of differentiating cells *in vivo* is still a daunting challenge, but the advent of transgenic cells expressing fluorescent proteins (e.g., enhanced green fluorescent pig cells [107]) might be useful in order to track heterologous transplanted cells and their progeny during differentiations. This might be possible only in immune compromised animals considering that GFP cells can be eliminated by the host organism [108]. Those cells in different stages of differentiation can be isolated using fluorescent-activated or magnetic-activated sorting systems. Once isolated, the transcriptome can be analyzed using microarray or next generation sequencing.

## Concluding Remarks

**Fig 8:**
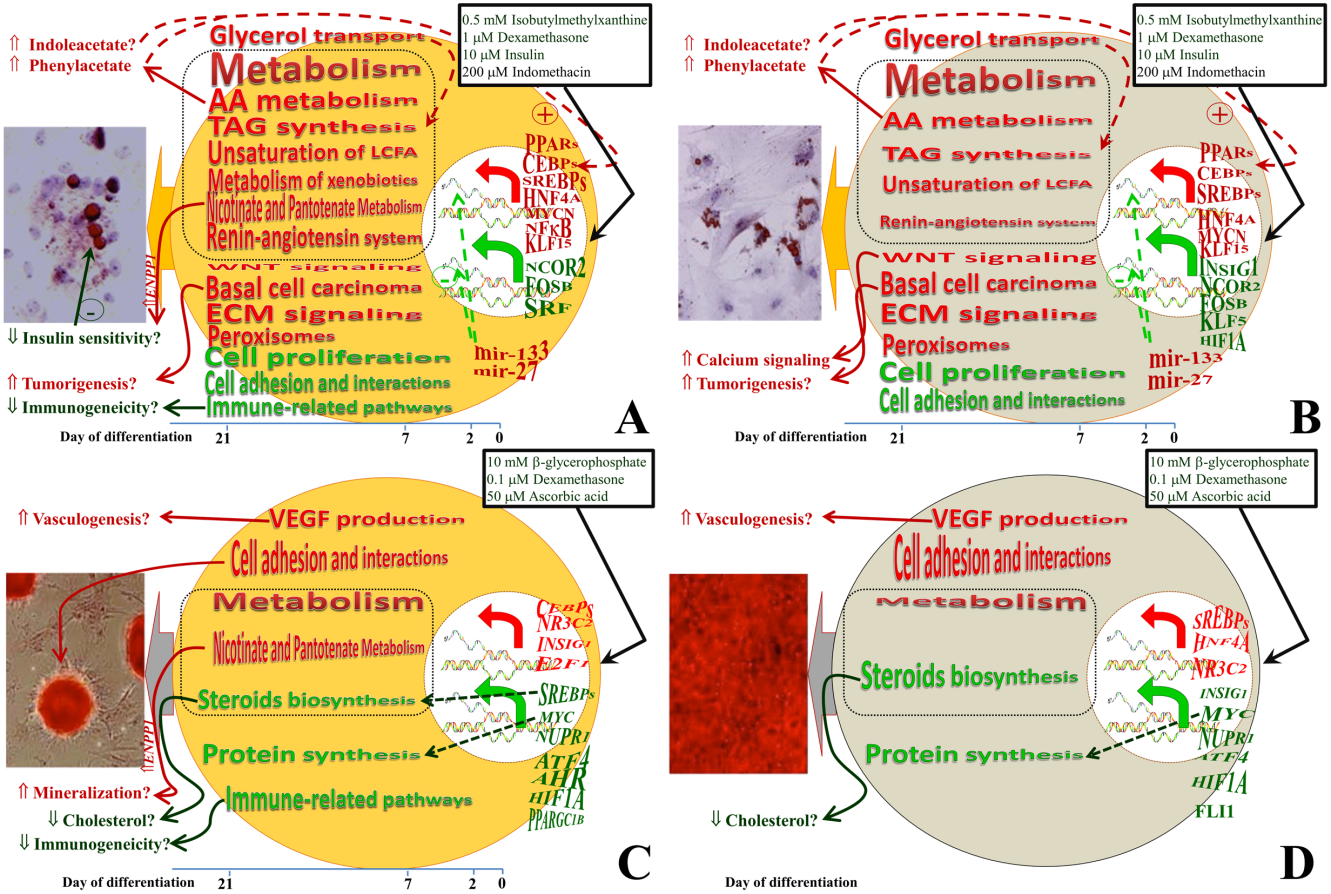
Overall functional adaptation of porcine ASC and BMSC during adipogenic and osteogenic differentiation. The model encompasses the most impacted and enriched terms plus transcription factors inferred by the analysis of transcriptomics changes in ASC and BMSC during adipogenic and osteogenic differentiation. The red font denotes induction while green font denotes inhibition. Shapes of the terms indicate an increase or decrease of the function from pre-differentiation (time 0) to 21 days of differentiation (from right to left). The analysis suggested that ASC during adipogenic differentiation (panel A) had a large transcriptomics change (**↰ (red arrow)** genes whose transcription was increased; **↰ (green arrow)** genes whose transcription was decreased; larger the size larger the number) with a more pronounce number of down-regulated compared to up-regulated genes. On the right, before the arrows, are reported the symbol of the most important transcription factors (TF) apparently regulating the genes affected by differentiations in each cell types. Red font TF are estimated to be activated and green are estimated to be inhibited. Shape of the TF denote change in activation (if larger in red from right to left → more activated during differentiation; if larger in green from right to left → more inhibited during differentiation; shape are derived from data reported in Fig 6). The functional analysis suggested an overall large induction of metabolism, encompassing triacylglycerol (TAG) synthesis with a fundamental role of unsaturation of long-chain fatty acids (LCFA) and import of glycerol. The amino acid (AA) metabolism, the metabolism of nicotinate and nicotinamide metabolism and pantothenate and CoA biosynthesis were induced with likely production of metabolites capable of direct or indirect positive effect on adipogenesis, partly through PPARγ. The ECM signaling, xenobiotics metabolism, and peroxisome were strongly induced. A potential increase in tumorigenesis by adipogenic differentiation can be deduced by the data. The cell proliferation and the cell-to-cell interactions were evidently inhibited by adipogenic differentiation. The transcriptomics data indicated also a large inhibition of immunogenicity as differentiation progressed in ASC. Functional analysis of transcriptomics changes by adipogenic differentiation in BMSC (panel B) suggested a very similar effect as for ASC. The osteogenic differentiation in ASC (panel C) and BMSC (panel D) was characterized by an increase in overall metabolism (larger in ASC vs. BMSC) but with an overall decrease of steroid biosynthesis and protein synthesis machinery. Data also indicated an overall increase in cell adhesion and accumulation of ECM with components such as collagen, and an increase in VEGF production with a likely consequent intensification of angiogenesis (if in an *in vivo* setting). Amid transcription factors the data suggested a pivotal role of PPAR isotypes and other transcription factors known to be involved in regulating lipid metabolism in controlling adipogenesis and novel TF in regulating osteogenesis. As for the adipogenic differentiation, also for the osteogenesis the data suggested that ASC were characterized by a decreased immunogenicity and an intensification of mineralization by the augmented expression of ectonucleotide pyrophosphatase/phosphodiesterase 1 (*ENPP1*) that plays a pivotal role in the nicotinate and nicotinamide metabolism and pantothenate and CoA biosynthesis.

The results from the present analysis allow proposing a dynamic model of adipogenic and osteogenic differentiation in porcine ASC and BMSC (Fig 8). Our data uncovered a larger and more coordinated transcriptomics adaptation during adipogenesis compared to osteogenesis with a similar magnitude between the two MSC. The larger number of DEG and the larger networks and TF involved in controlling the genes of the “adipose signature” cluster compared to any other cluster of genes, including the “osteogenic signature” one, support such conclusions. The DIA, together with the enrichment analysis, revealed a key role of PPAR signaling (likely PPARγ, but also PPARα can play a role) in determining the adipogenic fate of the cells. The role of the “Wnt signaling pathway” or other pathway was not large, with the former being more activated during adipogenesis, contrary to what previously reported.

The adipogenesis was featured by an increase in overall metabolism, particularly lipid and amino acid. The details analysis indicated an increased triacylglycerol synthesis and use of amino acids to produce adipogenic signaling molecules. The adipogenesis was also featured by decrease of cell-to-cell interactions, including focal adhesion and cytoskeletal regulation, and reduction of cell proliferation. The molecular basis for the adipogenic differentiation are relatively well-known [28]. The knowledge accumulated so far has been generated mostly from *in vitro* systems with large differences between culture conditions, type of cells used, and adipogenic inductions; however, it appears, also based on our data, that there is a relatively large agreement between the studies. Thus, we are expecting that such consistency should be found *in vivo* as well. Our analysis uncovered few relatively new molecular players (e.g., Wnt signaling more induced during adipogenesis, the role of amino acid metabolism during adipogenesis) but allowed for the first time to see the dynamic adaptation of the differentiation in large scale, permitting to propose an all-encompassing model (Fig 8).

Functional analysis of the osteogenic transcriptomics adaptation did not uncover any specific pathway being largely induced during osteogenesis. Relatively induced were pathways and functions related to cytoskeleton, cell-to-cell physical contact, extracellular matrix, and VEGF production. However, the data clearly indicated an overall inhibition of steroid synthesis at the end of bone formation and coordinated inhibition of protein synthesis machinery during osteogenesis, likely controlled by MYC. The reason for the reduction of steroids during osteogenesis, even though consistent with *in vivo* data, is not apparent and suggests the need for additional studies. The decrease of protein synthesis observed in the present experiment might be an important phenomenon during osteogenesis. The cluster analysis and network analyses indicated a moderately low coordination of genes affected by osteogenesis and low number of TF involved in such adaptation. Overall it appears that the osteogenesis is probably more regulated by other means, such as phosphorylation.

Even though not discussed in great detail, the functional analysis also uncovered some differences between the two MSC types. The differences were not so much related to a different transcriptomic perturbation during the differentiation, rather it appeared to be more related to a general adaptation to both differentiation types. This was particularly evident for ASC, prompting us to suggest that there is an “adipocyte memory” in ASC. The same could not be suggested for the BMSC. This can be a consequence of the MSC location. It has been established that the niche of the MSC is perivascular with relatively large vessels [9, 109, 110]. The adipose tissue is highly vascularized and the vessels are in very close physical proximity to the adipocytes, while the BMSC are more likely located in the middle of the bone marrow, far away from the endosteum, thus from the osteocytes [111]. Thus, the ASC are more closely associated with the adipocytes that might affect their niche.

Among others, an interesting feature that suggests additional research is the lower immunogenicity in differentiating ASC compared to BMSC revealed by our data. This, if further demonstrated, can provide additional reasons for using ASC instead of BMSC for clinical applications; however, the suggested larger tumorigenicity by adipogenesis, particularly in ASC, also needs to be carefully evaluated. The findings from the present work confirmed the high similarity in the transcriptomics response to adipogenic and osteogenic differentiation; thus, supporting an equivalence for use in tissue regeneration. Due to the easy and less painful harvesting of ASC compared to BMSC our data support the use of ASC as a better alternative than BMSC. However, the “adipose memory” deserves more in-depth investigations. The clinical consequence of such suggested “adipose memory” is at the present unknown.

## Prospective

The results from the present experiment allow proposing a dynamic model of *in vitro* osteogenesis and adipogenesis in porcine MSC. However, the limitations of the *in vitro* system might have hidden key information of MSC during the two differentiation types. Therefore, the use of heterologous transgenic MSC in combination with “omics” tools, such as RNA sequencing and epigenomics, can be of extreme value in order to study adipogenesis and osteogenesis *in vivo* so to improve the use of MSC for tissue repair.

## Materials and Methods

### Ethics statement

Subcutaneous back fat and bone marrow from femurs were harvested from three castrated Yorkshire crossbred male pigs under a protocol approved for this study by the University of Illinois Institutional Animal Care and Use Committee (IACUC #04296). The animals were euthanized via barbiturate overdose (Na pentobarbital, 90 mg/kg) followed by exsanguination. This is an acceptable method as described in the 2013 Report of the AVMA Panel on Euthanasia.

### ASC and BMSC isolation, culture, differentiation, and microarray analysis

The isolation, culture conditions, induction of differentiations, RNA extraction, and microarray analysis were previously described [14, 17]. Microarray data are deposited in the National Center for Biotechnology Information (NCBI) Gene Expression Omnibus (GEO) database (accession GSE25854).

### Statistical analysis

Microarray spots with median intensity ≥3 standard deviation above the median of the background and GenePix flag >100 were applied as filters to ensure high quality data. Data from a total of 82 microarrays were adjusted for dye and array effect (Loess normalization and array centering), duplicated spot intensities were not averaged and were subsequently used for statistical analysis. A mixed model with repeated measures was then fitted to the normalized log2-transformed adjusted ratios (sample/reference standard) using Proc MIXED (SAS, SAS Inst. Inc., Cary, NC). The model included the fixed effect of time (0, 2, 7, and 21 dd), cell type (ASC and BMSC), differentiation (osteogenic and adipogenic), interactions of time × cell type × differentiation. Pig (n = 3) was considered a random effect. P-values were adjusted for the number of genes tested using Benjamini and Hochberg’s false discovery rate (FDR) [112] to account for multiple comparisons. Differences in relative expression were considered significant at an FDR-adjusted P ≤0.05 for time × cell type × differentiation. Post-hoc P ≤0.05 was considered significant between pairwise comparisons. The difference in expression of genes is reported as fold change (2-fold = ±100% change).

### Dynamic Impact Approach (DIA) analysis

Recently, a novel Dynamic Impact Approach (DIA) method to analyze temporal transcriptomic data was developed [19]. The method uses the number of DEG, the magnitude, and the significance of changes in gene expression in order to provide an estimate of the dynamic impact of any treatment or condition on the system being studied. The DIA also provides a way to quickly interpret the results of the functional analysis.

Detailed description of the DIA has been previously reported [19]. The DIA analysis was performed for the Kyoto Encyclopedia of Genes and Genomes (KEGG) and the three gene ontology (GO) categories (i.e., Biological process, Molecular function, and Cellular component). For all the analysis the human database annotation was used (the pig microarray used in the present experiment was annotated using human annotations). For the KEGG the annotation was downloaded from the KEGG knowledge base (http://www.genome.jp/kegg/) in July 2011. For the GO all the database annotations were downloaded directly from the Gene Ontology Consortium website tool for annotation extraction (http://www.ebi.ac.uk/QuickGO/) in June 2012. For all databases analyzed we have used in DIA a cut-off of ≥30% genes present on our microarray platform vs. genome. In addition, for the GO analysis the terms with only 1 gene in our microarray were removed from the analysis. In order to capture the terms with the larger difference between differentiations or between cell types the direction of the impact (DoI) was used. For the differentiations the DoI in osteogenic was subtracted from the same differentiation in adipogenic. For the MSC type the DoI in BMSC was subtracted from the same differentiation in ASC.

In order to summarize the GO terms the REVIGO tool was used [60]. The GO ID were uploaded with the direction of the impact and the following options were used: ‘Allowed similarity’ = medium; the numbers associated to GO categories were “higher is better” for positive DoI and “lower is better” for negative DoI; database with GO term sizes = Homo sapiens; semantic similarity measure using SimRel. The table with results was downloaded and reformatted for Excel. The whole screen of the TreeMap was printed and copied in Adobe Photoshop Elements 9 with a DPI = 300 to produce the final figures.

### Up-stream transcription regulator analysis via Ingenuity Pathway Analysis (IPA)

To uncover the main up-stream regulators of the DEG we have taken advantage of the upstream regulator analysis in Ingenuity Pathway Analysis (IPA, Ingenuity® Systems, Mountain View, CA). The analysis uses an IPA Knowledge base to predict the expected causal effects between up-stream regulators and targets (i.e., DEG). The analysis provides the more plausible prediction of the status of the upstream regulator (i.e., activated or inhibited) by computing an overlap *p*-value and an activation z-score using the putative down-stream differentially expressed genes. For this purpose the whole dataset with Entrez-Gene IDs, statistical significance, and expression ratio of the entire experiment were uploaded into IPA.

### Cluster analyses

The *k*-means clustering analysis was conducted using Genesis software [98] with Euclidean distance. The decision on the number of clusters was based on the adjusted Figure of Merit (FOM) [99]. The analysis was conducted using the 2,200 DEG with the fold-change in expression log2 transformed with the following criteria: 50 FOM interactions, means centered, maximum of 50 clusters, and 100 interactions. The optimal number of clusters was selected when the comparison between two clusters resulted in ≤1% gain of power of prediction. The % gain of power of prediction of the FOM was considered as the % of the difference between FOM among consecutive clusters (S14 Fig) and calculated as [((FOM cluster_n_−FOM cluster_n+1_)/ FOM cluster_n_) × 100]. Based on the above criteria the cluster analysis with Genesis was run with 16 maximum clusters and 50 interactions.

### Functional enrichment analysis of DEG and genes in the clusters

The free available web tool Database for Annotation, Visualization and Integrated Discovery (DAVID) [113] was used for the enrichment analysis of the DEG in each comparison (separated by up-regulated and down-regulated gene lists) and genes in the clusters. The whole annotated microarray was used as background. The analysis was run using the default annotation dataset plus the ‘UCSC_TFBS’ in the Protein_Interactions annotation dataset and the UP_TISSUE in the Tissue_Expression annotation dataset. The ‘Functional Annotation Clustering’ and the ‘Functional Annotation Chart’ features were used to download all the results.

### Networks and transcription factor analysis of genes in clusters

The networks among genes in the same cluster were built using Ingenuity Pathway Analysis (IPA). A file containing a column for the Entrez Gene ID and a column for each cluster where the association of the Gene ID with the cluster was denoted by an arbitrary “P-value” = 0.01 was uploaded in IPA. The uploaded dataset was filtered based on the arbitrary “P-value” in order to retain for each cluster only the ID associated with the cluster. Using the filtered dataset for each cluster we built a new pathway incorporating all the genes in the cluster. By using the “Build-Path Explorer” option in IPA we identified all the direct and indirect interactions among genes in the cluster. Subsequently, using the “Path Designer” tool in IPA and using the “Build-Grow” option we added all the up-stream transcription factors for the genes in the cluster. The following options were selected: Interactions = Only “direct”; Grow out…”All the molecules”…that are “Upstream of the selected molecules”…and limit molecules to “Use Ingenuity Knowledge Base”; Relationship Types = “expression” and “transcription”; Molecule Types = “ligand-dependent nuclear receptor” and “transcription regulator”. All the other options were left as default. Once all the upstream molecules were added by IPA manually, we trimmed all the transcription factors that had less than 4 downstream molecules among the ones present in the cluster.

A Core Analysis of the cluster dataset was also run in order to obtain the Transcription Factor Analysis results. This analysis allowed identifying the transcription factors that may be responsible for gene expression changes observed in the experimental dataset.

## Supporting Information

S1 Fig Pattern of differentially expressed genes (DEG) in each differentiation and cell type. Overall view of the 2,200 transcripts significantly affected by cell type × time × differentiation with a False Discovery Rate ≤ 0.05. Images created using GeneSpring GX7. Adipo = adipogenic differentiation; Osteo = osteogenic differentiation; ASC = adipose-derived stem cells; BMSC = bone marrow-derived stem cells. The time (X-axis) is in day from beginning of differentiation. The Y-axis is log10 of fold-difference compared to day 0.
(TIFF)

S2 Fig Detailed depiction of the KEGG ‘Tryptophan metabolism’ at 7 day of adipogenic differentiation in ASC and BMSC. Shown is the response of the KEGG ‘Tryptophan metabolism’ in ASC and BMSC at 7 day of adipogenesis differentiation as obtained by the KegArray tool (http://www.kegg.jp/kegg/download/kegtools.html). Red-orange object denote up-regulation and green down-regulation relative to 0dd.
(TIF)

S3 Fig Detailed depiction of the KEGG ‘Phenylalanine metabolism’ at 7 day of adipogenic differentiation in ASC and BMSC. Shown is the difference in response of the KEGG ‘Phenylalanine metabolism’ in ASC and BMSC at 7 day of adipogenesis differentiation as obtained by the KegArray tool (http://www.kegg.jp/kegg/download/kegtools.html). Red-orange object denote up-regulation and green down-regulation relative to 0dd.
(TIF)

S4 Fig Detailed depiction of the KEGG ‘Metabolism of xenobiotics by cytochrome P450’ and ‘Drug metabolism—cytochrome P450’ at 21 day of adipogenic differentiation in ASC. Shown are the figures of the two pathways obtained by the KegArray tool (http://www.kegg.jp/kegg/download/kegtools.html). Red-orange object denote up-regulation and green down-regulation relative to 0dd.
(TIFF)

S5 Fig Detailed depiction of the KEGG ‘PPAR signaling pathway’ at 21 day of adipogenic differentiation in ASC and BMSC. Shown is the KEGG ‘PPAR signaling pathway’ at 21 days of adipogenic differentiation in ASC and BMSC as obtained by the KegArray tool (http://www.kegg.jp/kegg/download/kegtools.html). Striking is the similarity of the response between the two MSC. Red-orange object denote up-regulation and green down-regulation relative to 0dd.
(TIF)

S6 Fig Detailed depiction of the KEGG ‘Wnt signaling pathway’ at 7 day of adipogenic and osteogenic differentiation in BMSC. Shown is the response of the KEGG ‘Wnt signaling pathway’ at 7 day of adipogenic and osteogenic differentiation in BMSC as obtained by the KegArray tool (http://www.kegg.jp/kegg/download/kegtools.html). Red-orange object denote up-regulation and green down-regulation relative to 0dd.
(TIF)

S7 Fig Detailed depiction of the KEGG ‘Basal cell carcinoma’ at 21 day of adipogenic differentiation in ASC and BMSC. Shown is response of the KEGG ‘Basal cell carcinoma’ in ASC and BMSC at 21 day of adipogenic differentiation as obtained by the KegArray tool (http://www.kegg.jp/kegg/download/kegtools.html). Red-orange object denote up-regulation and green down-regulation relative to 0dd.
(TIF)

S8 Fig TreeMap view of GO Biological process terms with the larger difference in direction of the impact between adipogenic and osteogenic differentiation: terms more induced during adipogenesis. Results are from REVIGO analysis.
(TIF)

S9 Fig TreeMap view of GO Biological process terms with the larger difference in direction of the impact between adipogenic and osteogenic differentiation: terms more induced during osteogenesis. Results are from REVIGO analysis.
(TIF)

S10 Fig TreeMap view of GO Molecular process terms with the larger difference in direction of the impact between adipogenic and osteogenic differentiation: terms more induced during adipogenesis. Results are from REVIGO analysis.
(TIF)

S11 Fig TreeMap view of GO Molecular process terms with the larger difference in direction of the impact between adipogenic and osteogenic differentiation: terms more induced during osteogenesis. Results are from REVIGO analysis.
(TIF)

S12 Fig TreeMap view of GO Cellular component terms with the larger difference in direction of the impact between adipogenic and osteogenic differentiation: terms more induced during adipogenesis. Results are from REVIGO analysis.
(TIF)

S13 Fig TreeMap view of GO Cellular component terms with the larger difference in direction of the impact between adipogenic and osteogenic differentiation: terms more induced during osteogenesis. Results are from REVIGO analysis.
(TIF)

S14 Fig Figure of Merit and % Gain of Power for k-mean cluster. The figure of Merit (FOM) was calculated using Genesis [98]. The usual criterion for selecting the best number of clusters is the presence of the “elbow” of the FOM curve; however, it is very difficult to visualize the “elbow”. For this reason we have calculated the % Gain of Power as [(FOM previous cluster—FOM present cluster)/ FOM previous cluster × 100]. The % Gain of Power allows seeing the increase in power of prediction by adding an additional cluster. We deemed that the increase in power of prediction is worth to be considered if >1%; thus, we selected as the best number of cluster the first cluster which % Gain of Power is <1% (horizontal blue line denote 1% Gain of Power). In this case it was deemed 16 to be the best number of cluster (denoted by the blue arrow).
(TIF)

S15 Fig Network analysis of clusters plus putative transcription factors. In the left are shown the interactive networks among genes in each cluster constructed using Ingenuity Pathway Analysis (IPA). Details for each network are provided in S9 File. The graphs on the right denote: upper panel = the % of genes present in the network among all genes in the cluster eligible for network analysis in IPA; middle panel = the % of transcription factors (TF) present in the network among all genes in the cluster eligible for network analysis in IPA; bottom panel = the % of all TF with ≥3 down-stream genes (both present in the cluster and with a putative effect on transcription of genes included in the cluster) relative to all genes in the cluster eligible for network analysis in IPA.
(TIF)

S1 File Complete microarray dataset. Available are the annotation, the cluster number, the overall FDR (time x cell type x differentiation), the fold change, and the P-value between comparison for each gene.
(XLSX)

S2 File Complete KEGG pathway results from the Dynamic Impact Approach. The Excel file contains 3 sheets: ‘***KEGG pathways summary***’ containing the summary of impact and direction of the impact for the main categories and sub-categories of KEGG pathways; ‘***KEGG pathways***’ containing the impact and direction of the impact for each specific pathway in each category and sub-category of KEGG pathways; ‘***Sorted KEGG pathways***’ covering the specific pathways sorted in descending order by the sum of impact for each differentiation and in each cell type.
(XLSX)

S3 File Complete Gene Ontology results from the Dynamic Impact Approach. The Excel file contains 8 sheets including: ‘**legend**’, ‘***GO Biological process***’, ‘***GO Molecular function***’, and ‘***GO Cellular component***’ encompasses the impact and direction of the impact for the GO Biological process, GO Molecular function, and BO Cellular component, respectively, in descending order by the sum of the impact of all comparisons; ‘***Sorted GO***’ containing the sum of the impact of all time points comparison in adipogenic and osteogenic differentiation and in ASC and BMSC (plus the difference between adipogenic and osteogenic and ASC and BMSC) for each GO category; ‘***GO BP adipo vs*. *osteo***’, ‘***GO MF adipo vs*. *osteo***’, and ‘***GO CC adipo vs*. *osteo***’ include the results from REVIGO summary of the GO biological terms with associated differences in the direction of the impact between adipogenic and osteogenic differentiation.
(XLSX)

S4 File Complete Functional Annotation Clustering analysis results from DAVID for DEG in each time point vs. 0dd. The Excel file contains 13 sheets including a ‘legend’.
(XLSX)

S5 File Complete KEGG pathway analysis results from DAVID for DEG in each time point vs. 0dd. The Excel file contains 4 sheets encompassing the most enriched KEGG pathways as estimated by DAVID for each of the two differentiations in each mesenchymal stem cell.
(XLSX)

S6 File Complete results of the ups-stream regulators of DEG. The most relevant up-stream regulators of DEG in each MSC type during adipogenic and osteogenic differentiation were uncovered using Ingenuity Pathway Analysis. Red and green shade denote up-stream regulator deemed to be activated and inhibited, respectively.
(XLSX)

S7 File Complete Functional Annotation Chart from DAVID for each of the 16 *k*-mean cluster. The Excel file contains 16 sheets (one for each *k*-mean cluster) with the complete results from DAVID analysis with an EASE score ≤0.10. In yellow are highlighted the biological terms enriched with a Benjamini-Hochberg FDR ≤0.05.
(XLSX)

S8 File Complete Functional Annotation Clustering from DAVID for each of the 16 *k*-mean cluster. The Excel file contains 16 sheets (one for each *k*-mean cluster) with the results from the Functional Annotation Clustering of terms by DAVID analysis.
(XLSX)

S9 File Detailed figure of the networks for each of the 16 *k*-mean cluster built using Ingenuity Pathway Analysis. The PDF file contains the high quality image of the network among genes of each of the 16 cluster plus the transcription factor with ≥3 down-stream molecules. The genes belonging to the cluster are colored as the color of the cluster (see Fig 7). In the periphery of the network are reported the transcription factors (TF; with a larger font). The ones with the colored object are present in the cluster. The ones with white object are TF not present in the cluster but with ≥3 down-stream target among genes in the cluster as uncovered by Ingenuity Pathway Analysis.
(PDF)

S10 File Transcription factors with significant overlap with the genes in clusters. The Excel file contains one sheet with the results of the transcription factor analysis in each cluster performed using Ingenuity Pathway Analysis.
(XLSX)

S11 File Supplemental discussion of κ-mean cluster analysis. This is a supplemental discussion of clusters of grouped genes with overall changes in expression during adipogenic compared to osteogenic differentiation.
(DOCX)

S1 Video The clip is a time-lapse experiment of freshly isolated ASC induced to differentiated toward the osteogenic lineage for 3 days in 24 well plate (2 frames/s; pictures were taken every 10 min). The osteogenic medium was added at beginning of day 6 of culturing and cells were followed up to the end of day 8. The video also contains pictures of nodules stained with Alizarin Red S after 14 days of differentiation.
(MP4)

## References

[1] Kode JA, Mukherjee S, Joglekar MV, Hardikar AA. Mesenchymal stem cells: immunobiology and role in immunomodulation and tissue regeneration. Cytotherapy. 2009;11(4):377–91. Epub 2009/07/02. 912826134 [pii] 10.1080/14653240903080367 . 19568970

[2] Meirelles Lda S, Fontes AM, Covas DT, Caplan AI. Mechanisms involved in the therapeutic properties of mesenchymal stem cells. Cytokine Growth Factor Rev. 2009;20(5–6):419–27. Epub 2009/11/21. S1359-6101(09)00077-X [pii] 10.1016/j.cytogfr.2009.10.002 . 19926330

[3] Le Blanc K, Tammik C, Rosendahl K, Zetterberg E, Ringden O. HLA expression and immunologic properties of differentiated and undifferentiated mesenchymal stem cells. Exp Hematol. 2003;31(10):890–6. Epub 2003/10/11. S0301472X03001103 [pii]. . 14550804 10.1016/s0301-472x(03)00110-3

[4] Bartholomew A, Sturgeon C, Siatskas M, Ferrer K, McIntosh K, Patil S, et al Mesenchymal stem cells suppress lymphocyte proliferation in vitro and prolong skin graft survival in vivo. Exp Hematol. 2002;30(1):42–8. Epub 2002/02/02. S0301472X0100769X [pii]. . 11823036 10.1016/s0301-472x(01)00769-x

[5] Tse WT, Pendleton JD, Beyer WM, Egalka MC, Guinan EC. Suppression of allogeneic T-cell proliferation by human marrow stromal cells: implications in transplantation. Transplantation. 2003;75(3):389–97. Epub 2003/02/18. . 12589164 10.1097/01.TP.0000045055.63901.A9

[6] Slynarski K, Deszczynski J, Karpinski J. Fresh bone marrow and periosteum transplantation for cartilage defects of the knee. Transplant Proc. 2006;38(1):318–9. Epub 2006/03/01. S0041-1345(05)01553-8 [pii] 10.1016/j.transproceed.2005.12.075 . 16504736

[7] Veyrat-Masson R, Boiret-Dupre N, Rapatel C, Descamps S, Guillouard L, Guerin JJ, et al Mesenchymal content of fresh bone marrow: a proposed quality control method for cell therapy. Br J Haematol. 2007;139(2):312–20. Epub 2007/09/28. BJH6786 [pii] 10.1111/j.1365-2141.2007.06786.x . 17897309

[8] Friedenstein AJ, Chailakhjan RK, Lalykina KS. The development of fibroblast colonies in monolayer cultures of guinea-pig bone marrow and spleen cells. Cell Tissue Kinet. 1970;3(4):393–403. Epub 1970/10/01. . 5523063 10.1111/j.1365-2184.1970.tb00347.x

[9] Kolf CM, Cho E, Tuan RS. Mesenchymal stromal cells. Biology of adult mesenchymal stem cells: regulation of niche, self-renewal and differentiation. Arthritis Res Ther. 2007;9(1):204 Epub 2007/02/24. 10.1186/ar2116 17316462 PMC1860068

[10] Fraser JK, Wulur I, Alfonso Z, Hedrick MH. Fat tissue: an underappreciated source of stem cells for biotechnology. Trends Biotechnol. 2006;24(4):150–4. Epub 2006/02/21. S0167-7799(06)00028-X [pii] 10.1016/j.tibtech.2006.01.010 . 16488036

[11] Zuk PA, Zhu M, Mizuno H, Huang J, Futrell JW, Katz AJ, et al Multilineage cells from human adipose tissue: implications for cell-based therapies. Tissue Eng. 2001;7(2):211–28. Epub 2001/04/17. 10.1089/107632701300062859 . 11304456

[12] Zuk PA, Zhu M, Ashjian P, De Ugarte DA, Huang JI, Mizuno H, et al Human adipose tissue is a source of multipotent stem cells. Mol Biol Cell. 2002;13(12):4279–95. Epub 2002/12/12. 10.1091/mbc.E02-02-0105 . 12475952 PMC138633

[13] Monaco E, Bionaz M, Hollister SJ, Wheeler MB. Strategies for regeneration of the bone using porcine adult adipose-derived mesenchymal stem cells. Theriogenology. 2011;75(8):1381–99. Epub 2011/03/01. 10.1016/j.theriogenology.2010.11.020 . 21354606

[14] Monaco E, Bionaz M, Rodriguez-Zas S, Hurley WL, Wheeler MB. Transcriptomics Comparison between Porcine Adipose and Bone Marrow Mesenchymal Stem Cells during In Vitro Osteogenic and Adipogenic Differentiation. PLoS One. 2012;7(3):e32481 Epub 2012/03/14. 10.1371/journal.pone.0032481 22412878 PMC3296722

[15] Kim D, Monaco E, Maki A, de Lima AS, Kong HJ, Hurley WL, et al Morphologic and transcriptomic comparison of adipose- and bone-marrow-derived porcine stem cells cultured in alginate hydrogels. Cell Tissue Res. 2010;341(3):359–70. Epub 2010/08/04. 10.1007/s00441-010-1015-3 . 20680346

[16] Hattori H, Sato M, Masuoka K, Ishihara M, Kikuchi T, Matsui T, et al Osteogenic potential of human adipose tissue-derived stromal cells as an alternative stem cell source. Cells Tissues Organs. 2004;178(1):2–12. Epub 2004/11/20. CTO2004178001002 [pii] 10.1159/000081088 . 15550755

[17] Monaco E, Sobreira de Lima A, Bionaz M, Maki AJ, Wilson SW, Hurley WL, et al Morphological and Transcriptomic Comparison of Adipose and Bone Marrow Derived Porcine Stem Cells. The Open Tissue Engineering & Regenerative Medicine Journal. 2009;(2):20–33. 10.2174/1875043500902010020

[18] Huang da W, Sherman BT, Lempicki RA. Bioinformatics enrichment tools: paths toward the comprehensive functional analysis of large gene lists. Nucleic Acids Res. 2009;37(1):1–13. Epub 2008/11/27. gkn923 [pii] 10.1093/nar/gkn923 19033363 PMC2615629

[19] Bionaz M, Periasamy K, Rodriguez-Zas SL, Hurley WL, Loor JJ. A Novel Dynamic Impact Approach (DIA) for Functional Analysis of Time-Course Omics Studies: Validation Using the Bovine Mammary Transcriptome. PLoS One. 2012;7(3):e32455 Epub 2012/03/23. 10.1371/journal.pone.0032455 22438877 PMC3306320

[20] Ai-Aql ZS, Alagl AS, Graves DT, Gerstenfeld LC, Einhorn TA. Molecular mechanisms controlling bone formation during fracture healing and distraction osteogenesis. J Dent Res. 2008;87(2):107–18. Epub 2008/01/26. 18218835 10.1177/154405910808700215 PMC3109437

[21] Menssen A, Haupl T, Sittinger M, Delorme B, Charbord P, Ringe J. Differential gene expression profiling of human bone marrow-derived mesenchymal stem cells during adipogenic development. BMC genomics. 2011;12:461 Epub 2011/09/29. 10.1186/1471-2164-12-461 21943323 PMC3222637

[22] Spiegelman BM, Hu E, Kim JB, Brun R. PPAR gamma and the control of adipogenesis. Biochimie. 1997;79(2–3):111–2. Epub 1997/02/01. S0300-9084(97)81500-3 [pii]. . 9209705 10.1016/s0300-9084(97)81500-3

[23] Muruganandan S, Roman AA, Sinal CJ. Adipocyte differentiation of bone marrow-derived mesenchymal stem cells: cross talk with the osteoblastogenic program. Cellular and molecular life sciences: CMLS. 2009;66(2):236–53. Epub 2008/10/16. 10.1007/s00018-008-8429-z . 18854943 PMC11131547

[24] Takada I, Kouzmenko AP, Kato S. Wnt and PPARgamma signaling in osteoblastogenesis and adipogenesis. Nat Rev Rheumatol. 2009;5(8):442–7. Epub 2009/07/08. 10.1038/nrrheum.2009.137 . 19581903

[25] Akune T, Ohba S, Kamekura S, Yamaguchi M, Chung UI, Kubota N, et al PPAR gamma insufficiency enhances osteogenesis through osteoblast formation from bone marrow progenitors. J Clin Invest. 2004;113(6):846–55. 10.1172/Jci200419900 ISI:000220269200012. 15067317 PMC362117

[26] Fromm-Dornieden C, von der Heyde S, Lytovchenko O, Salinas-Riester G, Brenig B, Beissbarth T, et al Novel polysome messages and changes in translational activity appear after induction of adipogenesis in 3T3-L1 cells. BMC Mol Biol. 2012;13:9 10.1186/1471-2199-13-9 22436005 PMC3347988

[27] Spangenberg L, Shigunov P, Abud APR, Cofre AR, Stimamiglio MA, Kuligovski C, et al Polysome profiling shows extensive posttranscriptional regulation during human adipocyte stem cell differentiation into adipocytes. Stem Cell Res. 2013;11(2):902–12. 10.1016/j.scr.2013.06.002 WOS:000323586600019. 23845413

[28] Gregoire FM. Adipocyte differentiation: from fibroblast to endocrine cell. Exp Biol Med (Maywood). 2001;226(11):997–1002. Epub 2001/12/18. . 11743135 10.1177/153537020122601106

[29] Luckman SP, Hughes DE, Coxon FP, Graham R, Russell G, Rogers MJ. Nitrogen-containing bisphosphonates inhibit the mevalonate pathway and prevent post-translational prenylation of GTP-binding proteins, including Ras. J Bone Miner Res. 1998;13(4):581–9. Epub 1998/04/29. 10.1359/jbmr.1998.13.4.581 . 9556058

[30] Mundy G, Garrett R, Harris S, Chan J, Chen D, Rossini G, et al Stimulation of bone formation in vitro and in rodents by statins. Science. 1999;286(5446):1946–9. Epub 1999/12/03. . 10583956 10.1126/science.286.5446.1946

[31] Kumarasuriyar A, Lee I, Nurcombe V, Cool SM. De-sulfation of MG-63 cell glycosaminoglycans delays in vitro osteogenesis, up-regulates cholesterol synthesis and disrupts cell cycle and the actin cytoskeleton. Journal of cellular physiology. 2009;219(3):572–83. Epub 2009/01/15. 10.1002/jcp.21700 . 19142873

[32] Waku T, Shiraki T, Oyama T, Maebara K, Nakamori R, Morikawa K. The nuclear receptor PPARgamma individually responds to serotonin- and fatty acid-metabolites. The EMBO journal. 2010;29(19):3395–407. Epub 2010/08/19. 10.1038/emboj.2010.197 20717101 PMC2957204

[33] Lehmann JM, Lenhard JM, Oliver BB, Ringold GM, Kliewer SA. Peroxisome proliferator-activated receptors alpha and gamma are activated by indomethacin and other non-steroidal anti-inflammatory drugs. The Journal of biological chemistry. 1997;272(6):3406–10. Epub 1997/02/07. . 9013583 10.1074/jbc.272.6.3406

[34] Samid D, Shack S, Sherman LT. Phenylacetate: a novel nontoxic inducer of tumor cell differentiation. Cancer Res. 1992;52(7):1988–92. Epub 1992/04/01. . 1372534

[35] Harmey D, Hessle L, Narisawa S, Johnson KA, Terkeltaub R, Millan JL. Concerted regulation of inorganic pyrophosphate and osteopontin by akp2, enpp1, and ank: an integrated model of the pathogenesis of mineralization disorders. Am J Pathol. 2004;164(4):1199–209. Epub 2004/03/25. 10.1016/S0002-9440(10)63208-7 15039209 PMC1615351

[36] Pan W, Ciociola E, Saraf M, Tumurbaatar B, Tuvdendorj D, Prasad S, et al Metabolic consequences of ENPP1 overexpression in adipose tissue. Am J Physiol Endocrinol Metab. 2011;301(5):E901–11. Epub 2011/08/04. 10.1152/ajpendo.00087.2011 21810932 PMC3275110

[37] Ellero S, Chakhtoura G, Barreau C, Langouet S, Benelli C, Penicaud L, et al Xenobiotic-metabolizing cytochromes p450 in human white adipose tissue: expression and induction. Drug metabolism and disposition: the biological fate of chemicals. 2010;38(4):679–86. 10.1124/dmd.109.029249 . 20035023

[38] Kim DH, Puri N, Schwartzman ML. Cytochrome P450 Derived Epoxyeicosatrienoic Acid Retard Adipogenesis in Mesenchymal Stem cells by Heme Oxygenase-AKT Signaling. Faseb J. 2012;26. WOS:000310711305901.

[39] Lo T, Tsai CF, Shih YR, Wang YT, Lu SC, Sung TY, et al Phosphoproteomic analysis of human mesenchymal stromal cells during osteogenic differentiation. J Proteome Res. 2012;11(2):586–98. Epub 2011/11/18. 10.1021/pr200868p . 22088210

[40] Choi YA, Lim J, Kim KM, Acharya B, Cho JY, Bae YC, et al Secretome analysis of human BMSCs and identification of SMOC1 as an important ECM protein in osteoblast differentiation. J Proteome Res. 2010;9(6):2946–56. Epub 2010/04/03. 10.1021/pr901110q . 20359165

[41] Yamaguchi M. Nutritional factors and bone homeostasis: synergistic effect with zinc and genistein in osteogenesis. Mol Cell Biochem. 2012;366(1–2):201–21. Epub 2012/04/06. 10.1007/s11010-012-1298-7 . 22476903

[42] Goff SA. A unifying theory for general multigenic heterosis: energy efficiency, protein metabolism, and implications for molecular breeding. New Phytol. 2011;189(4):923–37. Epub 2010/12/21. 10.1111/j.1469-8137.2010.03574.x . 21166808

[43] Kapahi P. Protein synthesis and the antagonistic pleiotropy hypothesis of aging. Adv Exp Med Biol. 2010;694:30–7. Epub 2010/10/05. . 20886754 10.1007/978-1-4419-7002-2_3

[44] von der Heyde S, Fromm-Dornieden C, Salinas-Riester G, Beissbarth T, Baumgartner BG. Dynamics of mRNA and polysomal abundance in early 3T3-L1 adipogenesis. BMC Genomics. 2014;15:381 10.1186/1471-2164-15-381 24886538 PMC4039748

[45] Yin C, Xiao Y, Zhang W, Xu E, Liu W, Yi X, et al DNA microarray analysis of genes differentially expressed in adipocyte differentiation. Journal of biosciences. 2014;39(3):415–23. . 24845505 10.1007/s12038-014-9412-5

[46] Urs S, Smith C, Campbell B, Saxton AM, Taylor J, Zhang B, et al Gene expression profiling in human preadipocytes and adipocytes by microarray analysis. The Journal of nutrition. 2004;134(4):762–70. . 15051823 10.1093/jn/134.4.762

[47] Engeli S, Negrel R, Sharma AM. Physiology and pathophysiology of the adipose tissue renin-angiotensin system. Hypertension. 2000;35(6):1270–7. Epub 2000/06/17. . 10856276 10.1161/01.hyp.35.6.1270

[48] Nishizuka M, Koyanagi A, Osada S, Imagawa M. Wnt4 and Wnt5a promote adipocyte differentiation. FEBS Lett. 2008;582(21–22):3201–5. Epub 2008/08/19. 10.1016/j.febslet.2008.08.011 . 18708054

[49] Tamura M, Gu J, Danen EH, Takino T, Miyamoto S, Yamada KM. PTEN interactions with focal adhesion kinase and suppression of the extracellular matrix-dependent phosphatidylinositol 3-kinase/Akt cell survival pathway. The Journal of biological chemistry. 1999;274(29):20693–703. Epub 1999/07/10. . 10400703 10.1074/jbc.274.29.20693

[50] Dreyer C, Krey G, Keller H, Givel F, Helftenbein G, Wahli W. Control of the peroxisomal beta-oxidation pathway by a novel family of nuclear hormone receptors. Cell. 1992;68(5):879–87. Epub 1992/03/06. . 1312391 10.1016/0092-8674(92)90031-7

[51] Keller JM, Cable S, el Bouhtoury F, Heusser S, Scotto C, Armbruster L, et al Peroxisome through cell differentiation and neoplasia. Biol Cell. 1993;77(1):77–88. Epub 1993/01/01. . 8518747 10.1016/s0248-4900(05)80177-7

[52] De Miguel MP, Fuentes-Julian S, Blazquez-Martinez A, Pascual CY, Aller MA, Arias J, et al Immunosuppressive properties of mesenchymal stem cells: advances and applications. Curr Mol Med. 2012;12(5):574–91. Epub 2012/04/21. . 22515979 10.2174/156652412800619950

[53] Gotherstrom C, Ringden O, Tammik C, Zetterberg E, Westgren M, Le Blanc K. Immunologic properties of human fetal mesenchymal stem cells. Am J Obstet Gynecol. 2004;190(1):239–45. Epub 2004/01/30. 10.1016/j.ajog.2003.07.022 . 14749666

[54] Lindroos B, Suuronen R, Miettinen S. The potential of adipose stem cells in regenerative medicine. Stem Cell Rev. 2011;7(2):269–91. Epub 2010/09/21. 10.1007/s12015-010-9193-7 . 20853072

[55] Lin CS, Lin G, Lue TF. Allogeneic and Xenogeneic Transplantation of Adipose-Derived Stem Cells in Immunocompetent Recipients without Immunosuppressants. Stem cells and development. 2012 Epub 2012/05/25. 10.1089/scd.2012.0176 . 22621212 PMC3806387

[56] Yu JM, Jun ES, Bae YC, Jung JS. Mesenchymal stem cells derived from human adipose tissues favor tumor cell growth in vivo. Stem Cells Dev. 2008;17(3):463–73. Epub 2008/06/05. 10.1089/scd.2007.0181 . 18522494

[57] Yang C, Tibbitt MW, Basta L, Anseth KS. Mechanical memory and dosing influence stem cell fate. Nature materials. 2014;13(6):645–52. 10.1038/nmat3889 24633344 PMC4031270

[58] Narsinh KH, Plews J, Wu JC. Comparison of human induced pluripotent and embryonic stem cells: fraternal or identical twins? Molecular therapy: the journal of the American Society of Gene Therapy. 2011;19(4):635–8. 10.1038/mt.2011.41 21455209 PMC3070108

[59] Huang da W, Sherman BT, Zheng X, Yang J, Imamichi T, Stephens R, et al Extracting biological meaning from large gene lists with DAVID. Curr Protoc Bioinformatics. 2009;Chapter 13:Unit 13 1. Epub 2009/09/04. 10.1002/0471250953.bi1311s27 . 19728287

[60] Supek F, Bosnjak M, Skunca N, Smuc T. REVIGO summarizes and visualizes long lists of gene ontology terms. PLoS One. 2011;6(7):e21800 Epub 2011/07/27. 10.1371/journal.pone.0021800 21789182 PMC3138752

[61] Olswang Y, Blum B, Cassuto H, Cohen H, Biberman Y, Hanson RW, et al Glucocorticoids repress transcription of phosphoenolpyruvate carboxykinase (GTP) gene in adipocytes by inhibiting its C/EBP-mediated activation. The Journal of biological chemistry. 2003;278(15):12929–36. Epub 2003/02/01. 10.1074/jbc.M300263200 . 12560325

[62] Nye C, Kim J, Kalhan SC, Hanson RW. Reassessing triglyceride synthesis in adipose tissue. Trends Endocrinol Metab. 2008;19(10):356–61. Epub 2008/10/22. 10.1016/j.tem.2008.08.003 . 18929494

[63] Shi H, Halvorsen YD, Ellis PN, Wilkison WO, Zemel MB. Role of intracellular calcium in human adipocyte differentiation. Physiological genomics. 2000;3(2):75–82. Epub 2000/10/04. . 11015602 10.1152/physiolgenomics.2000.3.2.75

[64] Zisa D, Shabbir A, Suzuki G, Lee T. Vascular endothelial growth factor (VEGF) as a key therapeutic trophic factor in bone marrow mesenchymal stem cell-mediated cardiac repair. Biochem Biophys Res Commun. 2009;390(3):834–8. Epub 2009/10/20. 10.1016/j.bbrc.2009.10.058 19836359 PMC2788008

[65] Wang CJ, Huang KE, Sun YC, Yang YJ, Ko JY, Weng LH, et al VEGF modulates angiogenesis and osteogenesis in shockwave-promoted fracture healing in rabbits. J Surg Res. 2011;171(1):114–9. Epub 2010/05/11. 10.1016/j.jss.2010.01.045 . 20452608

[66] Portal-Nunez S, Lozano D, Esbrit P. Role of angiogenesis on bone formation. Histol Histopathol. 2012;27(5):559–66. . 22419020 10.14670/HH-27.559

[67] Keramaris NC, Calori GM, Nikolaou VS, Schemitsch EH, Giannoudis PV. Fracture vascularity and bone healing: a systematic review of the role of VEGF. Injury. 2008;39 Suppl 2:S45–57. 10.1016/S0020-1383(08)70015-9 . 18804573

[68] Huang da W, Sherman BT, Tan Q, Kir J, Liu D, Bryant D, et al DAVID Bioinformatics Resources: expanded annotation database and novel algorithms to better extract biology from large gene lists. Nucleic Acids Res. 2007;35(Web Server issue):W169–75. Epub 2007/06/20. gkm415 [pii] 10.1093/nar/gkm415 17576678 PMC1933169

[69] Cristancho AG, Lazar MA. Forming functional fat: a growing understanding of adipocyte differentiation. Nature reviews Molecular cell biology. 2011;12(11):722–34. Epub 2011/09/29. 10.1038/nrm3198 . 21952300 PMC7171550

[70] Spiegelman BM, Puigserver P, Wu Z. Regulation of adipogenesis and energy balance by PPARgamma and PGC-1. Int J Obes Relat Metab Disord. 2000;24 Suppl 4:S8–10. Epub 2000/12/29. . 11126248 10.1038/sj.ijo.0801492

[71] Hansen JB, Zhang H, Rasmussen TH, Petersen RK, Flindt EN, Kristiansen K. Peroxisome proliferator-activated receptor delta (PPARdelta) -mediated regulation of preadipocyte proliferation and gene expression is dependent on cAMP signaling. The Journal of biological chemistry. 2001;276(5):3175–82. Epub 2000/11/09. 10.1074/jbc.M005567200 . 11069900

[72] Costa V, Gallo MA, Letizia F, Aprile M, Casamassimi A, Ciccodicola A. PPARG: Gene Expression Regulation and Next-Generation Sequencing for Unsolved Issues. PPAR Res. 2010;2010 Epub 2010/09/28. 10.1155/2010/409168 20871817 PMC2943117

[73] Christodoulides C, Vidal-Puig A. PPARs and adipocyte function. Molecular and cellular endocrinology. 2010;318(1–2):61–8. Epub 2009/09/24. 10.1016/j.mce.2009.09.014 . 19772894

[74] Ayala-Sumuano JT, Velez-Delvalle C, Beltran-Langarica A, Marsch-Moreno M, Cerbon-Solorzano J, Kuri-Harcuch W. Srebf1a is a key regulator of transcriptional control for adipogenesis. Sci Rep. 2011;1:178 Epub 2012/02/23. 10.1038/srep00178 22355693 PMC3240949

[75] Weber LW, Boll M, Stampfl A. Maintaining cholesterol homeostasis: sterol regulatory element-binding proteins. World J Gastroenterol. 2004;10(21):3081–7. Epub 2004/10/01. . 15457548 10.3748/wjg.v10.i21.3081 PMC4611246

[76] Yin L, Ma H, Ge X, Edwards PA, Zhang Y. Hepatic hepatocyte nuclear factor 4alpha is essential for maintaining triglyceride and cholesterol homeostasis. Arterioscler Thromb Vasc Biol. 2011;31(2):328–36. Epub 2010/11/13. 10.1161/ATVBAHA.110.217828 21071704 PMC3079249

[77] Freytag SO, Geddes TJ. Reciprocal regulation of adipogenesis by Myc and C/EBP alpha. Science. 1992;256(5055):379–82. Epub 1992/04/17. . 1566086 10.1126/science.256.5055.379

[78] Mori T, Sakaue H, Iguchi H, Gomi H, Okada Y, Takashima Y, et al Role of Kruppel-like factor 15 (KLF15) in transcriptional regulation of adipogenesis. The Journal of biological chemistry. 2005;280(13):12867–75. Epub 2005/01/25. 10.1074/jbc.M410515200 . 15664998

[79] Li J, Takaishi K, Cook W, McCorkle SK, Unger RH. Insig-1 "brakes" lipogenesis in adipocytes and inhibits differentiation of preadipocytes. Proceedings of the National Academy of Sciences of the United States of America. 2003;100(16):9476–81. Epub 2003/07/19. 10.1073/pnas.1133426100 12869692 PMC170943

[80] Yu C, Markan K, Temple KA, Deplewski D, Brady MJ, Cohen RN. The nuclear receptor corepressors NCoR and SMRT decrease peroxisome proliferator-activated receptor gamma transcriptional activity and repress 3T3-L1 adipogenesis. The Journal of biological chemistry. 2005;280(14):13600–5. Epub 2005/02/05. 10.1074/jbc.M409468200 . 15691842

[81] Sabatakos G, Sims NA, Chen J, Aoki K, Kelz MB, Amling M, et al Overexpression of DeltaFosB transcription factor(s) increases bone formation and inhibits adipogenesis. Nat Med. 2000;6(9):985–90. Epub 2000/09/06. 10.1038/79683 . 10973317

[82] Oishi Y, Manabe I, Tobe K, Tsushima K, Shindo T, Fujiu K, et al Kruppel-like transcription factor KLF5 is a key regulator of adipocyte differentiation. Cell Metab. 2005;1(1):27–39. Epub 2005/08/02. 10.1016/j.cmet.2004.11.005 . 16054042

[83] McGregor RA, Choi MS. microRNAs in the regulation of adipogenesis and obesity. Curr Mol Med. 2011;11(4):304–16. Epub 2011/04/22. 21506921 10.2174/156652411795677990 PMC3267163

[84] Trajkovski M, Ahmed K, Esau CC, Stoffel M. MyomiR-133 regulates brown fat differentiation through Prdm16. Nat Cell Biol. 2012;14(12):1330–5. Epub 2012/11/13. 10.1038/ncb2612 . 23143398

[85] Piek E, Sleumer LS, van Someren EP, Heuver L, de Haan JR, de Grijs I, et al Osteo-transcriptomics of human mesenchymal stem cells: accelerated gene expression and osteoblast differentiation induced by vitamin D reveals c-MYC as an enhancer of BMP2-induced osteogenesis. Bone. 2010;46(3):613–27. Epub 2009/10/28. 10.1016/j.bone.2009.10.024 . 19857615

[86] Cano CE, Hamidi T, Sandi MJ, Iovanna JL. Nupr1: the Swiss-knife of cancer. Journal of cellular physiology. 2011;226(6):1439–43. Epub 2010/07/27. 10.1002/jcp.22324 . 20658514

[87] Wagegg M, Gaber T, Lohanatha FL, Hahne M, Strehl C, Fangradt M, et al Hypoxia promotes osteogenesis but suppresses adipogenesis of human mesenchymal stromal cells in a hypoxia-inducible factor-1 dependent manner. PLoS One. 2012;7(9):e46483 Epub 2012/10/03. 10.1371/journal.pone.0046483 23029528 PMC3459928

[88] Jaffe IZ, Tintut Y, Newfell BG, Demer LL, Mendelsohn ME. Mineralocorticoid receptor activation promotes vascular cell calcification. Arterioscler Thromb Vasc Biol. 2007;27(4):799–805. Epub 2007/01/20. 10.1161/01.ATV.0000258414.59393.89 . 17234727

[89] Fumoto T, Ishii KA, Ito M, Berger S, Schutz G, Ikeda K. Mineralocorticoid receptor function in bone metabolism and its role in glucocorticoid-induced osteopenia. Biochem Biophys Res Commun. 2014;447(3):407–12. Epub 2014/04/10. 10.1016/j.bbrc.2014.03.149 . 24713303

[90] Eberle D, Hegarty B, Bossard P, Ferre P, Foufelle F. SREBP transcription factors: master regulators of lipid homeostasis. Biochimie. 2004;86(11):839–48. Epub 2004/12/14. 10.1016/j.biochi.2004.09.018 . 15589694

[91] James AW. Review of Signaling Pathways Governing MSC Osteogenic and Adipogenic Differentiation. Scientifica (Cairo). 2013;2013:684736 Epub 2014/01/15. 10.1155/2013/684736 24416618 PMC3874981

[92] Aubert J, Belmonte N, Dani C. Role of pathways for signal transducers and activators of transcription, and mitogen-activated protein kinase in adipocyte differentiation. Cellular and molecular life sciences: CMLS. 1999;56(5–6):538–42. Epub 2001/02/24. . 11212303 10.1007/s000180050450 PMC11147030

[93] Park JG, Lee DH, Moon YS, Kim KH. Reversine increases the plasticity of lineage-committed preadipocytes to osteogenesis by inhibiting adipogenesis through induction of TGF-beta pathway in vitro. Biochem Biophys Res Commun. 2014;446(1):30–6. Epub 2014/02/20. 10.1016/j.bbrc.2014.02.036 . 24548409

[94] Ali AT, Hochfeld WE, Myburgh R, Pepper MS. Adipocyte and adipogenesis. Eur J Cell Biol. 2013;92(6–7):229–36. Epub 2013/07/24. 10.1016/j.ejcb.2013.06.001 . 23876739

[95] Vidal C, Bermeo S, Li W, Huang D, Kremer R, Duque G. Interferon gamma inhibits adipogenesis in vitro and prevents marrow fat infiltration in oophorectomized mice. Stem cells. 2012;30(5):1042–8. Epub 2012/02/15. 10.1002/stem.1063 . 22331815

[96] Xiang X, Zhao J, Xu G, Li Y, Zhang W. mTOR and the differentiation of mesenchymal stem cells. Acta Biochim Biophys Sin (Shanghai). 2011;43(7):501–10. Epub 2011/06/07. 10.1093/abbs/gmr041 . 21642276

[97] Pantovic A, Krstic A, Janjetovic K, Kocic J, Harhaji-Trajkovic L, Bugarski D, et al Coordinated time-dependent modulation of AMPK/Akt/mTOR signaling and autophagy controls osteogenic differentiation of human mesenchymal stem cells. Bone. 2013;52(1):524–31. Epub 2012/11/01. 10.1016/j.bone.2012.10.024 . 23111315

[98] Sturn A, Quackenbush J, Trajanoski Z. Genesis: cluster analysis of microarray data. Bioinformatics. 2002;18(1):207–8. Epub 2002/02/12. . 11836235 10.1093/bioinformatics/18.1.207

[99] Yeung KY, Haynor DR, Ruzzo WL. Validating clustering for gene expression data. Bioinformatics. 2001;17(4):309–18. ISI:000168325600003. 11301299 10.1093/bioinformatics/17.4.309

[100] Grontved L, Madsen MS, Boergesen M, Roeder RG, Mandrup S. MED14 tethers mediator to the N-terminal domain of peroxisome proliferator-activated receptor gamma and is required for full transcriptional activity and adipogenesis. Mol Cell Biol. 2010;30(9):2155–69. Epub 2010/03/03. 10.1128/MCB.01238-09 20194623 PMC2863581

[101] Rangwala SM, Lazar MA. Transcriptional control of adipogenesis. Annu Rev Nutr. 2000;20:535–59. Epub 2000/08/15. 10.1146/annurev.nutr.20.1.535 . 10940345

[102] Tzaphlidou M. The role of collagen in bone structure: an image processing approach. Micron. 2005;36(7–8):593–601. Epub 2005/10/08. 10.1016/j.micron.2005.05.009 . 16209926

[103] Engler AJ, Sen S, Sweeney HL, Discher DE. Matrix elasticity directs stem cell lineage specification. Cell. 2006;126(4):677–89. Epub 2006/08/23. S0092-8674(06)00961-5 [pii] 10.1016/j.cell.2006.06.044 . 16923388

[104] Choi YS, Vincent LG, Lee AR, Dobke MK, Engler AJ. Mechanical derivation of functional myotubes from adipose-derived stem cells. Biomaterials. 2012;33(8):2482–91. Epub 2011/12/27. S0142-9612(11)01459-1 [pii] 10.1016/j.biomaterials.2011.12.004 22197570 PMC3261363

[105] van Riggelen J, Yetil A, Felsher DW. MYC as a regulator of ribosome biogenesis and protein synthesis. Nat Rev Cancer. 2010;10(4):301–9. Epub 2010/03/25. 10.1038/nrc2819 . 20332779

[106] Khatri P, Sirota M, Butte AJ. Ten Years of Pathway Analysis: Current Approaches and Outstanding Challenges. Plos Computational Biology. 2012;7(3). ARTN e32455 10.1371/journal.pone.0032455 ISI:000303309100009. PMC3285573 22383865

[107] Lai L, Park KW, Cheong HT, Kuhholzer B, Samuel M, Bonk A, et al Transgenic pig expressing the enhanced green fluorescent protein produced by nuclear transfer using colchicine-treated fibroblasts as donor cells. Mol Reprod Dev. 2002;62(3):300–6. Epub 2002/07/12. 10.1002/mrd.10146 . 12112592

[108] Stripecke R, Carmen Villacres M, Skelton D, Satake N, Halene S, Kohn D. Immune response to green fluorescent protein: implications for gene therapy. Gene Ther. 1999;6(7):1305–12. Epub 1999/08/24. . 10455440 10.1038/sj.gt.3300951

[109] Kuhn NZ, Tuan RS. Regulation of stemness and stem cell niche of mesenchymal stem cells: implications in tumorigenesis and metastasis. Journal of cellular physiology. 2010;222(2):268–77. Epub 2009/10/23. 10.1002/jcp.21940 . 19847802

[110] Lin CS, Xin ZC, Deng CH, Ning H, Lin G, Lue TF. Defining adipose tissue-derived stem cells in tissue and in culture. Histol Histopathol. 2010;25(6):807–15. Epub 2010/04/09. . 20376787 10.14670/HH-25.807

[111] Oh IH, Kwon KR. Concise review: multiple niches for hematopoietic stem cell regulations. Stem cells. 2010;28(7):1243–9. Epub 2010/06/03. 10.1002/stem.453 . 20517982

[112] Benjamini Y, Hochberg Y. Controlling the False Discovery Rate—a Practical and Powerful Approach to Multiple Testing. J Roy Stat Soc B Met. 1995;57(1):289–300. ISI:A1995QE45300017.

[113] Huang da W, Sherman BT, Lempicki RA. Systematic and integrative analysis of large gene lists using DAVID bioinformatics resources. Nat Protoc. 2009;4(1):44–57. Epub 2009/01/10. nprot.2008.211 [pii] 10.1038/nprot.2008.211 . 19131956

