# Supplementary figures and images for: Transcription Adaptation during In Vitro Adipogenesis and Osteogenesis of Porcine Mesenchymal Stem Cells: Dynamics of Pathways, Biological Processes, Up-Stream Regulators, and Gene Networks
Source: PLoS One. 2015 Sep 23;10(9):e0137644. doi: 10.1371/journal.pone.0137644 (PMC4580618; doi:10.1371/journal.pone.0137644)

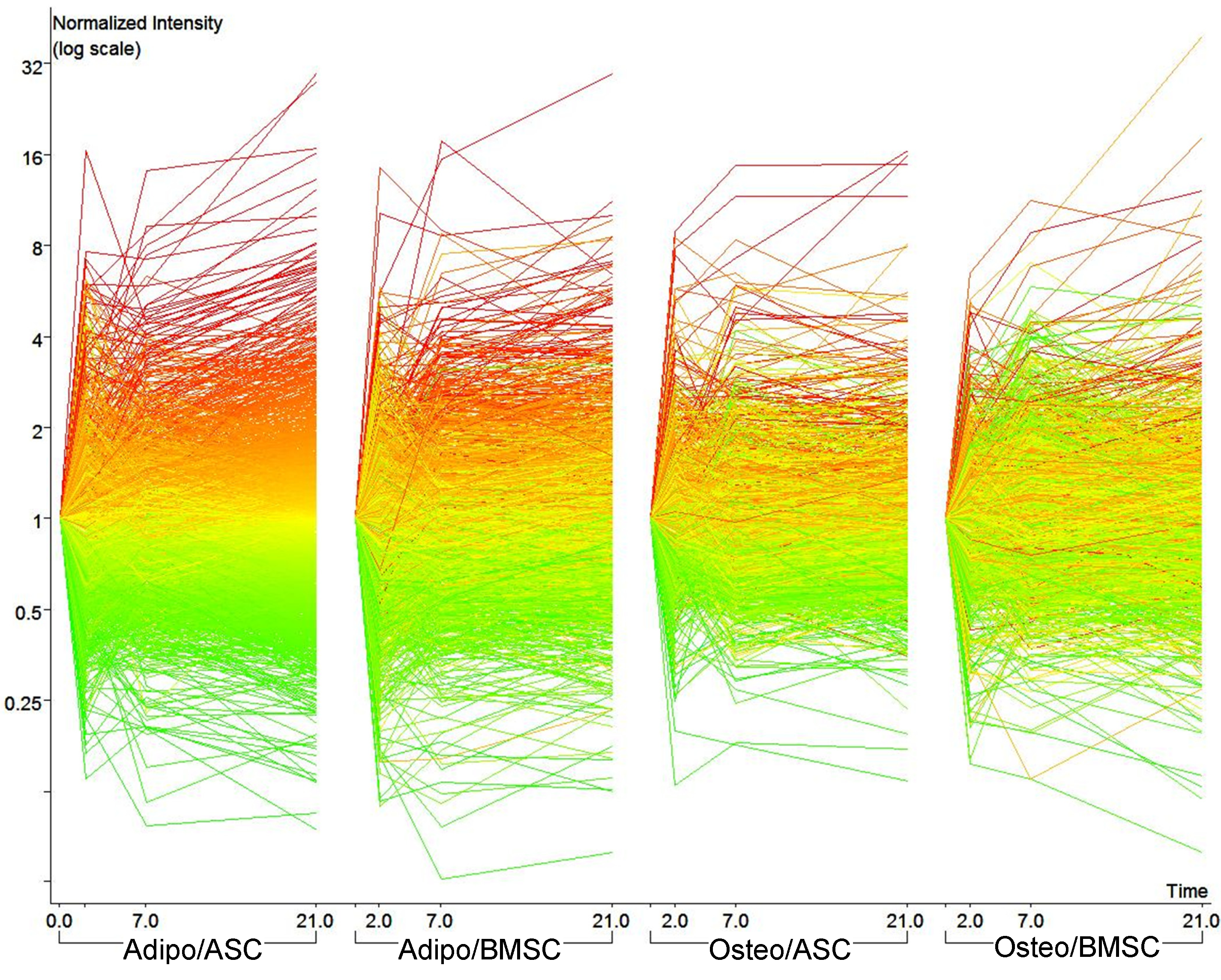

Supplement: S1 Fig — Overall view of the 2,200 transcripts significantly affected by cell type × time × differentiation with a False Discovery Rate ≤ 0.05. Images created using GeneSpring GX7. Adipo = adipogenic differentiation; Osteo = osteogenic differentiation; ASC = adipose-derived stem cells; BMSC = bone marrow-derived stem cells. The time (X-axis) is in day from beginning of differentiation. The Y-axis is log10 of fold-difference compared to day 0. (TIFF) [file pone.0137644.s001.tiff]

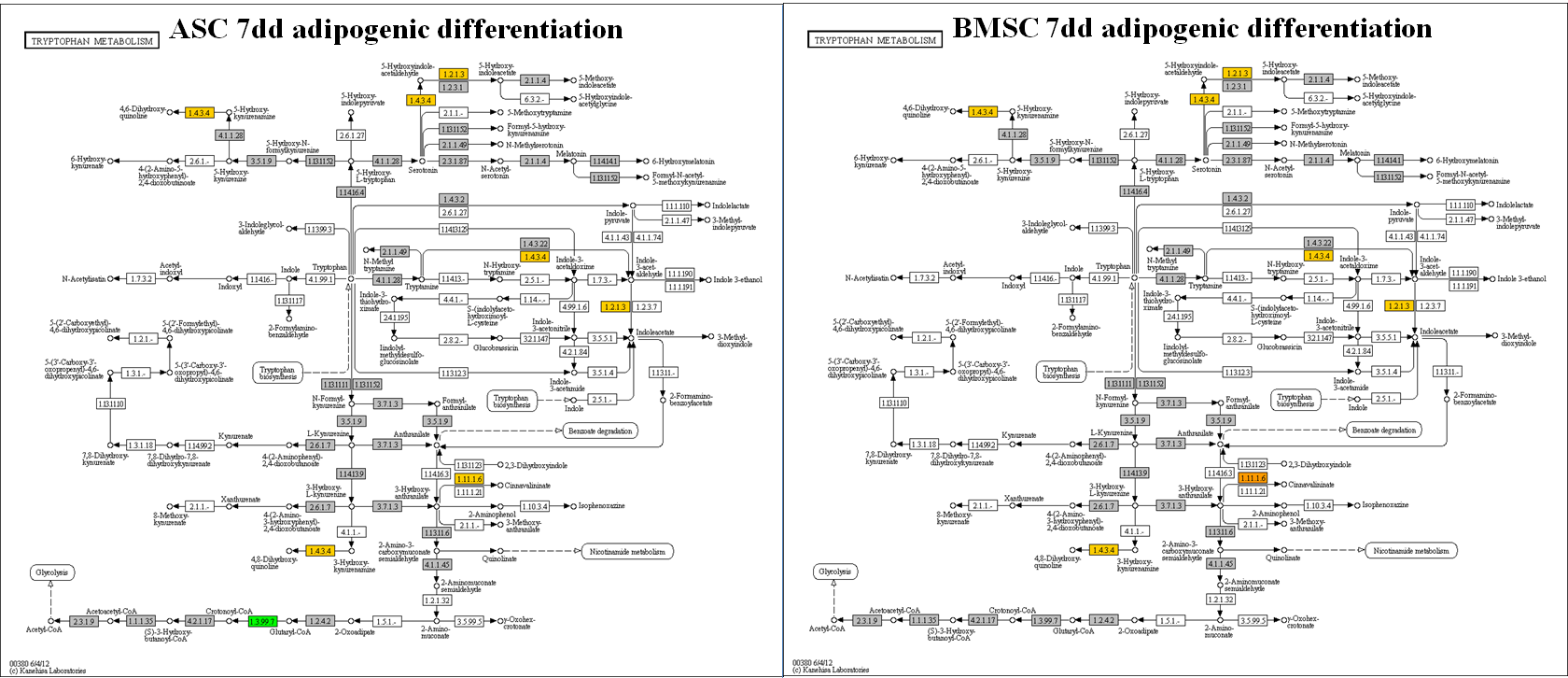

Supplement: S2 Fig — Shown is the response of the KEGG ‘Tryptophan metabolism’ in ASC and BMSC at 7 day of adipogenesis differentiation as obtained by the KegArray tool (http://www.kegg.jp/kegg/download/kegtools.html). Red-orange object denote up-regulation and green down-regulation relative to 0dd. (TIF) [file pone.0137644.s002.tif]

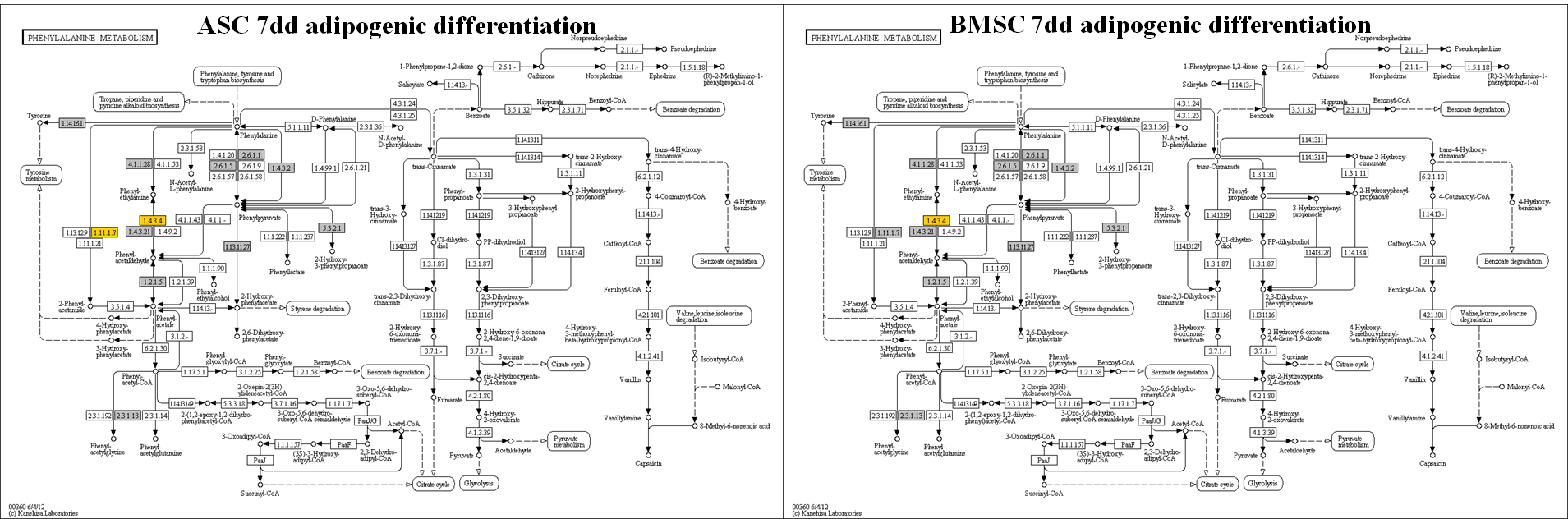

Supplement: S3 Fig — Shown is the difference in response of the KEGG ‘Phenylalanine metabolism’ in ASC and BMSC at 7 day of adipogenesis differentiation as obtained by the KegArray tool (http://www.kegg.jp/kegg/download/kegtools.html). Red-orange object denote up-regulation and green down-regulation relative to 0dd. (TIF) [file pone.0137644.s003.tif]

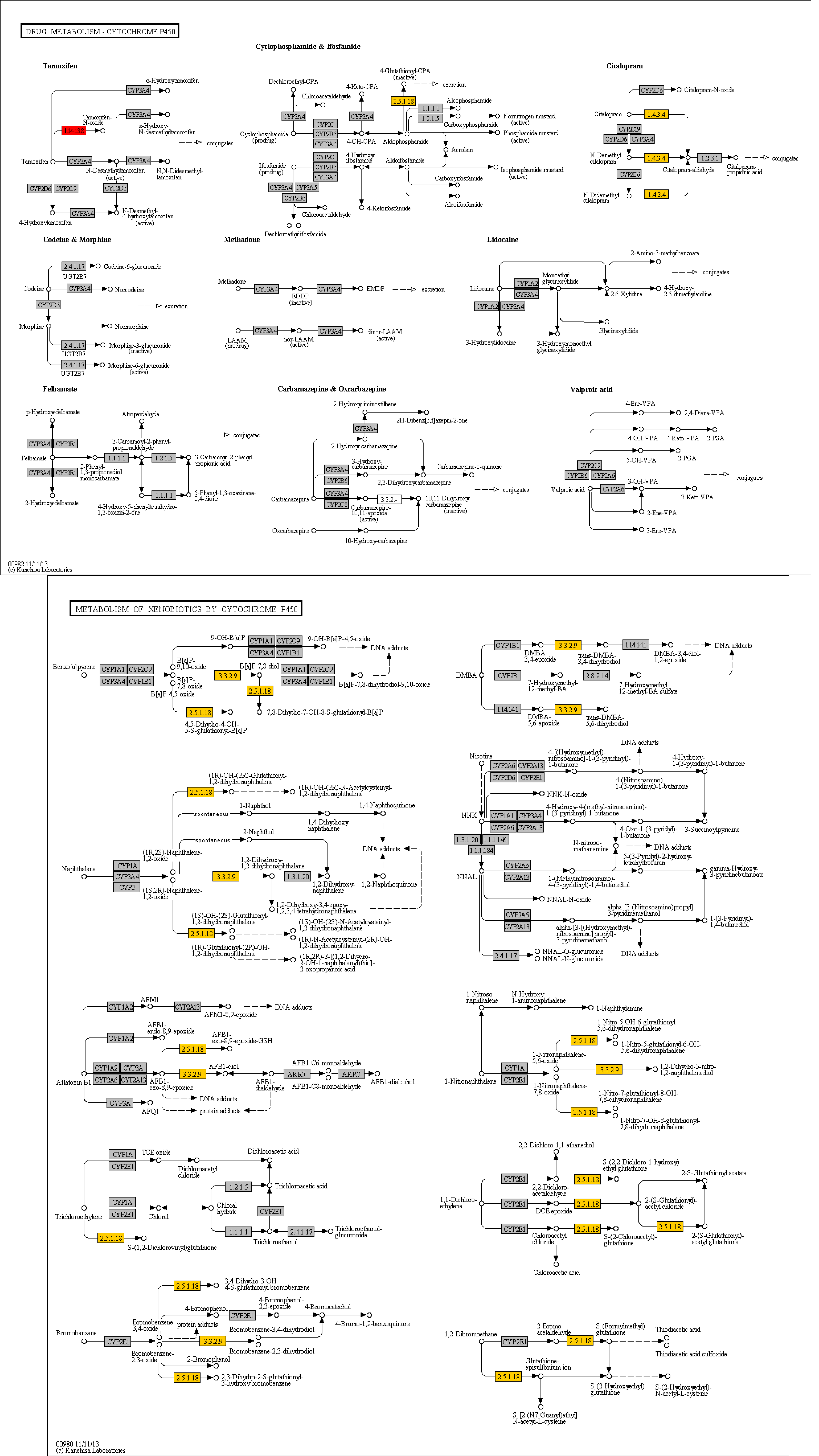

Supplement: S4 Fig — Shown are the figures of the two pathways obtained by the KegArray tool (http://www.kegg.jp/kegg/download/kegtools.html). Red-orange object denote up-regulation and green down-regulation relative to 0dd. (TIFF) [file pone.0137644.s004.tiff]

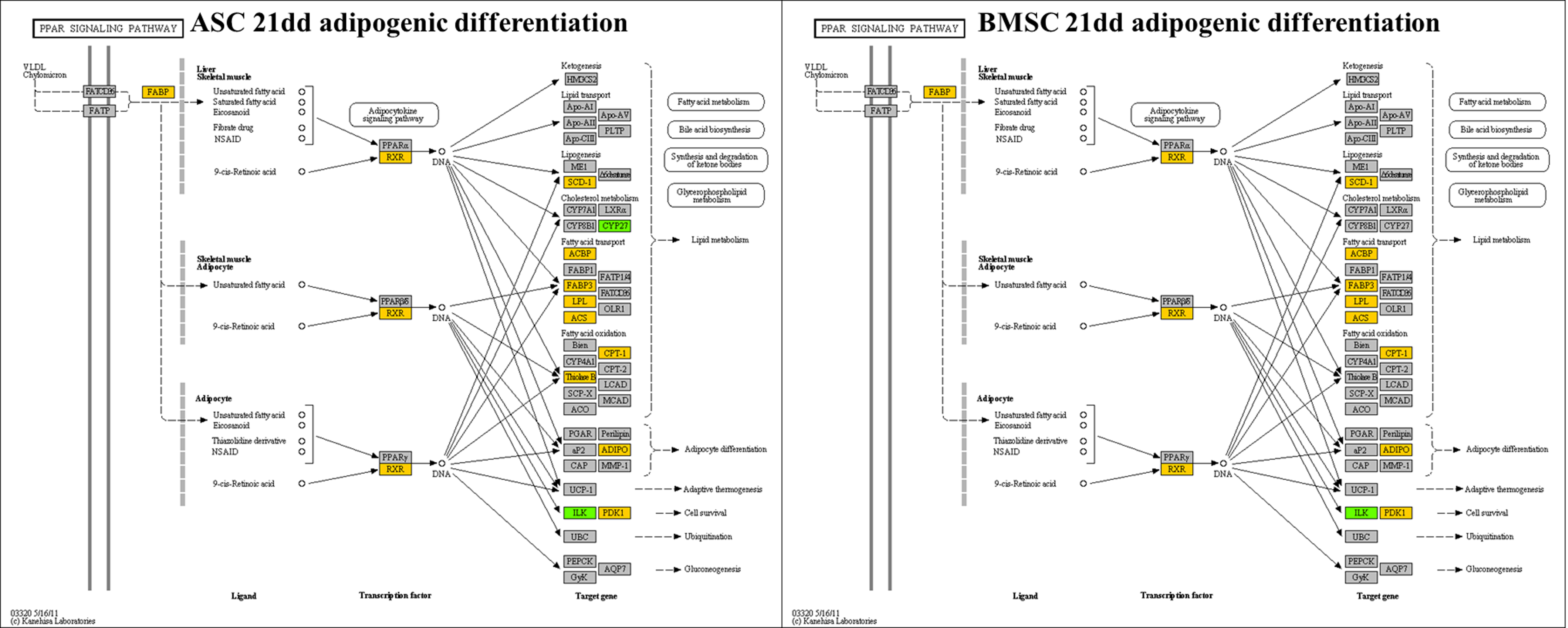

Supplement: S5 Fig — Shown is the KEGG ‘PPAR signaling pathway’ at 21 days of adipogenic differentiation in ASC and BMSC as obtained by the KegArray tool (http://www.kegg.jp/kegg/download/kegtools.html). Striking is the similarity of the response between the two MSC. Red-orange object denote up-regulation and green down-regulation relative to 0dd. (TIF) [file pone.0137644.s005.tif]

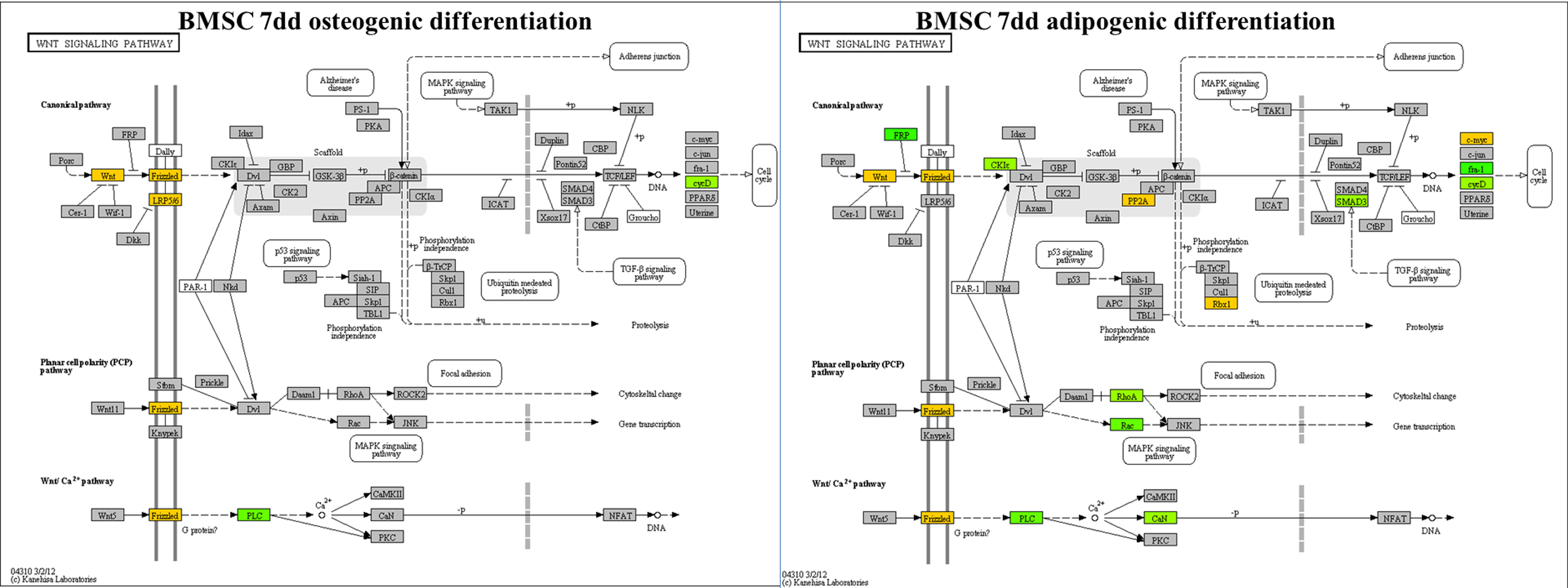

Supplement: S6 Fig — Shown is the response of the KEGG ‘Wnt signaling pathway’ at 7 day of adipogenic and osteogenic differentiation in BMSC as obtained by the KegArray tool (http://www.kegg.jp/kegg/download/kegtools.html). Red-orange object denote up-regulation and green down-regulation relative to 0dd. (TIF) [file pone.0137644.s006.tif]

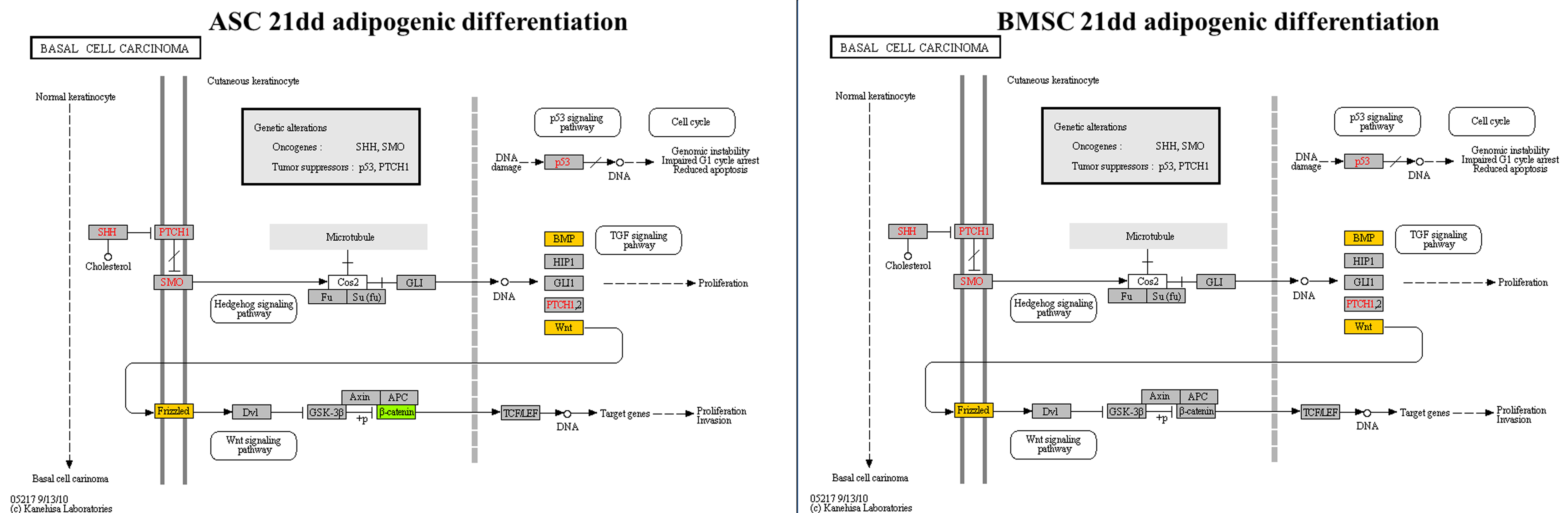

Supplement: S7 Fig — Shown is response of the KEGG ‘Basal cell carcinoma’ in ASC and BMSC at 21 day of adipogenic differentiation as obtained by the KegArray tool (http://www.kegg.jp/kegg/download/kegtools.html). Red-orange object denote up-regulation and green down-regulation relative to 0dd. (TIF) [file pone.0137644.s007.tif]

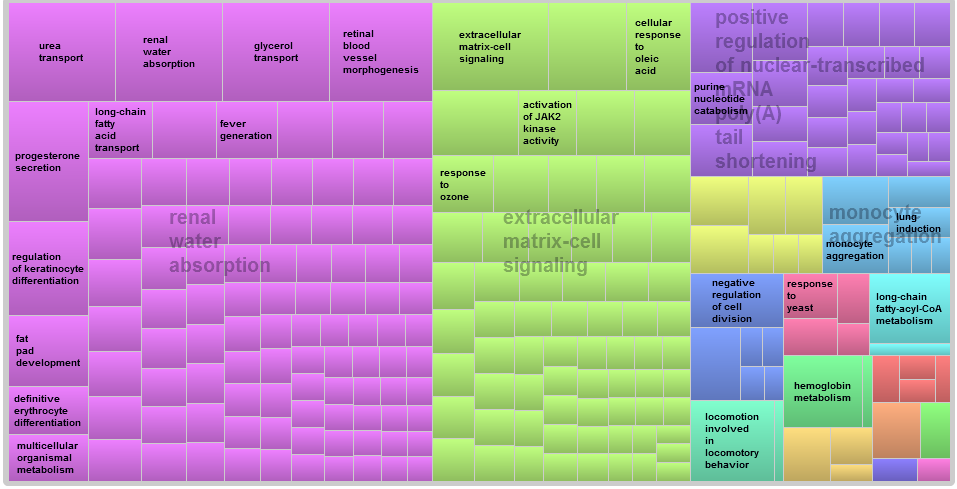

Supplement: S8 Fig — Results are from REVIGO analysis. (TIF) [file pone.0137644.s008.tif]

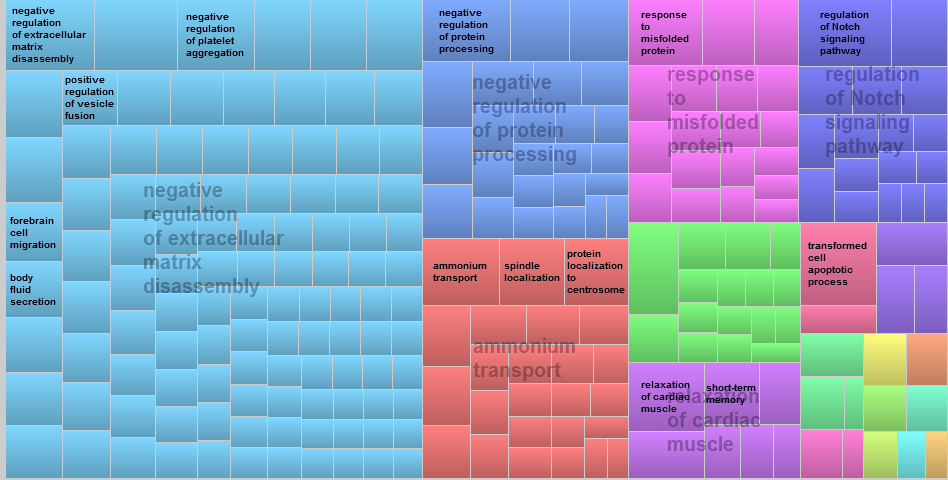

Supplement: S9 Fig — Results are from REVIGO analysis. (TIF) [file pone.0137644.s009.tif]

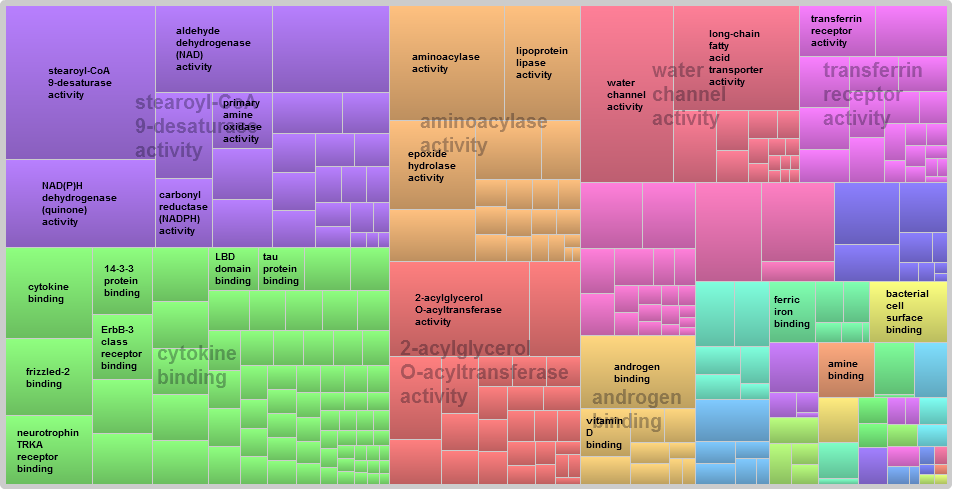

Supplement: S10 Fig — Results are from REVIGO analysis. (TIF) [file pone.0137644.s010.tif]

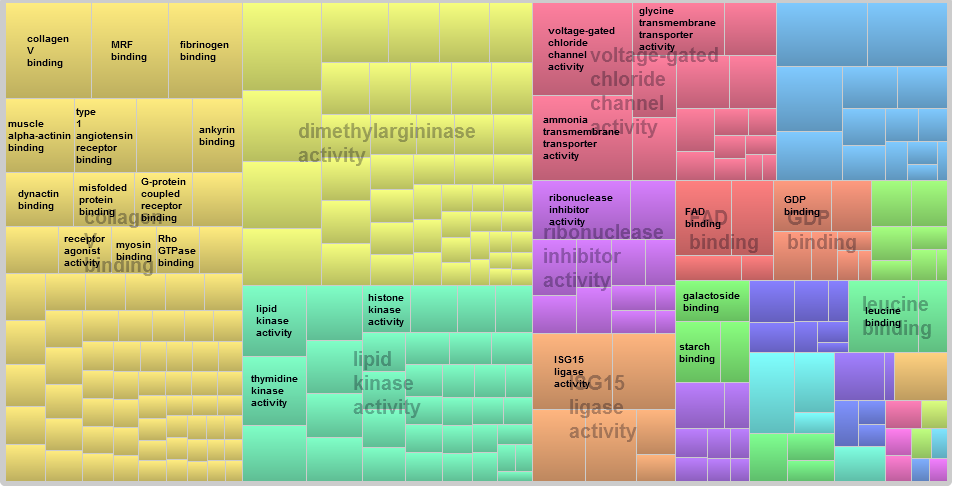

Supplement: S11 Fig — Results are from REVIGO analysis. (TIF) [file pone.0137644.s011.tif]

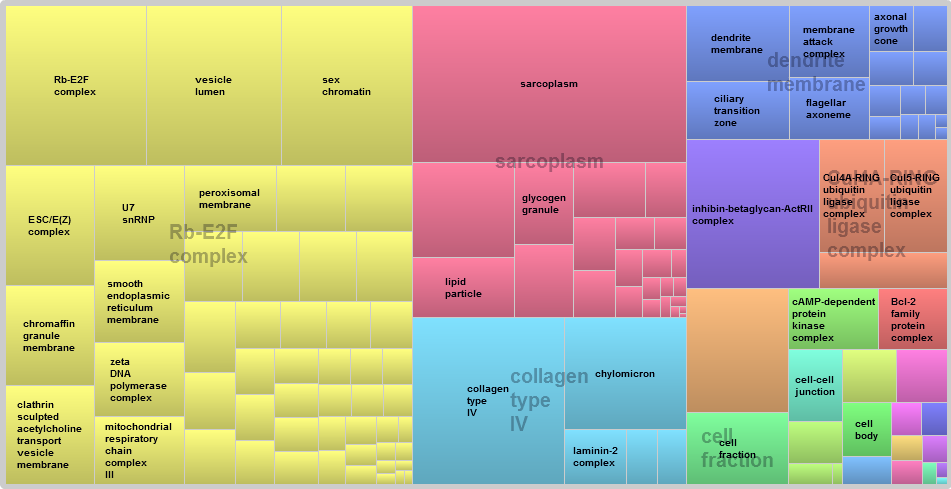

Supplement: S12 Fig — Results are from REVIGO analysis. (TIF) [file pone.0137644.s012.tif]

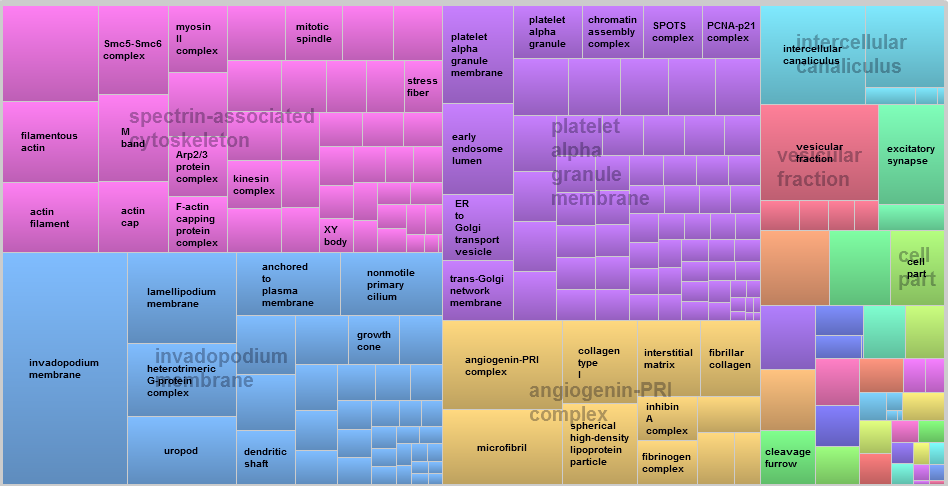

Supplement: S13 Fig — Results are from REVIGO analysis. (TIF) [file pone.0137644.s013.tif]

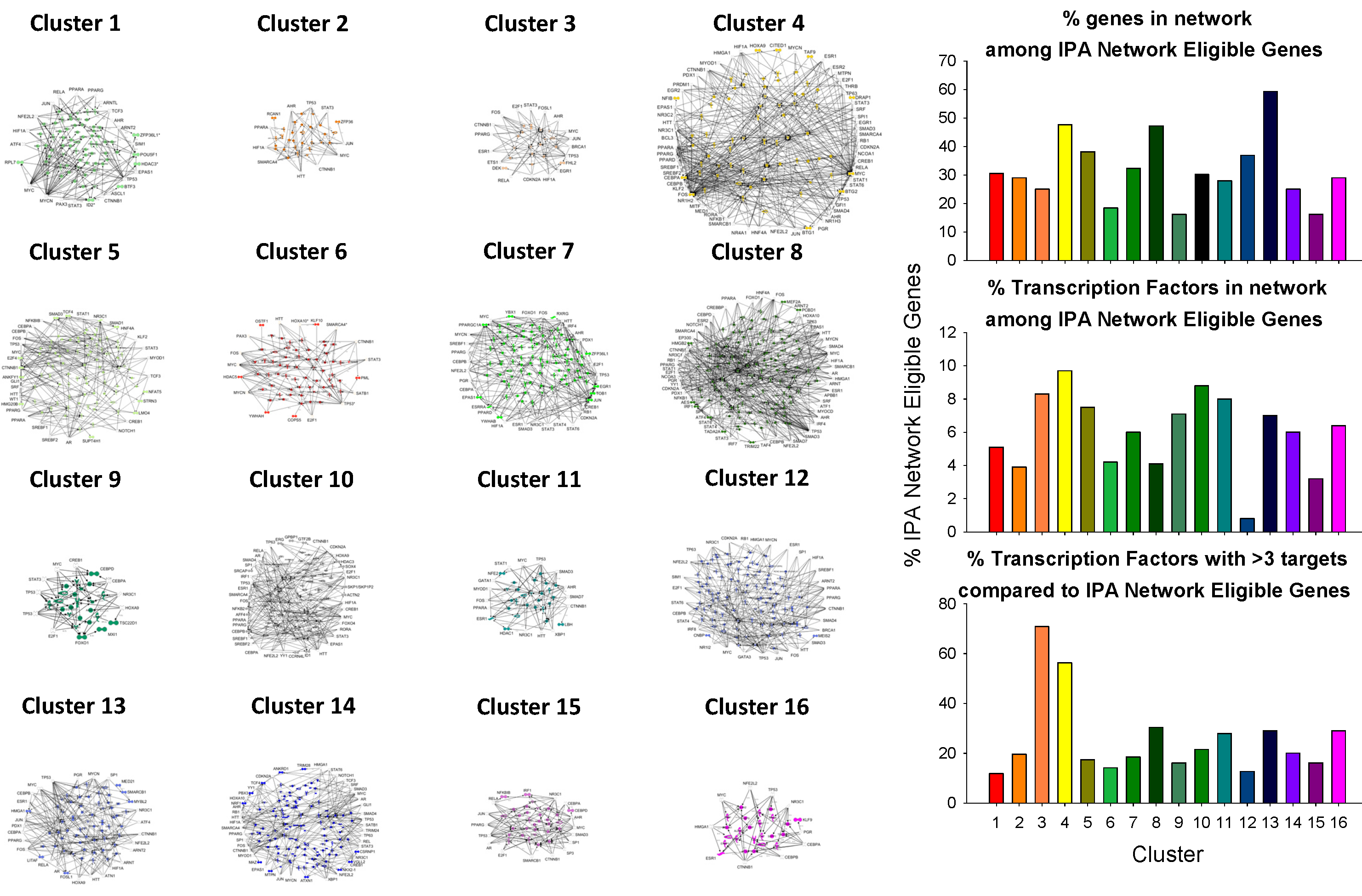

Supplement: S15 Fig — In the left are shown the interactive networks among genes in each cluster constructed using Ingenuity Pathway Analysis (IPA). Details for each network are provided in S9 File. The graphs on the right denote: upper panel = the % of genes present in the network among all genes in the cluster eligible for network analysis in IPA; middle panel = the % of transcription factors (TF) present in the network among all genes in the cluster eligible for network analysis in IPA; bottom panel = the % of all TF with ≥3 down-stream genes (both present in the cluster and with a putative effect on transcription of genes included in the cluster) relative to all genes in the cluster eligible for network analysis in IPA. (TIF) [file pone.0137644.s015.tif]
