# Supplementary material for: Transcription Adaptation during In Vitro Adipogenesis and Osteogenesis of Porcine Mesenchymal Stem Cells: Dynamics of Pathways, Biological Processes, Up-Stream Regulators, and Gene Networks
Source: PLoS One. 2015 Sep 23;10(9):e0137644. doi: 10.1371/journal.pone.0137644 (PMC4580618; doi:10.1371/journal.pone.0137644)
Supplement: S9 File — The PDF file contains the high quality image of the network among genes of each of the 16 cluster plus the transcription factor with ≥3 down-stream molecules. The genes belonging to the cluster are colored as the color of the cluster (see Fig 7). In the periphery of the network are reported the transcription factors (TF; with a larger font). The ones with the colored object are present in the cluster. The ones with white object are TF not present in the cluster but with ≥3 down-stream target among genes in the cluster as uncovered by Ingenuity Pathway Analysis. (PDF) [file pone.0137644.s024.pdf]

IPA Network Eligible Molecules	118
Type of Relationships	
Total	37
Genes	36
% genes	30.5
**Transcription Factor**	
Total	18
In cluster	6
% molecules	5.1
No cluster	12

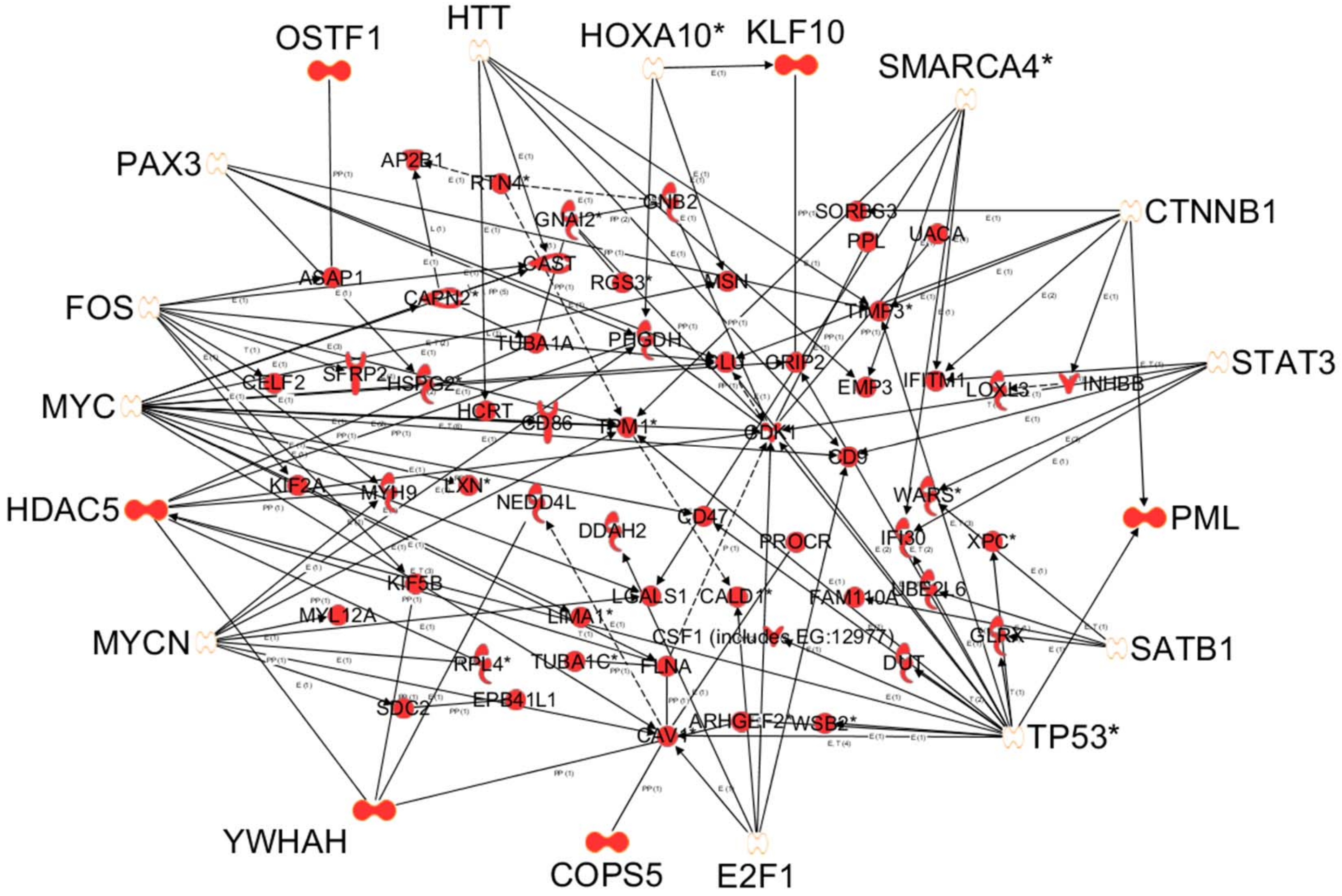

Cluster 1

IPA Network Eligible Molecules	51
Type of Relationships	
Total	16
Genes	17
% genes	29.0
**Transcription Factor**	
Total	12
In cluster	2
% molecules	3.9
No cluster	10

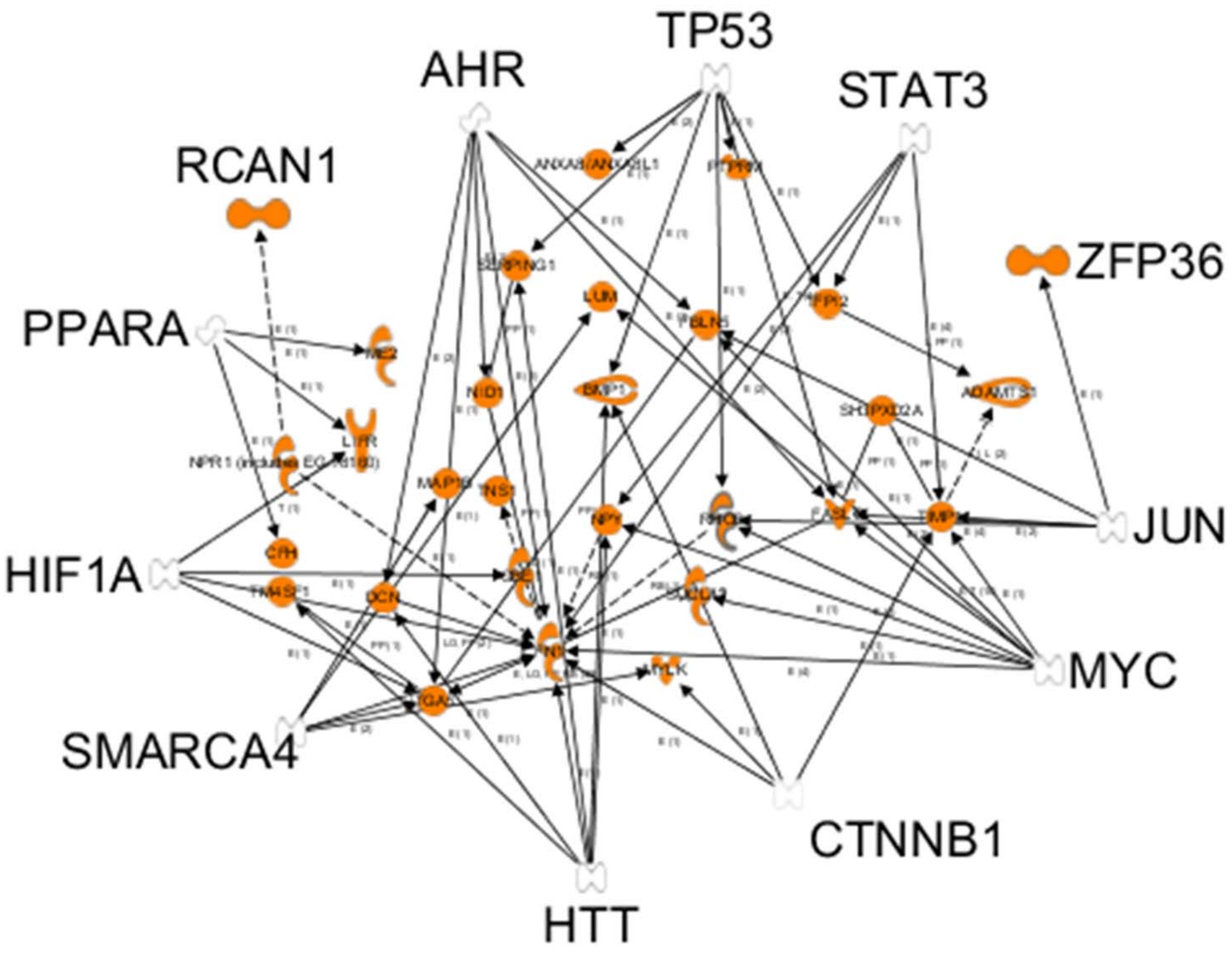

Cluster 2

IPA Network Eligible Molecules	24
**Type of Relationships**	
Total	6
Genes	6
% genes	25.0
**Transcription Factor**	
Total	19
In cluster	2
% molecules	8.3
No cluster	17

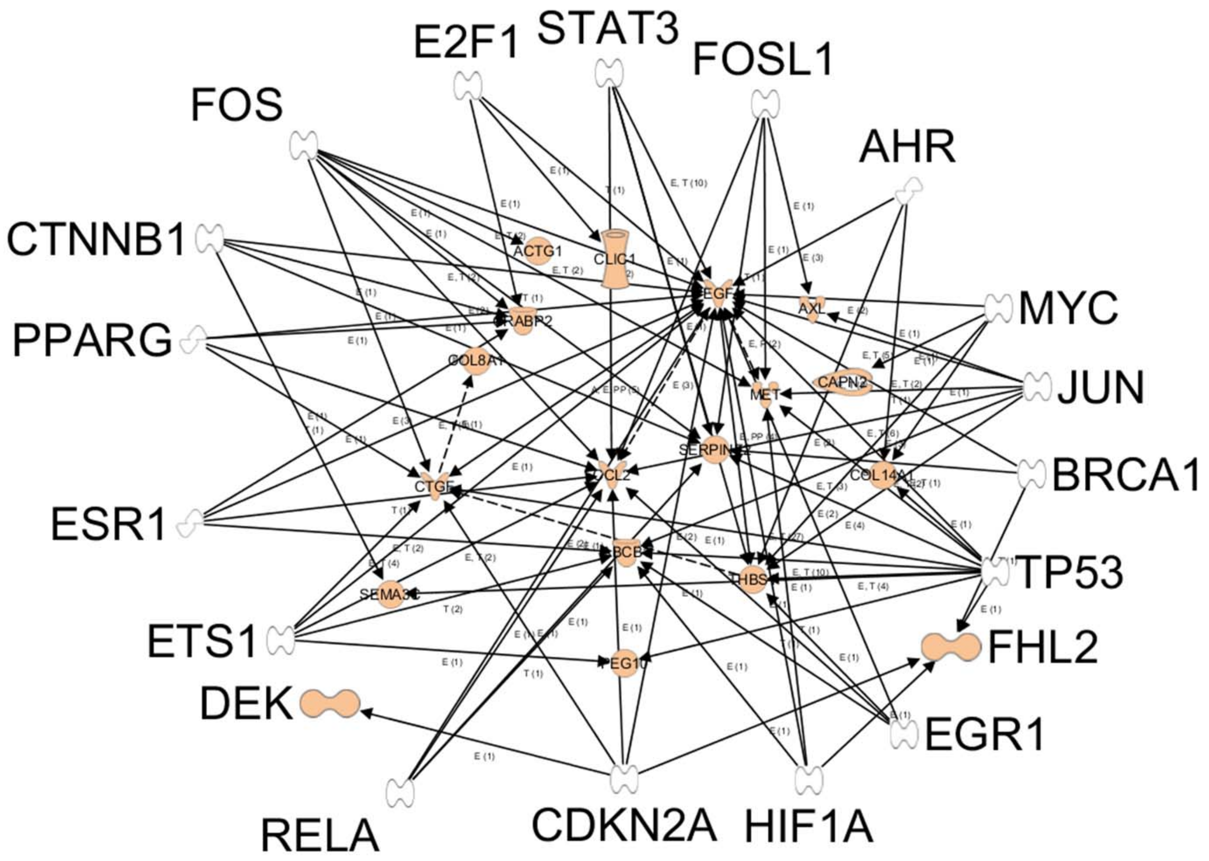

Cluster 3

IPA Network Eligible Molecules	103
Type of Relationships	
Total	99
Genes	49
% genes	47.6
**Transcription Factor**	
Total	65
In cluster	10
% molecules	9.7
No cluster	55

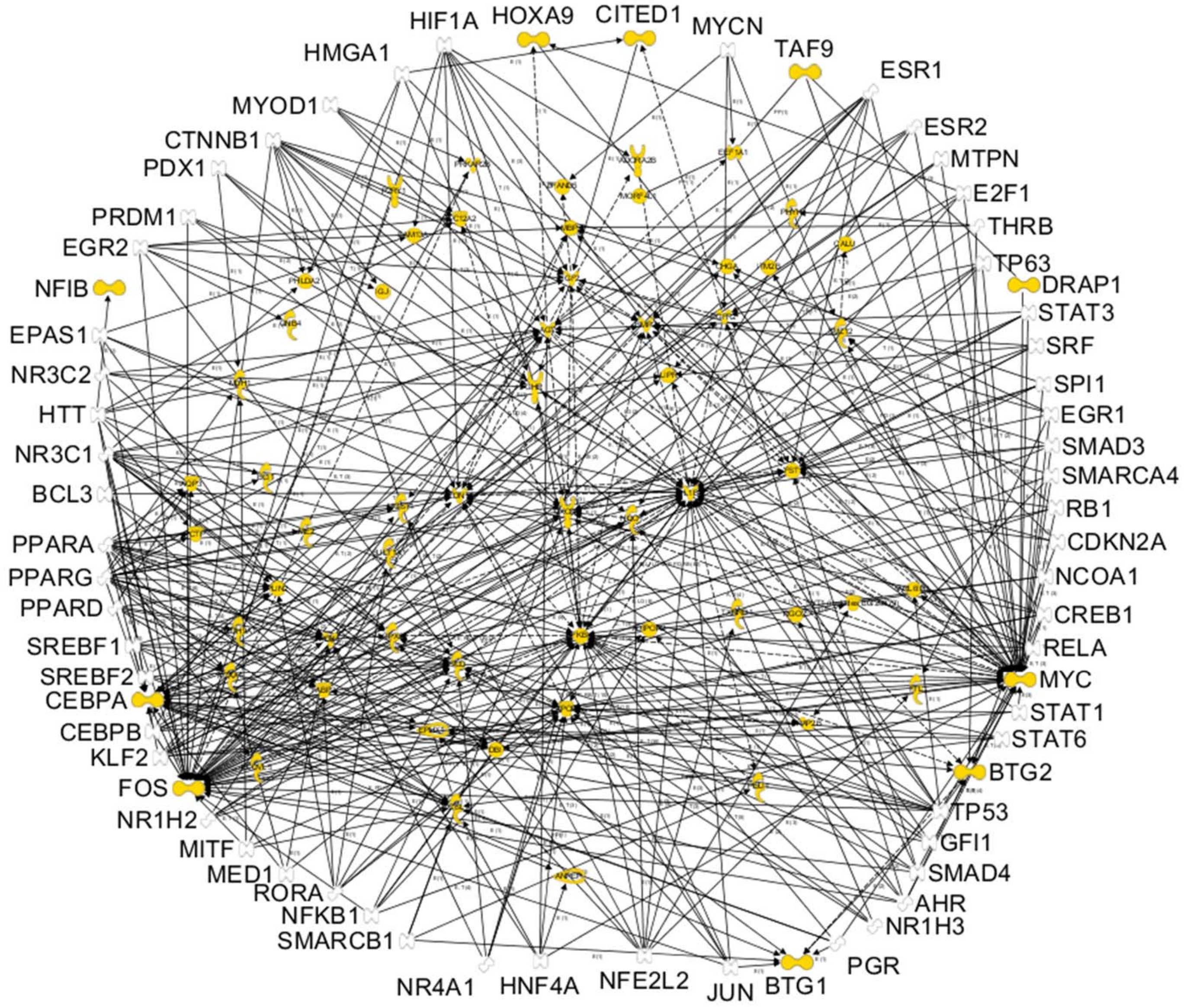

Cluster 4

IPA Network Eligible Molecules	160
**Type of Relationships**	
Total	81
Genes	61
% genes	38.1
**Transcription Factor**	
Total	35
In cluster	12
% molecules	7.5
No cluster	23

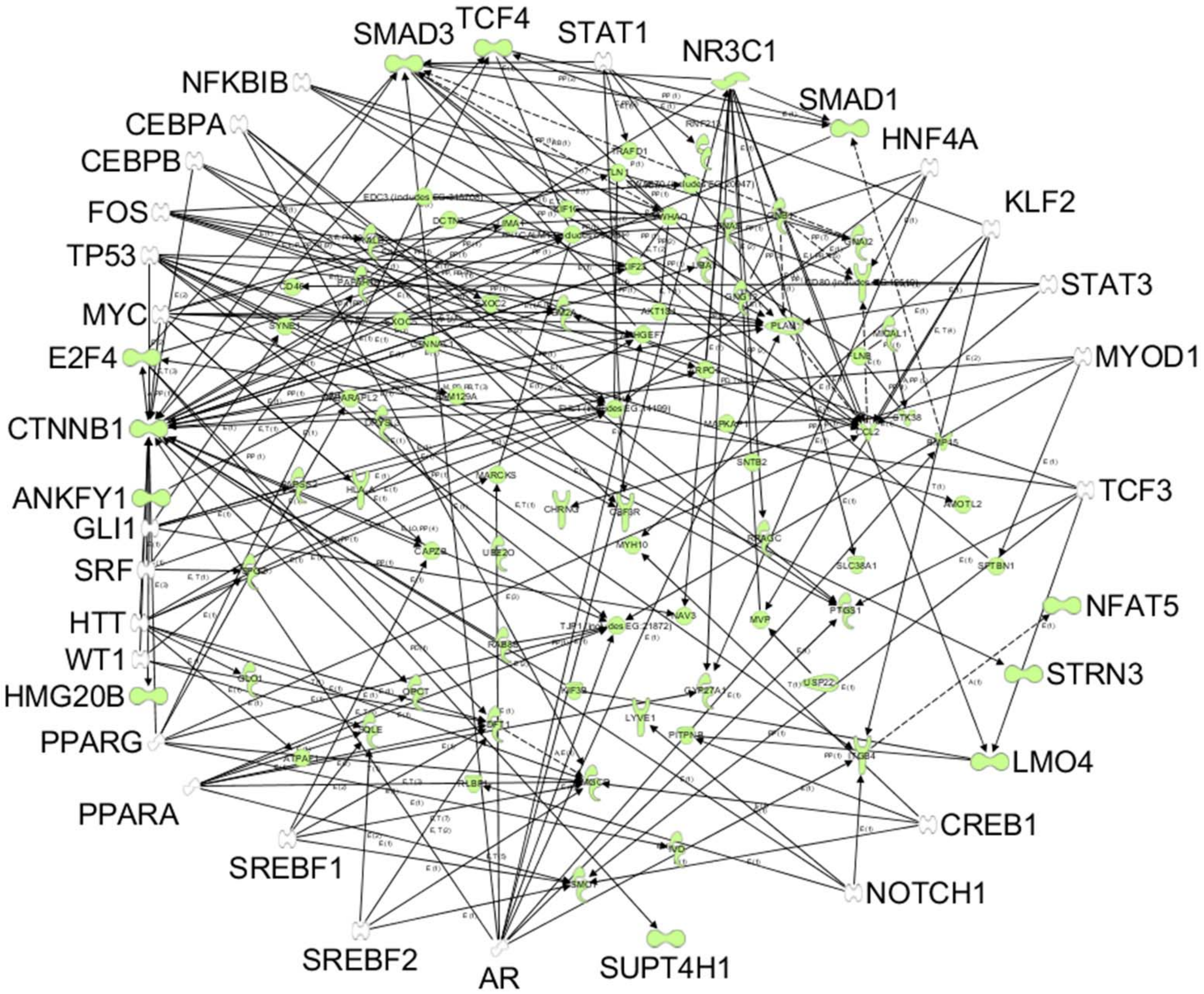

Cluster 5

IPA Network Eligible Molecules	141
**Type of Relationships**	
Total	23
Genes	26
% genes	18.4
**Transcription Factor**	
Total	26
In cluster	6
% molecules	4.2
No cluster	20

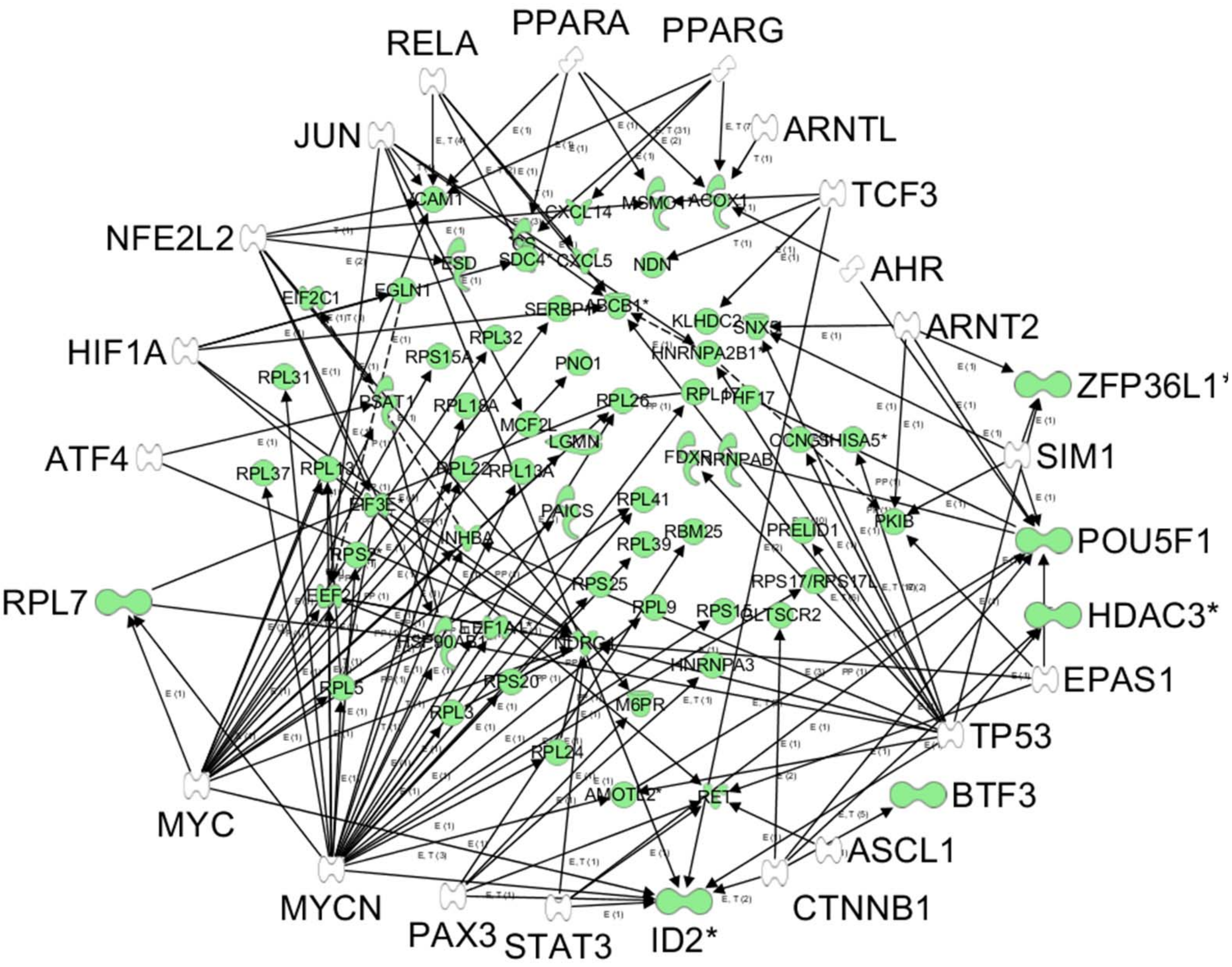

Cluster 6

IPA Network Eligible Molecules	167
Type of Relationships	
Total	61
Genes	54
% genes	32.3
**Transcription Factor**	
Total	37
In cluster	10
% molecules	6.0
No cluster	27

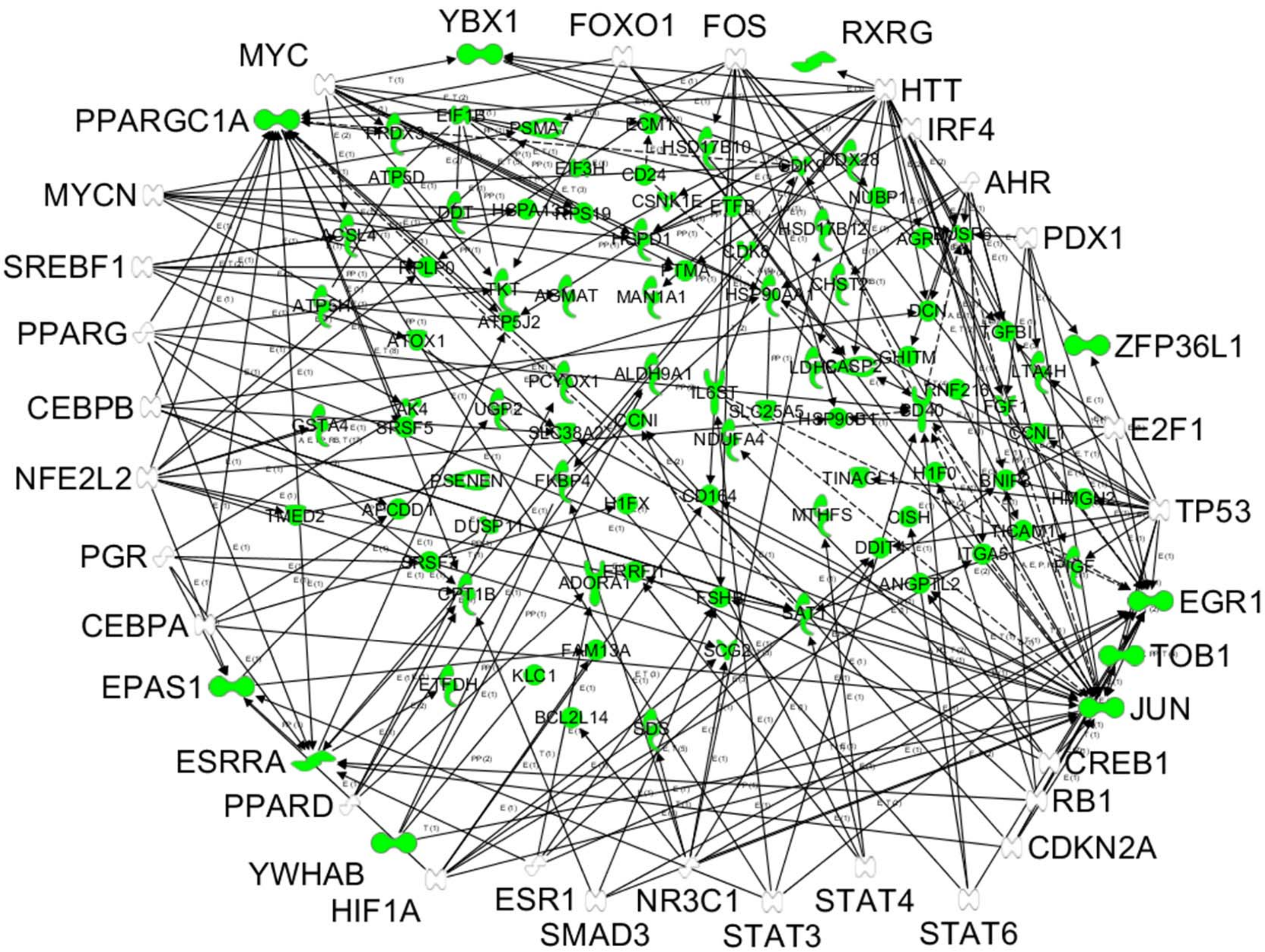

Cluster 7

IPA Network Eligible Molecules	194
**Type of Relationships**	
Total	169
Genes	92
% genes	47.2
**Transcription Factor**	
Total	64
In cluster	8
% molecules	4.1
No cluster	56

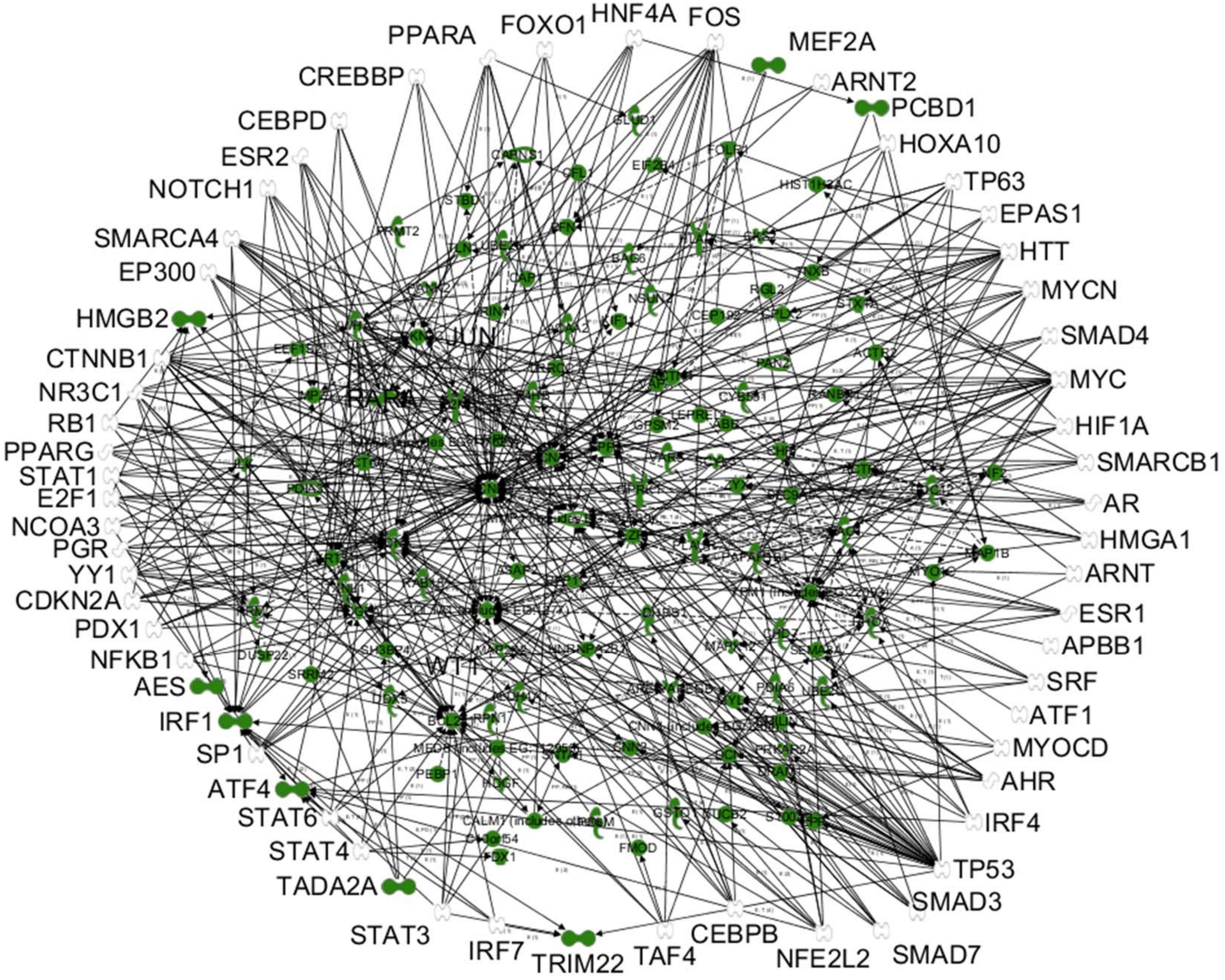

Cluster 8

IPA Network Eligible Molecules	56
Type of Relationships	
Total	11
Genes	9
% genes	16.1
**Transcription Factor**	
Total	13
In cluster	4
% molecules	2.7
No cluster	9

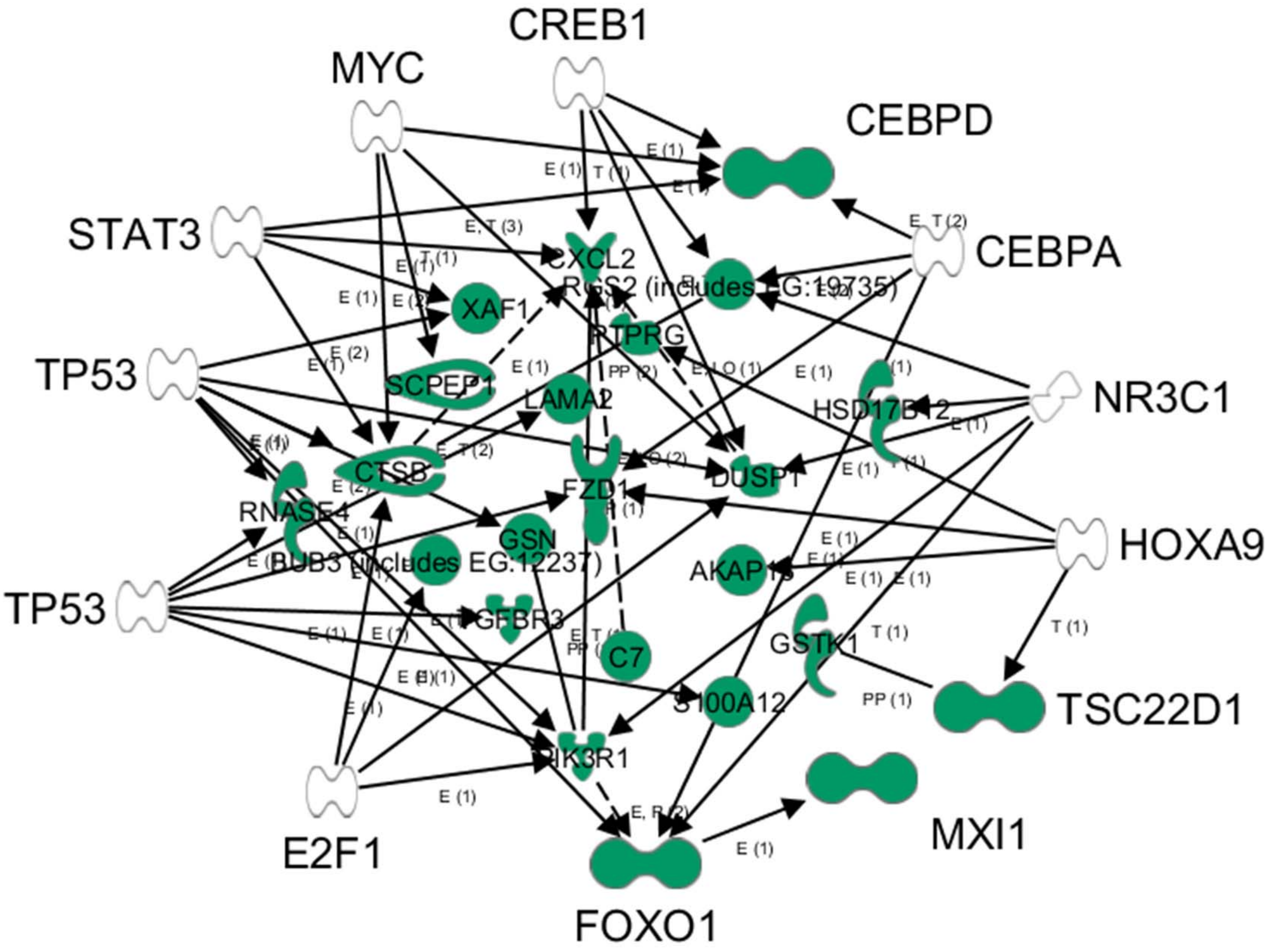

Cluster 9

IPA Network Eligible Molecules	148
Type of Relationships	
Total	70
Genes	54
% genes	30.2
**Transcription Factor**	
Total	43
In cluster	13
% molecules	8.8
No cluster	30

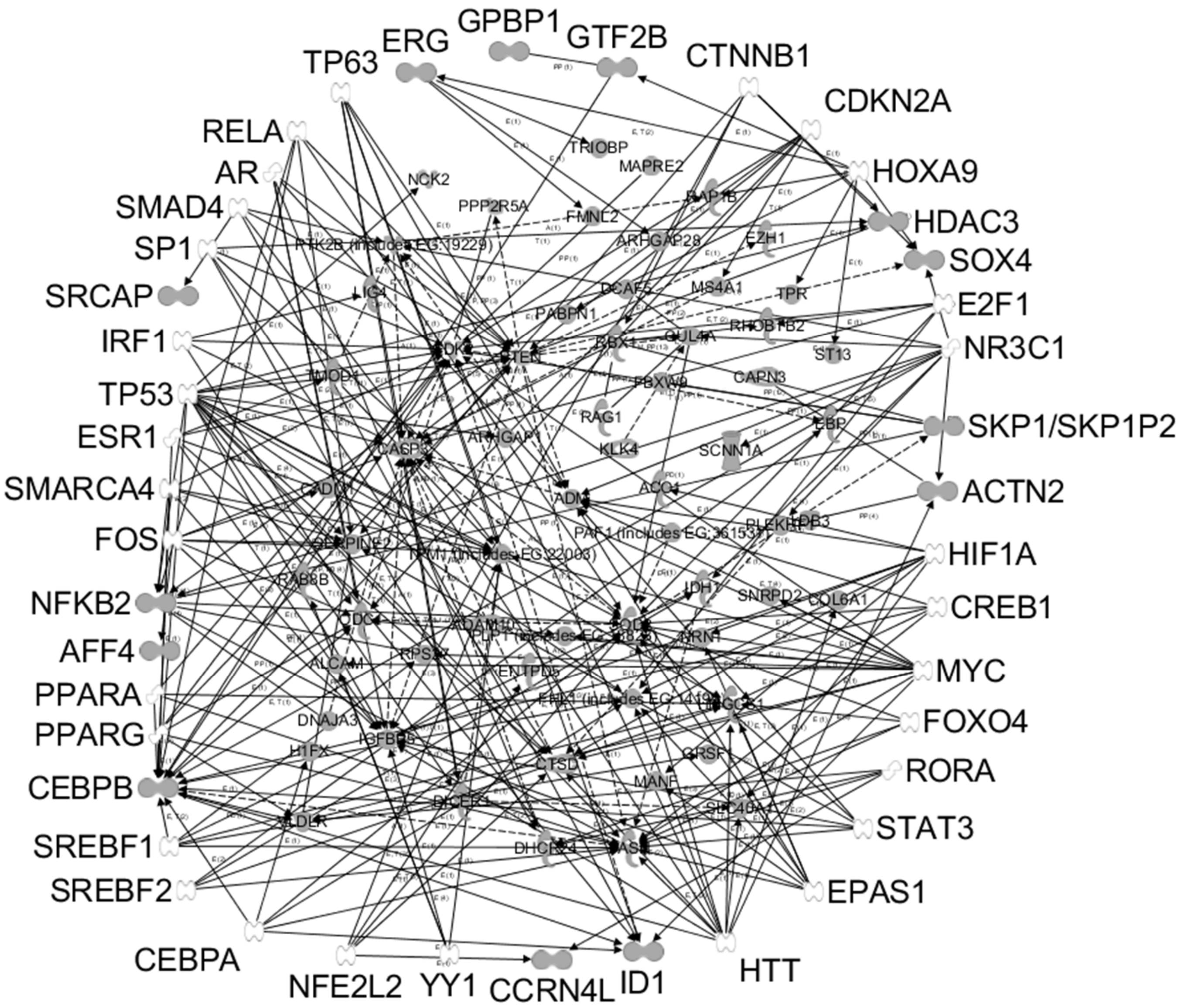

Cluster 10

IPA Network Eligible Molecules	50
Type of Relationships	
Total	13
Genes	14
% genes	28
**Transcription Factor**	
Total	18
In cluster	4
% molecules	8.0
No cluster	14

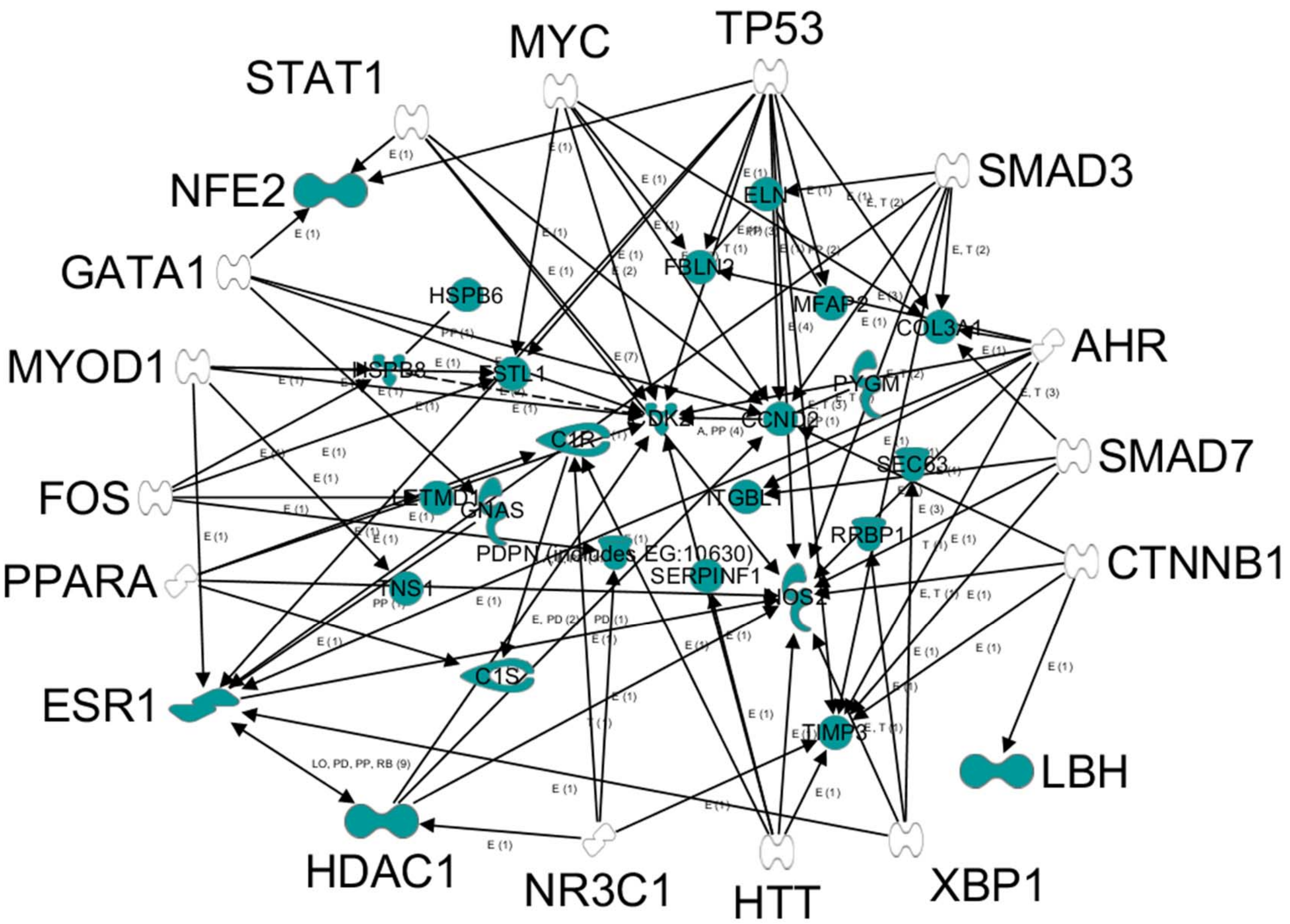

Cluster 11

IPA Network Eligible Molecules	244
**Type of Relationships**	
Total	119
Genes	90
% genes	36.9
**Transcription Factor**	
Total	33
In cluster	2
% molecules	0.8
No cluster	31

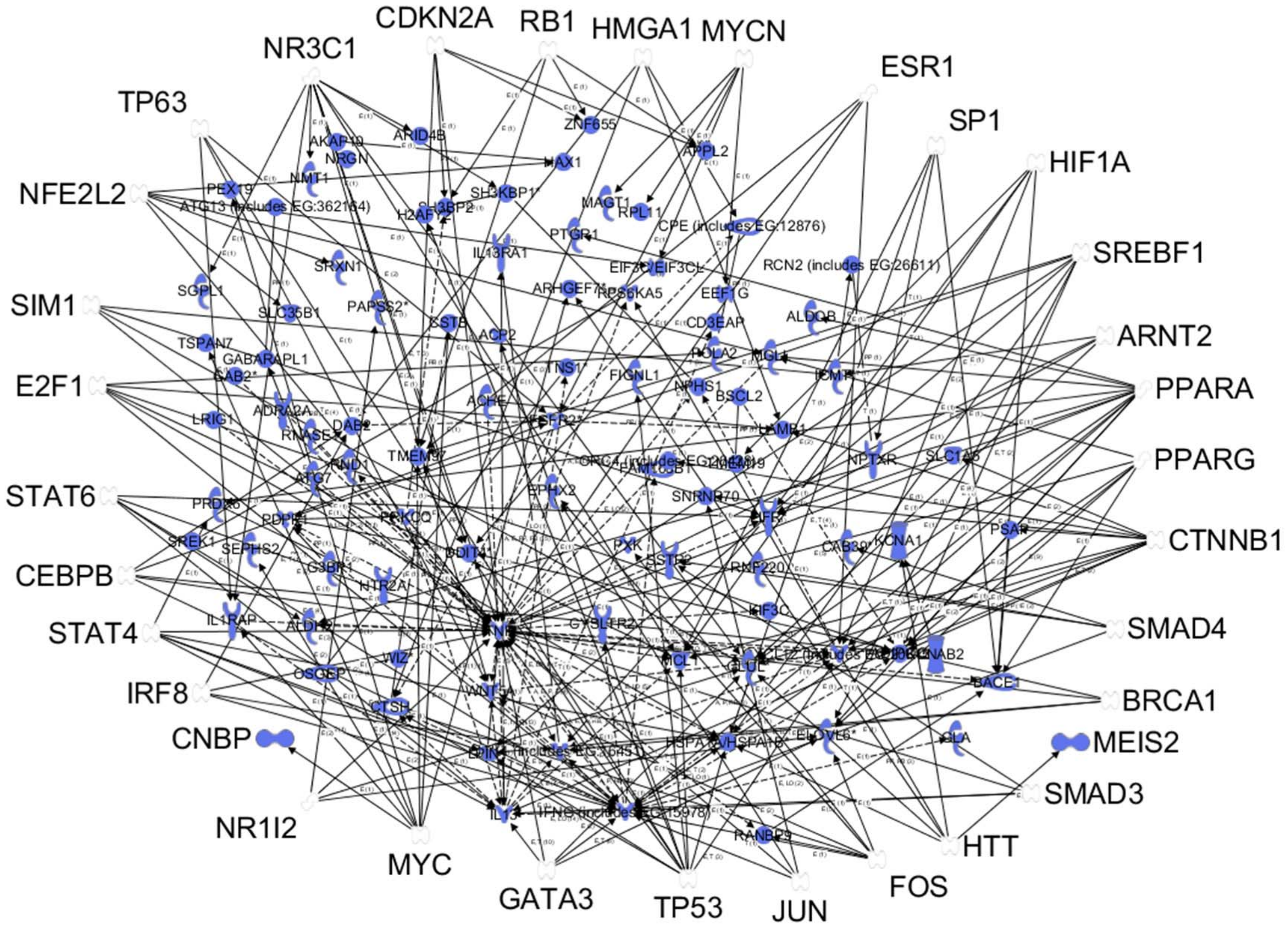

Cluster 12

IPA Network Eligible Molecules	86
Type of Relationships	
Total	86
Genes	51
% genes	59.3
**Transcription Factor**	
Total	30
In cluster	6
% molecules	7.0
No cluster	24

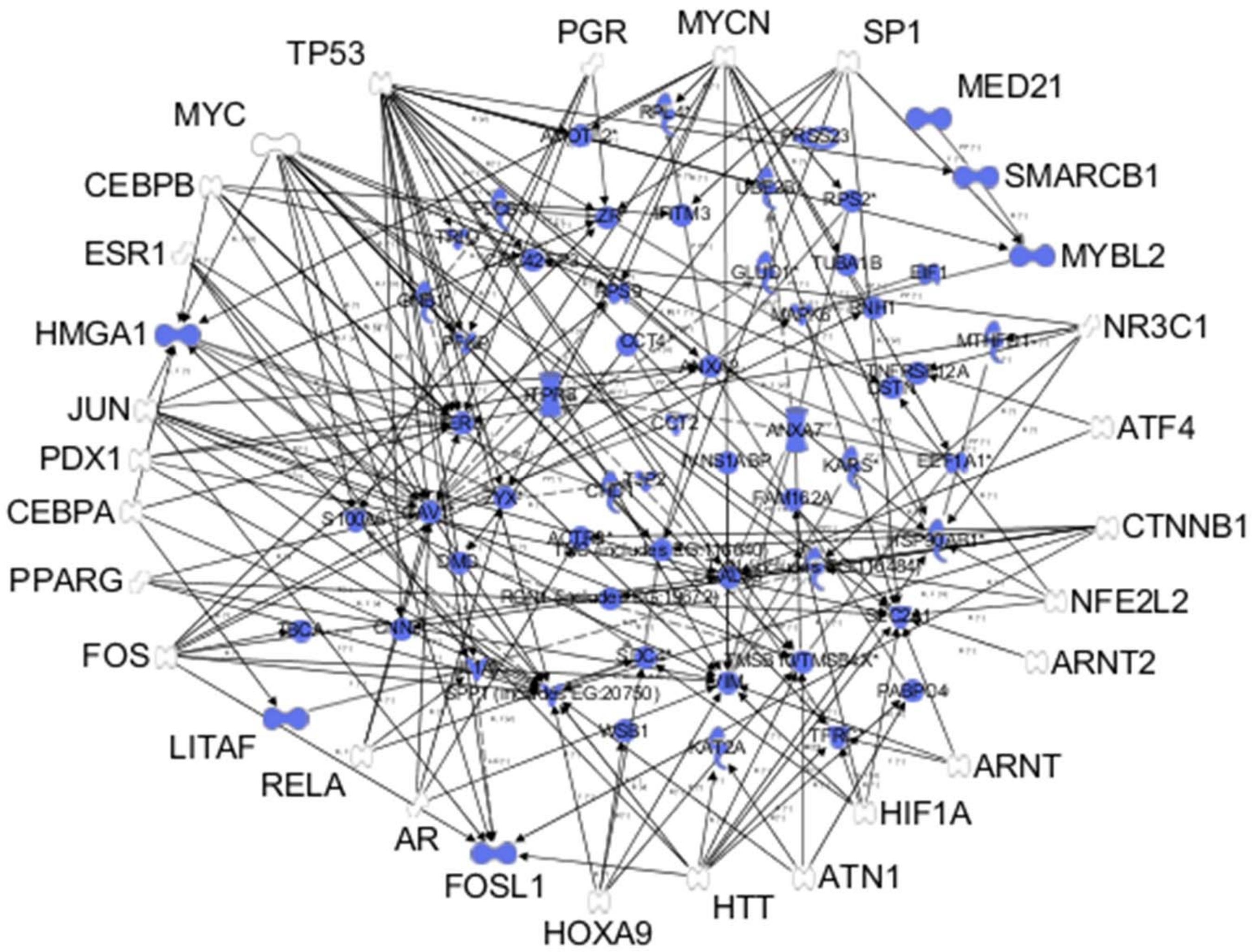

Cluster 13

IPA Network Eligible Molecules	184
Type of Relationships	
Total	39
Genes	46
% genes	25.0
**Transcription Factor**	
Total	47
In cluster	11
% molecules	6.0
No cluster	36

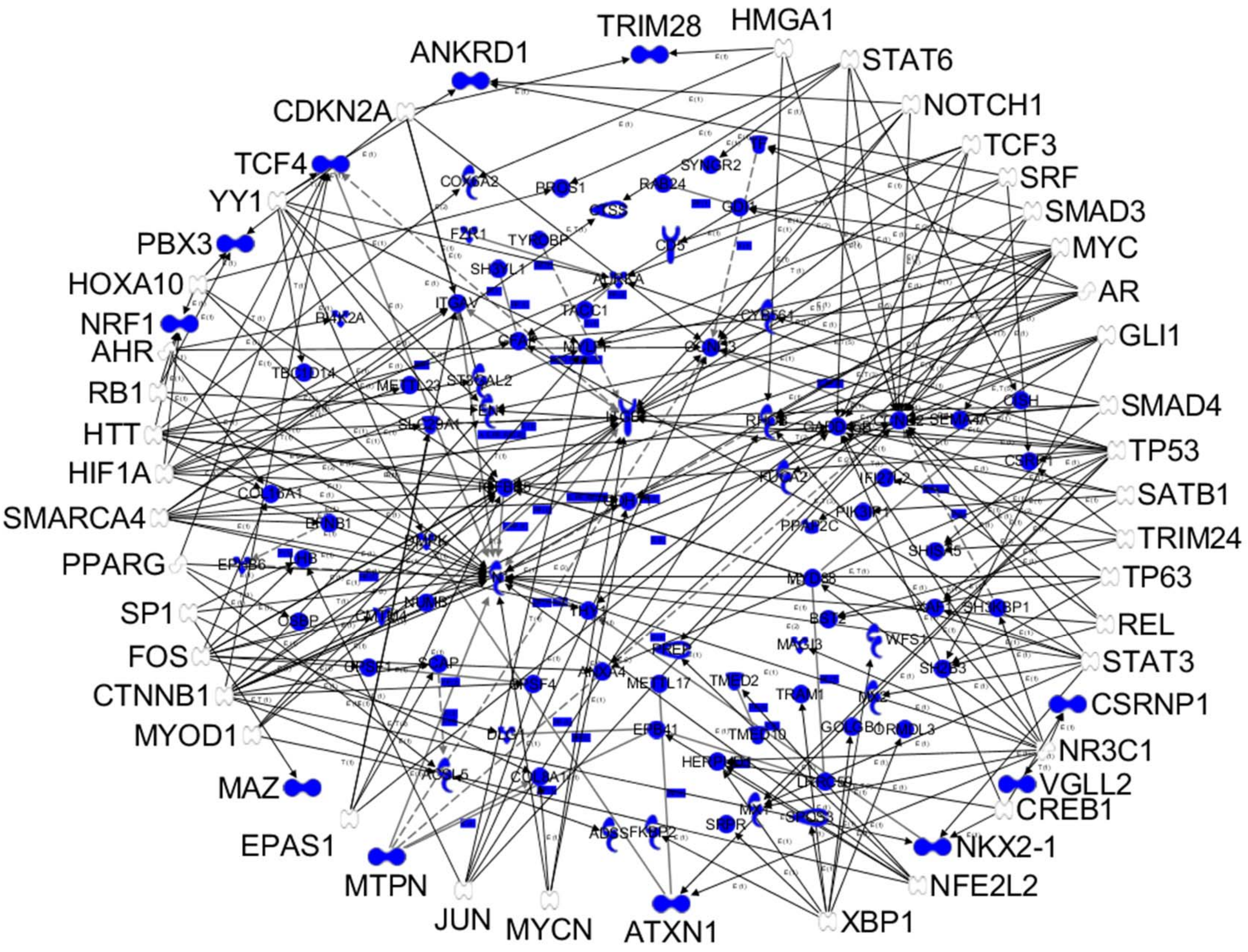

Cluster 14

IPA Network Eligible Molecules	93
Type of Relationships	
Total	11
Genes	15
% genes	16.1

Transcription Factor	
Total	18
In cluster	3
% molecules	3.2
No cluster	15

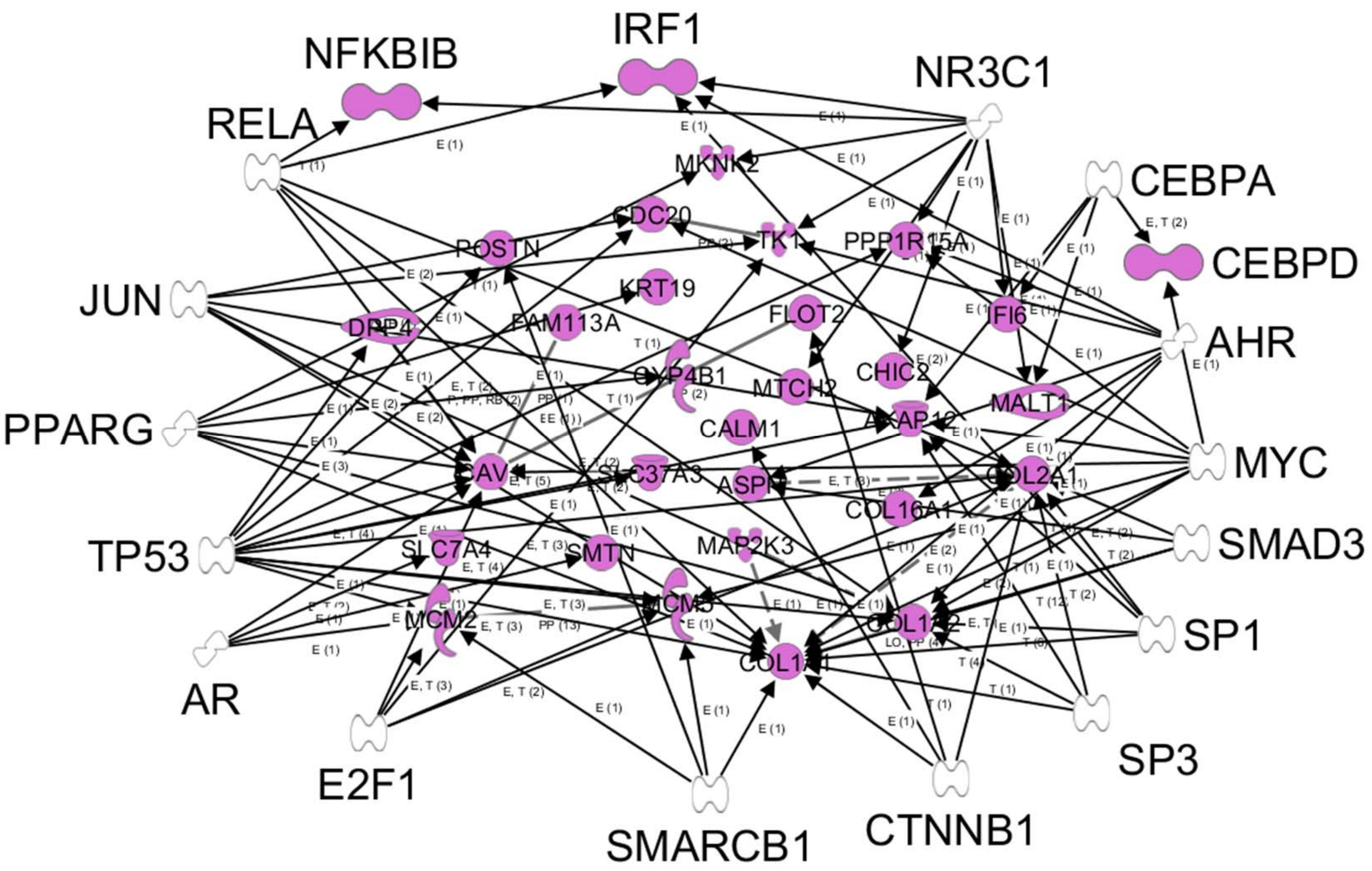

Cluster 15

IPA Network Eligible Molecules	31
**Type of Relationships**	
Total	7
Genes	9
% genes	29.0
**Transcription Factor**	
Total	11
In cluster	2
% molecules	6.4
No cluster	9

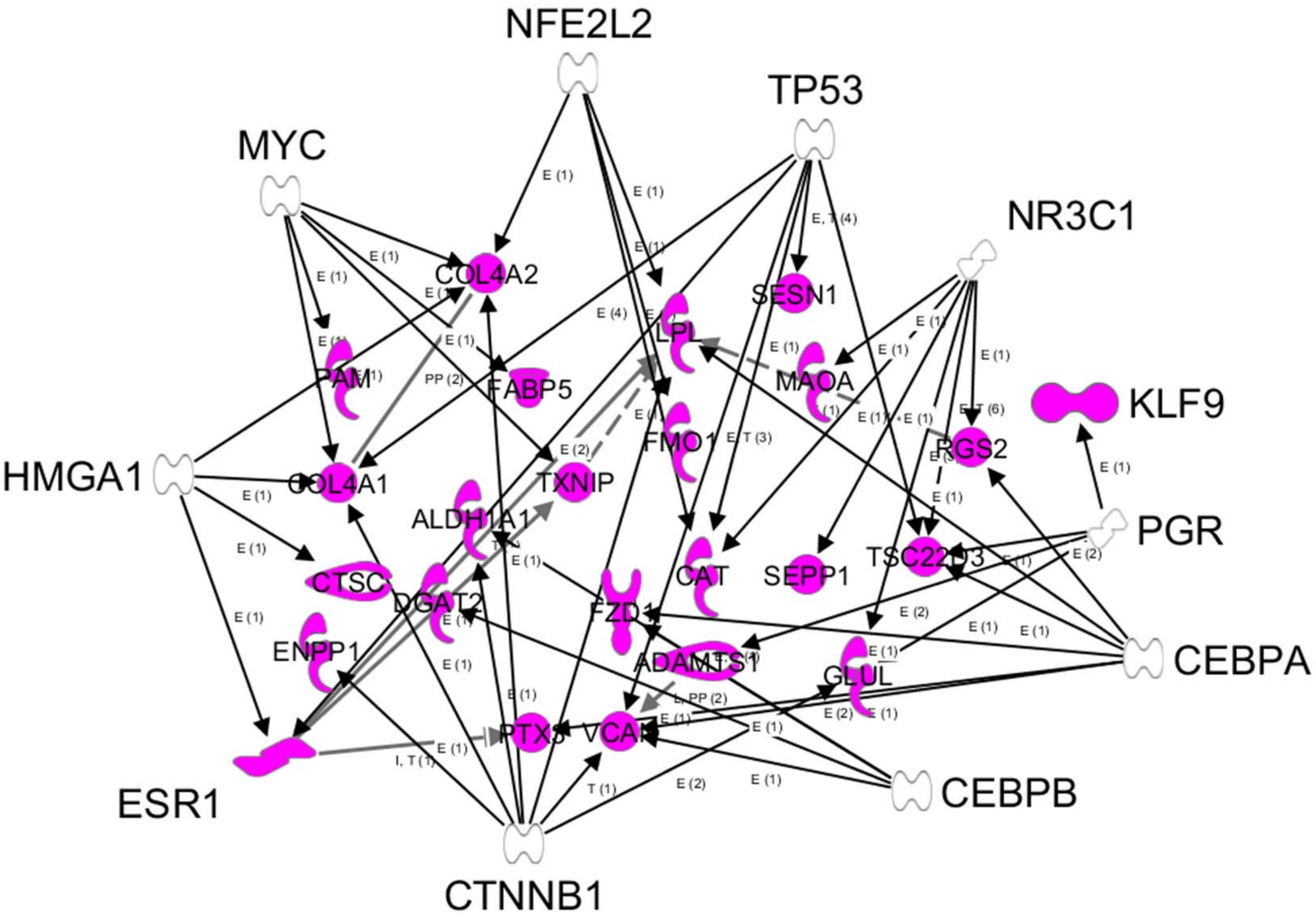

Cluster 16
