# Supplementary material for: Transcription Adaptation during In Vitro Adipogenesis and Osteogenesis of Porcine Mesenchymal Stem Cells: Dynamics of Pathways, Biological Processes, Up-Stream Regulators, and Gene Networks
Source: PLoS One. 2015 Sep 23;10(9):e0137644. doi: 10.1371/journal.pone.0137644 (PMC4580618; doi:10.1371/journal.pone.0137644)
Supplement: S11 File — This is a supplemental discussion of clusters of grouped genes with overall changes in expression during adipogenic compared to osteogenic differentiation. (DOCX) [file pone.0137644.s026.docx]

**Supplemental discussion of κ-mean cluster analysis**

Bionaz et al., PLoS ONE

The clusters 4, 7 and 12 grouped genes with an overall larger increase in expression during adipogenesis compared to osteogenesis. When using the liberal EASE score to determine the enrichment of biological terms, the cluster 4 and 12 were enriched with genes involved in lipid metabolism (File S8 and File S8), but only cluster 4 was enriched with genes involved in fatty acid metabolism and response to organic substrate with a Benjamini-Hochberg FDR< 0.05 for the significance of the enrichment. The response to organic substrates included 19 genes; most of those were involved in lipid synthesis and accumulation (File S7). The cluster 4 appears to be the cluster “signature” of the adipogenic differentiation due to its large difference between the two differentiation types. Interestingly, despite the large number of potential PPARγ target genes in the cluster, the KEGG pathway associated with PPAR signaling was enriched significantly (<0.05) only using the EASE score (File S7). Cluster 4 was also enriched by genes typical of monocytes. There is not an apparent explanation for this observation. Cluster 7 was highly enriched with genes involved in the mitochondria membrane. Interestingly, the mitochondria are highly affected by the adipogenesis, as previously observed in T3T cells, with overall increases in mitochondrial biogenesis and uncoupling respiration [[1](#_ENREF_1),[2](#_ENREF_2)].

The clusters 11, 14, and 15 grouped genes that expression is increased during osteogenesis and reduced during adipogenesis. Among those only cluster 15 had an enrichment of collagen- and extracellular matrix-associated genes with a BH FDR<0.05 (Figure 7 and File S7). Those genes can be considered expected during osteogenesis bearing in mind that collagen type I deposition in the extracellular matrix is essential for bone structure [[3](#_ENREF_3)]. The cluster 15 appears to contain the “osteogenic signature genes”, based on the larger increase in expression pattern of those genes during osteogenesis compared to adipogenesis. The cluster 11 also grouped genes with a larger increase in expression during osteogenic vs. adipogenic differentiation. This cluster grouped genes that had an increase in expression in BMSC but a decrease in expression in ASC during adipogenic differentiation and a larger increase in BMSC vs. ASC during osteogenesis. As for the cluster 14, the cluster 11 was highly enriched with genes related to extracellular region, in particular signaling molecules (Figure 7 and File S8), indicating a large co-regulation of cell-to-cell communication during osteogenesis.

Few clusters showed a similar pattern between adipogenic and osteogenic differentiation. Clusters 1, 3, 8 and 13 (and with lower magnitude cluster 5) grouped genes with, on average, a consistent down-regulation during differentiations but with a larger decrease in adipogenic compared to osteogenic differentiation. Interestingly, those clusters were highly enriched with genes related to cytoskeleton organization, regulation of organelle organization, heparin binding, and focal adhesion (Figure 7 and File S7). The cytoskeleton plays a pivotal role in cell shape, organelle organization, polarity, and sensing external forces that in turn are able to stimulate differentiation. This has been shown in BMSC [[5](#_ENREF_5)] but also in ASC [[6](#_ENREF_6)]. The coordinated down-regulation of the cytoskeleton during differentiation, particularly for the adipogenic differentiation, might be indicative of decreased cell interactions but might also indicate a decreased hypersensitivity of the cells to the stiffness of the surrounding environment. In human BMSC the phosphorylation of the actin cytoskeleton is an important phenomenon during *in vitro* osteogenesis [[7](#_ENREF_7)]. Increase in focal adhesion after mechanical stimulation has been shown to be involved in inhibiting adipogenesis in murine BMSC [[8](#_ENREF_8)]. Our data confirm those results by showing a strong decrease in expression of genes involved in focal adhesion due to differentiations (cluster 3 in Figure 7). The KEGG pathway analysis using DIA also indicated that the ‘Focal adhesion’ pathway was overall inhibited during adipogenesis and was slightly induced during osteogenesis, particularly for BMSC (Figure 4).

Among the other clusters few had enrichment of genes related to particular biological terms. Cluster 6, featured an overall down-regulation during differentiation and a larger decrease during osteogenesis compared to adipogenesis but was highly enriched in genes involved in protein synthesis. In agreement with this observation, the ‘Ribosome’ KEGG pathway was inhibited overall during differentiation, particularly during the osteogenic differentiation (Figure 3).

The clusters 2, 9 and 10 grouped genes that had a similar behavior between differentiation types but a larger increase in expression in ASC vs. BMSC for cluster 2 and BMSC vs. ASC for clusters 9 and 10 (Figure 7). Cluster 2 was highly enriched in genes involved in collagen binding and extracellular region signaling encompassing cell adhesion and fibronectin (File S7 and File S8). Those data indicated that the porcine ASC during osteogenesis had a stronger cell-to-cell signaling and interactions compared to BMSC. This might partly explain the formation of large nodules in ASC compared to the uniform layer in BMSC observed during 2D *in vitro* osteogenesis [[9](#_ENREF_9)]. The clusters 9 and 10 were not enriched with genes related to particular biological terms (File S7 and File S8); however, using the liberal EASE score the most enriched genes in cluster 9 were the ones involved in extracellular matrix binding and in cluster 10 were the genes involved in posttranscriptional regulation and lipid biosynthesis, particularly cholesterol biosynthesis. Greater steroidogenesis in BMSC during either differentiation type is consistent with what was suggested by the DIA results of the analysis of the pathways (see Figure 3).

The cluster 16 grouped genes that had a very large increase in expression in both differentiation types (Figure 7). This cluster was highly enriched in genes involved in extracellular matrix and cell-to-cell interaction through glycoproteins (File S7 and File S8). This indicates that in both differentiation types minimal interaction between cells is essential and needs to be highly coordinated, even though overall cell-to-cell interaction appeared to be inhibited during adipogenesis (see above).

The transcription factor network analysis (Figure S14) indicated that genes present in cluster 13, followed by cluster 4 and 8, formed the largest interactive networks; however, cluster 4 had the largest number of transcription factor (TF) in the network and also the largest number of TF both present in the cluster but also putative TF with >3 down-stream genes (see caption of Figure S14). This indicates that the transcription of genes in cluster 4 was highly coordinated but also that the transcription involved harmonization of a large number of TF (see File S9 for details).

The statistical analysis of overlap TF performed using Ingenuity Pathway Analysis (Table 1, all the results are available in File S10) supports the above observations. The cluster 4 had the largest number of putative TF, considering a stringent significance, with several TF previously known to be involved in adipogenesis, such as PPARγ and CEBPα [[10](#_ENREF_10),[11](#_ENREF_11),[12](#_ENREF_12)] (Table 1). Also, activation of PPARα has been reported to induce adipogenesis [[13](#_ENREF_13)].

The cluster 3 also had a large number of significant overlapping TF (Table 1 and Figure S14). It is interesting that two of the TF with the most significant overlap (i.e., up-stream transcription factor) with the genes in cluster 3 were up-regulated during adipogenesis. For instance *FOS* was present in cluster 4 and *JUN* in cluster 7 (File S1 and File S9). It has been shown that expression of *FOS* increases during adipogenesis but, contrary to the increase in expression during adipogenesis observed in the present experiment (File S1), not *JUN* [[12](#_ENREF_12)]. Those observations suggest coordination between induction of lipid accumulation (i.e., cluster 4) and inhibition of focal adhesion (cluster 3).

Overall the cluster analysis in association with the TF network and TF overlapping analyses strongly indicated large transcriptomics coordination and large interactions of genes involved in adipogenic compared to osteogenic differentiation. In addition, the analysis highlighted a larger number of TF involved in driving adipogenesis compared to osteogenesis.

Few TF are known to have a large amount of down-stream target genes, such as MYC (encompassing several isoforms) and TP53. As consequence, those TF have >3 putative target genes in all the 16 gene clusters (File S10). The enrichment analysis using IPA indicated that only few clusters had a significant enrichment of target genes for MYC (and its isoforms) and/or TP53 (Table 1). Among those, cluster 6 was highly enriched by genes involved in protein synthesis and was also highly enriched by target genes of MYCN or n-Myc (Table 1). The n-Myc is part of a family of transcription factors having similar functions, among those the v-Myc (or simply MYC) has been shown to play a crucial role in coordinating expression of ribosomal proteins that are involved in protein synthesis [[19](#_ENREF_19)]. In our experiment, *MYC* was actually up-regulated during adipogenesis while *MYCN* was not affected by differentiations (File S1). Although not clear due to the increase or not change in expression, our analysis suggest that MYC had likely played a role in coordinating the decrease in protein synthesis during osteogenic differentiations in our experiment with a probable more important role of n-Myc than v-Myc.

The TP53 is one of the most studied transcription factors for its role in tumor suppression due to its anti-proliferative property [[20](#_ENREF_20)]. This TF plays an important role in inhibiting bone differentiation and it also regulates the balance between bone formation and bone resorption [[21](#_ENREF_21)]. Interestingly, cluster 3 is highly enriched by target genes of TP53 (Table 1). The genes in this cluster are featured by an overall down-regulation during differentiation, with a larger down-regulation during adipogenesis compared to osteogenesis. The expression of *TP53* gene was not affected by the conditions in the present study (File S1). In summary, the data indicated that TP53 played an important role in coordinating the expression of several genes in our study with an apparent suppressive role in expression that might be important for allowing differentiation in both lineages.

**References**

1. Ducluzeau PH, Priou M, Weitheimer M, Flamment M, Duluc L, et al. (2011) Dynamic regulation of mitochondrial network and oxidative functions during 3T3-L1 fat cell differentiation. Journal of physiology and biochemistry 67: 285-296.

2. Liu D, Lin Y, Kang T, Huang B, Xu W, et al. (2012) Mitochondrial Dysfunction and Adipogenic Reduction by Prohibitin Silencing in 3T3-L1 Cells. PloS one 7: e34315.

3. Tzaphlidou M (2005) The role of collagen in bone structure: an image processing approach. Micron 36: 593-601.

4. Heining E, Bhushan R, Paarmann P, Henis YI, Knaus P (2011) Spatial segregation of BMP/Smad signaling affects osteoblast differentiation in C2C12 cells. PloS one 6: e25163.

5. Engler AJ, Sen S, Sweeney HL, Discher DE (2006) Matrix elasticity directs stem cell lineage specification. Cell 126: 677-689.

6. Choi YS, Vincent LG, Lee AR, Dobke MK, Engler AJ (2012) Mechanical derivation of functional myotubes from adipose-derived stem cells. Biomaterials 33: 2482-2491.

7. Lo T, Tsai CF, Shih YR, Wang YT, Lu SC, et al. (2012) Phosphoproteomic analysis of human mesenchymal stromal cells during osteogenic differentiation. Journal of proteome research 11: 586-598.

8. Sen B, Guilluy C, Xie Z, Case N, Styner M, et al. (2011) Mechanically induced focal adhesion assembly amplifies anti-adipogenic pathways in mesenchymal stem cells. Stem cells 29: 1829-1836.

9. Monaco E, Sobreira de Lima A, Bionaz M, Maki AJ, Wilson SW, et al. (2009) Morphological and Transcriptomic Comparison of Adipose and Bone Marrow Derived Porcine Stem Cells. The Open Tissue Engineering & Regenerative Medicine Journal: 20-33.

10. Grontved L, Madsen MS, Boergesen M, Roeder RG, Mandrup S (2010) MED14 tethers mediator to the N-terminal domain of peroxisome proliferator-activated receptor gamma and is required for full transcriptional activity and adipogenesis. Molecular and cellular biology 30: 2155-2169.

11. Muruganandan S, Roman AA, Sinal CJ (2009) Adipocyte differentiation of bone marrow-derived mesenchymal stem cells: cross talk with the osteoblastogenic program. Cellular and molecular life sciences : CMLS 66: 236-253.

12. Rangwala SM, Lazar MA (2000) Transcriptional control of adipogenesis. Annual review of nutrition 20: 535-559.

13. Spiegelman BM, Hu E, Kim JB, Brun R (1997) PPAR gamma and the control of adipogenesis. Biochimie 79: 111-112.

14. Banerjee SS, Feinberg MW, Watanabe M, Gray S, Haspel RL, et al. (2003) The Kruppel-like factor KLF2 inhibits peroxisome proliferator-activated receptor-gamma expression and adipogenesis. The Journal of biological chemistry 278: 2581-2584.

15. Foryst-Ludwig A, Clemenz M, Hohmann S, Hartge M, Sprang C, et al. (2008) Metabolic actions of estrogen receptor beta (ERbeta) are mediated by a negative cross-talk with PPARgamma. PLoS genetics 4: e1000108.

16. Song B, Estrada KD, Lyons KM (2009) Smad signaling in skeletal development and regeneration. Cytokine & growth factor reviews 20: 379-388.

17. Collins A, Hewitt SL, Chaumeil J, Sellars M, Micsinai M, et al. (2011) RUNX transcription factor-mediated association of Cd4 and Cd8 enables coordinate gene regulation. Immunity 34: 303-314.

18. Monaco E, Bionaz M, Sobreira de Lima A, Hurley WL, Loor JJ, et al. (2010) Selection and reliability of internal reference genes for quantitative PCR verification of transcriptomics during the differentiation process of porcine adult mesenchymal stem cells. Stem cell research & therapy 1: 7.

19. van Riggelen J, Yetil A, Felsher DW (2010) MYC as a regulator of ribosome biogenesis and protein synthesis. Nature reviews Cancer 10: 301-309.

20. Seemann S, Maurici D, Olivier M, Caron de Fromentel C, Hainaut P (2004) The tumor suppressor gene TP53: implications for cancer management and therapy. Critical reviews in clinical laboratory sciences 41: 551-583.

21. Liu H, Li B (2010) p53 control of bone remodeling. Journal of cellular biochemistry 111: 529-534.
